# AGR2-mediated unconventional secretion of 14-3-3ε and α-actinin-4, responsive to ER stress and autophagy, drives chemotaxis in canine mammary tumor cells

**DOI:** 10.1186/s11658-024-00601-w

**Published:** 2024-05-31

**Authors:** Stephen Hsien-Chi Yuan, Chih-Ching Wu, Yu-Chih Wang, Xiu-Ya Chan, Hao-Wei Chu, Youngsen Yang, Hao-Ping Liu

**Affiliations:** 1 Department of Veterinary Medicine, College of Veterinary Medicine, National Chung Hsing University, Taichung, 40227 Taiwan; 2 Graduate Institute of Biomedical Sciences, College of Medicine, Chang Gung University, Taoyuan, Taiwan; 3 Department of Otolaryngology-Head and Neck Surgery, Chang Gung Memorial Hospital, Taoyuan, Taiwan; 4 Molecular Medicine Research Center, Chang Gung University, Taoyuan, Taiwan; 5 Department of Medical Biotechnology and Laboratory Sciences, College of Medicine, Chang Gung University, Taoyuan, Taiwan; 6 Research Center for Emerging Viral Infections, College of Medicine, Chang Gung University, Taoyuan, Taiwan; 7 Graduate Institute of Veterinary Pathology, College of Veterinary Medicine, National Chung Hsing University, Taichung, 40227 Taiwan; 8 Department of Oncology, Taichung Veterans General Hospital, Taichung, Taiwan; 9 Biotechnology Center, National Chung Hsing University, Taichung, 40227 Taiwan

**Keywords:** Canine mammary tumor (CMT), Anterior gradient 2 (AGR2), 14-3-3 Epsilon (YWHAE), Alpha-actinin 4 (ACTN4), Proteomics, Chemotaxis, Unconventional protein secretion, Microenvironment

## Abstract

**Background:**

Canine mammary tumors (CMTs) in intact female dogs provide a natural model for investigating metastatic human cancers. Our prior research identified elevated expression of Anterior Gradient 2 (AGR2), a protein disulfide isomerase (PDI) primarily found in the endoplasmic reticulum (ER), in CMT tissues, highly associated with CMT progression. We further demonstrated that increased AGR2 expression actively influences the extracellular microenvironment, promoting chemotaxis in CMT cells. Unraveling the underlying mechanisms is crucial for assessing the potential of therapeutically targeting AGR2 as a strategy to inhibit a pro-metastatic microenvironment and impede tumor metastasis.

**Methods:**

To identify the AGR2-modulated secretome, we employed proteomics analysis of the conditioned media (CM) from two CMT cell lines ectopically expressing AGR2, compared with corresponding vector-expressing controls. AGR2-regulated release of 14-3-3ε (gene: YWHAE) and α-actinin 4 (gene: ACTN4) was validated through ectopic expression, knockdown, and knockout of the AGR2 gene in CMT cells. Extracellular vesicles derived from CMT cells were isolated using either differential ultracentrifugation or size exclusion chromatography. The roles of 14-3-3ε and α-actinin 4 in the chemotaxis driven by the AGR2-modulated CM were investigated through gene knockdown, antibody-mediated interference, and recombinant protein supplement. Furthermore, the clinical relevance of the release of 14-3-3ε and α-actinin 4 was assessed using CMT tissue-immersed saline and sera from CMT-afflicted dogs.

**Results:**

Proteomics analysis of the AGR2-modulated secretome revealed increased abundance in 14-3-3ε and α-actinin 4. Ectopic expression of AGR2 significantly increased the release of 14-3-3ε and α-actinin 4 in the CM. Conversely, knockdown or knockout of AGR2 expression remarkably reduced their release. Silencing 14-3-3ε or α-actinin 4 expression diminished the chemotaxis driven by AGR2-modulated CM. Furthermore, AGR2 controls the release of 14-3-3ε and α-actinin 4 primarily via non-vesicular routes, responding to the endoplasmic reticulum (ER) stress and autophagy activation. Knockout of AGR2 resulted in increased α-actinin 4 accumulation and impaired 14-3-3ε translocation in autophagosomes. Depletion of extracellular 14-3-3ε or α-actinin 4 reduced the chemotaxis driven by AGR2-modulated CM, whereas supplement with recombinant 14-3-3ε in the CM enhanced the CM-driven chemotaxis. Notably, elevated levels of 14-3-3ε or α-actinin 4 were observed in CMT tissue-immersed saline compared with paired non-tumor samples and in the sera of CMT dogs compared with healthy dogs.

**Conclusion:**

This study elucidates AGR2’s pivotal role in orchestrating unconventional secretion of 14-3-3ε and α-actinin 4 from CMT cells, thereby contributing to paracrine-mediated chemotaxis. The insight into the intricate interplay between AGR2-involved ER stress, autophagy, and unconventional secretion provides a foundation for refining strategies aimed at impeding metastasis in both canine mammary tumors and potentially human cancers.

**Supplementary Information:**

The online version contains supplementary material available at 10.1186/s11658-024-00601-w.

## Introduction

Comparative oncology, a burgeoning field dedicated to investigating cancer risk and tumor development across various species, aims to advance human and animal health [1, 2]. By investigating naturally occurring cancers in pet dogs, we gain valuable insights that enhance our understanding and management of relevant human cancers. Among these, canine mammary tumors (CMTs) stand out as the most prevalent neoplasms, constituting 50–70% of tumors in intact female dogs, with approximately 50% being malignant [3, 4].

Malignant CMTs predominantly originate from epithelial tissues and exhibit diverse histological subtypes, including the most common complex carcinoma (11%) and simple carcinoma (11%) [5, 6]. Complex carcinoma consists of both malignant glandular epithelial cells and benign myoepithelial cells, while simple carcinoma comprises a single neoplastic cell type, which mirrors molecular aspects of human breast carcinomas and often signifies a worse prognosis [7]. Molecular categorization, based on expression statuses of estrogen receptor (ER) and human epidermal growth factor receptor 2 (HER2), classifies CMT into four subtypes: luminal A (ER^+^/HER2^−^; 44.8%), luminal B (ER^+^/HER2^+^; 13.5%), basal (ER^−^/HER2^−^ and a basal marker positive; 29.2%), and HER2-overexpressing (ER^−^/HER2^+^; 8.3%) [8]. Dogs with luminal B tumors exhibit higher mortality, metastasis, and recurrence rates in the liver or lungs compared to those with luminal A tumors. Additionally, HER2-overexpressing cancers tend to present with high-grade histology and aggressive clinical behaviors [8].

Long-term studies indicate that the risk of CMT-associated mortality within two years of diagnosis ranges from 20 to 45% [9]. Notably, exposure to ovarian hormones is a significant risk factor in CMT development [10]. Certain breeds, such as Beagles and Maltese, demonstrate a propensity for CMT, while these tendencies may vary geographically [11, 12]. Despite the prevalence of CMT, the underlying molecular mechanisms driving CMT pathogenesis remain largely uncharacterized.

To unravel novel proteins associated with CMT pathogenesis, we employed quantitative proteomic approaches to conduct a comprehensive analysis of the proteomes in CMT tissues, along with matching hyperplastic and normal mammary gland tissues [13]. This investigation led to the identification of Anterior gradient 2 (AGR2) as highly expressed in CMT tissues. Subsequent analyses conclusively demonstrated significant overexpression of AGR2 in various histopathological subtypes of CMT tissues, contrasting sharply with its expression levels in normal or hyperplastic mammary gland tissues [14]. AGR2 is an ortholog to the *Xenopus laevis* protein XAG-2, which plays an essential role in the development of the forebrain and the mucus-secreting cement gland [15]. Notably, canine AGR2 shares 94% of its peptide sequence with human AGR2.

AGR2 functions as a protein disulfide isomerase (PDI) primarily located in the endoplasmic reticulum (ER). It mediates the formation of disulfide bonds, catalyzes the cysteine-based redox reactions, and adds to the quality control of proteins [16, 17]. AGR2 is indispensable for the production of the intestinal mucin MUC-2 [18]. Additionally, AGR2 engages with MUC-5 as a primary client and is co-expressed with the acidic mucin in Barrett’s esophagus and esophageal adenocarcinoma tissue [19]. Furthermore, AGR2 binding to the epidermal growth factor receptor (EGFR) in the ER is crucial for the delivery of EGFR to the plasma membrane [20].

Dysregulation of PDIs can result in the accumulation of misfolded client proteins in the ER, triggering ER stress and unfolded protein response (UPR), consequently contributing to the development of various diseases [21–23]. AGR2 dysregulation has been linked to a range of diseases, including inflammatory bowel diseases [24], neoplasm development, and cancer progression [17, 25–27]. Overexpression of AGR2 is commonly observed in various human adenocarcinomas, where it promotes tumor growth and metastasis, correlating with a poor prognosis [28–30]. Moreover, upregulation of AGR2 expression is seen in chemotherapeutics-resistant cancer cells, further contributing to drug resistance [31–34].

Notably, AGR2 encompasses an N-terminal signal sequence and can be secreted from both non-tumor and tumor cells into the extracellular milieu through the conventional secretory pathway [24, 25, 35–40]. Elevated levels of extracellular AGR2 have been detected in sera, plasma, or urine of human cancer patients, including those with breast cancer, prostate cancer, urothelial carcinoma, nasopharyngeal carcinoma, and ovarian cancer [32, 41–45], showing a positive correlation with metastasis occurrence and poor prognosis. Along these lines, our previous study demonstrated a significant association between serum AGR2 concentration in CMT-afflicted dogs and distant tumor metastasis, as well as worse overall survival [14], suggesting the potential utility of serum AGR2 as a prognostic indicator for CMT. Moreover, our findings reinforce the suitability of CMT as a complementary model for investigating AGR2 function in human cancer [46, 47]. Significantly, the carcinogenic properties of extracellular AGR2 underscore its potential as a therapeutic target [46]. Monoclonal antibodies specifically targeting the extracellular AGR2 have shown promising results in human cancers [37, 48, 49].

In this study, we discovered that AGR2, in addition to being secreted, can regulate the release of 14-3-3ε (YWHAE) and alpha-actinin 4 (ACTN4) from CMT cells. Subsequently, the released 14-3-3ε and α-actinin 4 play a pivotal role in promoting the paracrine chemotaxis of CMT cells.

14-3-3ε is an isoform of the 14-3-3 family of conserved regulatory adaptor molecules expressed in all eukaryotic cells. By recognizing specific phosphorylation motifs and interacting with target proteins, 14-3-3 proteins participate in a variety of intracellular processes, regulate the subcellular localization of target proteins [50], and act as a stress-adaptive signaling hub in cancer cells that governs critical processes, including apoptosis, cell cycle progression, autophagy, glucose metabolism, and cell motility [51, 52]. Notably, 14-3-3ε has been reported as an oncogene in various cancer cell lines [51] and viewed as a target to modulate drug sensitivity [53–55].

α-actinin 4 belongs to a family of actin-binding proteins and is a non-muscle alpha-actinin that has long been associated with cancer development and metastasis [56, 57]. α-actinin 4 is predominantly expressed in the cellular protrusions that stimulate the invasive phenotype in cancer cells, and overexpression of α-actinin 4 has been noticed to parallel the accretion of drug resistance in several cancer cell lines [58].

Despite lacking a signal sequence required for conventional protein secretion through the ER-to-Golgi route, 14-3-3ε and α-actinin 4 have been identified in the secretomes of various human cancer cell lines [59, 60] and extracellular vesicles (EVs) originating from human cancers [61–64]. Moreover, 14-3-3ε and α-actinin 4 may be released directly from the cell membranes or liberated from the EVs to serve as ligands for cell-surface proteins. For instance, 14-3-3ε has been found to function as a soluble mediator critical in the communication between subchondral bone and cartilage in osteoarthritis [65]. Similarly, elevated levels of serum α-actinin 4 are associated with lymph node metastasis and a poorer prognosis in cervical cancer [66].

While intracellular 14-3-3ε and α-actinin 4 have been implicated in cancer progression, their extracellular pro-oncogenic roles have not yet been elucidated. Herein we aimed to characterize how AGR2 controls the release of 14-3-3ε and α-actinin 4 using CMT cells as a model system. To our knowledge, this is the first investigation into the AGR2-modulated secretome, involving the unconventional secretion of oncogenic proteins 14-3-3ε and α-actinin 4. Understanding the mechanisms underlying the AGR2-controlled release of 14-3-3ε and α-actinin 4 in response to stress conditions will contribute to the refinement of AGR2-targeting therapeutic strategies for cancers.

## Materials and methods

### Cell lines

Four CMT cell lines (CMT-U27, CMT-U27e, CF41.Mg, and DMGT), along with two human breast cancer cell lines (MDA-MB-231 and MCF7) were utilized in this study. CMT-U27 and CF41.Mg were purchased from American type culture collection (ATCC; CRL-3456™ and CRL-6232TM, respectively). CMT-U27 originated from a simple carcinoma-subtype CMT obtained from a 14-year-old female Poodle, while CF41.Mg was derived from a mixed-type CMT acquired from a 10-year-old female Beagle. DMGT originated from a complex carcinoma-subtype CMT obtained from a six-year-old female crossbreed dog, originally established by Dr. Shih-Chieh Chang’s team at the Department of Veterinary Medicine, National Chung Hsing University. The identities of CMT cell lines were confirmed by short tandem repeat (STR) profiling analysis.

CMT-U27e, a subline derived from CMT-U27, was obtained after mock selection in parallel with puromycin selection of AGR2-knockout and control CMT-U27 clones (see below). It exhibits elevated levels of AGR2 compared to the original cell line. CMT-U27 and its derivatives were maintained in Roswell Park Memorial Institute 1640 medium (RPMI; Gibco, Thermo Fisher Scientific), while CF41.Mg, DMGT, MDA-MB-231, and MCF7 were maintained in Dulbecco’s Modified Eagle’s Medium (DMEM; Gibco, Thermo Fisher Scientific) with high glucose. For maintenance, cells were grown in culture media supplemented with 10% fetal bovine serum (FBS; Gibco, Thermo Fisher Scientific) at 37 °C in an incubator supplied with 5% CO_2_.

### Transfection

For ectopic plasmid transfection, cells were seeded in 6-well plates at the following densities: CMT-U27 (4.5 × 10^5^ cells/well), CF41.Mg (2 × 10^5^ cells/well), DMGT (3 × 10^5^ cells/well), MDA-MB-231 (3 × 10^5^ cells/well), or MCF7 (3 × 10^5^ cells/well). The cells were allowed to grow for 18 to 24 h before transfection. CMT-U27, DMGT, MDA-MB-231, or MCF7 was transfected with 1.5 µg of plasmids or the mock control using 4.5 µL of Lipofectamine™ 2000 (Invitrogen, Thermo Fisher Scientific). CF41.Mg was transfected with 2 µg of plasmids using 4 µL of jetPRIME^®^ (Polyplus, Illkirch, France) according to the manufacturer’s instruction.

For siRNA transfection, CMT-U27 or CMT-U27e was transfected with 75 nM siRNA oligonucleotide duplexes using 10 µL of Lipofectamine™ RNAiMAX (Invitrogen, Thermo Fisher Scientific). Following media replacement, transfected cells were cultured for the specified time intervals before further experiments.

### AGR2-expressing vectors

The expression vector for canine AGR2 tagged with Myc and His was previously constructed in a pcDNA3.1(+)-A-myc.His backbone [14], denoted pcDNA3.1-myc.His-AGR2 throughout the text. The expression vector for HA-tagged AGR2 was generated by integrating the DNA fragment encoding canine AGR2 into a pcDNA3.1(+)-HA-C vector via the KpnI and XhoI site. To generate the expression vector for human AGR2, the DNA fragment encoding human AGR2 was obtained from human A549 cells using similar methods and then inserted into a pcDNA3.1(+)-A-myc.His vector, referred to as pcDNA3.1-myc.His-hAGR2 in the text.

### siRNA oligonucleotide duplexes

Duplex oligonucleotides of siRNA against canine *AGR2* (denoted siAGR2) were synthesized by and purchased from GeneDireX Inc. (Taoyuan, Taiwan). Sequences of two siAGR2 duplexes are listed as follows: siAGR2-1: 5ʹ-GGCCAAAGAUAUCACAGUUTT-3ʹ and 5ʹ-AACUGUGAUAUAUCUUUGGCCTT-3ʹ; siAGR2-2: 5ʹ-GACUCAGACCUAUGAAGAATT-3ʹ and 5ʹ-UUCUUCAUAGGUCUGAGUCTT-3ʹ.

siRNA duplexes against canine *YWHAE* (denoted siYWHAE) or those against canine *ACTN4* (denoted siACTN4) were synthesized by and purchased from Eurogentec (Seraing, Liège, Belgium). Sequences of two siYWHAE and siACTN4 duplexes are listed as follows: siYWHAE-1: 5ʹ-GUUGACAGUUGAAGAAAGATT-3ʹ and 5ʹ-UCUUUCUUCAACUGUCAACTT-3ʹ; siYWHAE-2: 5ʹ-CAAGGGAGGAGAAGACAAATT-3ʹ and 5ʹ-UUUGUCUUCUCCUCCCUUG-3ʹ; siACTN4-1: 5ʹ-CCUUCCAAGCCUUCAUUGATT-3’ and 5ʹ-UCAAUGAAGGCUUGGAAGGTT-3ʹ; siACTN4-2: 5ʹ-GUUGACAGUUGAAGAAAGATT-3ʹ and 5ʹ-UCUUUCUUCAACUGUCAACTT-3ʹ.

### Generation of AGR2-knockout (KO) CMT cell clones

The CRISPR-Cas9 technique was used to generate AGR2-KO cells in CMT-U27 by transient transfection method. Single guide RNA (sgRNAs) targeting canine *AGR2*, i.e., canine AGR2 sgRNA-1 or AGR2 sgRNA-2, was constructed into the pU6-gRNA.Ppuro vector by the National RNAi Core Facility, Academia Sinica Taiwan (Nankang, Taipei, Taiwan). AGR2 sgRNA-1 and AGR2 sgRNA-2 sequences are as follows: 5ʹ-GAGTGTAAGAGAGGGCGACG-3ʹ and 5ʹ-GTTGGCCAGAGTGTAAGAGA-3ʹ, respectively. CMT-U27 cells were transfected with the plasmid expressing AGR2 sgRNA-1, AGR2 sgRNA-2, or a mock control using Lipofectamin™ 2000 as described above. The transfectants were selected with 1 μg/mL puromycin at 24 h post-transfection for four days. Live cells were serially diluted and seeded into a 96-well culture plate, then grown until a single cell clone was obtained. Two AGR2-KO clones, KO-S10 and KO-S4, were generated using AGR2 sgRNA-1 and AGR2 sgRNA-2, respectively. The null expression of AGR2 was verified by immunoblotting.

### Collection of conditioned media and cell lysates

Conditioned media (CM) were first centrifuged at 300 × *g* for 10 min to remove detached cells and further centrifuged at 3000 × *g* for 10 min to remove cell debris. Collected CM was used for following experiments or applied to further processes as indicated elsewhere. Cell lysates were harvested in a homogenization buffer (20 mM Tris–HCl, pH 7.5, 150 mM NaCl, 5 mM EDTA, pH 8.0, 1% NP-40) supplemented with the protease inhibitor cocktail (VWR Life Science, USA) and phenyl methyl sulfonyl fluoride (PMSF; Sigma-Aldrich, USA). Protein concentrations of the CM and cell lysates were measured using a BCA protein concentration assay kit (Pierce, Thermo Fisher Scientific) according to the manufacturer’s instructions.

### Transwell migration assay

Conditioned media (CM) collected from corresponding cells subjected to various treatments, or fresh media supplemented with recombinant proteins, were added to the bottom well as the attractant (600 µL per well). Cells were suspended in 160 µL of serum-free culture media, stained with 40 µL of Hoechst 33342 Staining Dye Solution (500 nM; Abcam, UK), and then placed in a top hanging insert with a pore size of 8 μm (SPL, Korea) for a transwell migration assay. Cells were seeded into the insert as follows: CMT-U27 (9 × 10^4^ cells), CMT-U27e (9 × 10^4^ cells), CF41.Mg (6 × 10^4^ cells), DMGT (6 × 10^4^ cells), MDA-MB-231 (1 × 10^5^ cells), or MCF7 (1 × 10^5^ cells). Following a 16- to 20-h incubation at 37 °C, cells in the inserts were fixed with 10% formalin at room temperature for 30 min and then washed several times with 1 × PBS. Cells that remained inside the insert (non-migrated cells) were removed with a cotton swap. Images of migrated cells were acquired by using the Leica DMI3000 B Inverted Microscope (Leica, Wetzlar, Germany) equipped with a Zyla 5.5 Megapixel sCMOS camera (Andor Technology, Belfas, Ireland) under a 10 × objective. The number of migrated cells was measured with MetaMorph^®^ NX Software (Molecular Devices, San Jose, CA, USA) and presented as the mean + SD of three independent experiments.

### Proteomics analysis of the AGR2-affected CMT secretome by a gel-enhanced liquid chromatography-tandem mass spectrometry (GeLC-MS/MS)

Serum-free CM samples collected from transfected CMT cells were deprived of cells and cell debris and subsequently concentrated and desalted with Vivaspin^®^ 20 (GE Healthcare) following the manufacturer’s instructions. Concentrated CM proteins (10 µg) were resolved by sodium dodecyl sulfate–polyacrylamide gel electrophoresis (SDS-PAGE) and stained with 0.5% Coomassie Brilliant Blue G-250 (AppliChem GmbH, Germany). Individual gel lanes were cut into 15 pieces, each of which was dehydrated in acetonitrile (Mallinckrodt Baker) and dried using SpeedVac. The proteins were reduced with 25 mM NH_4_HCO_3_ (Sigma-Aldrich) containing 10 mM dithiothreitol (Biosynth AG, Switzerland) at 60 °C for 30 min and alkylated with 55 mM iodoacetamide (Amersham Biosciences, UK) at room temperature for 30 min. The proteins were then digested with trypsin (20 μg/mL; Thermo Fisher Scientific) overnight at 37 °C.

The extracted peptides were analyzed by the LTQ-Orbitrap Discovery (Thermo Fisher Scientific) as described in the previous study [59]. Briefly, peptide extracts were reconstituted in HPLC buffer A (0.1% formic acid; Sigma-Aldrich), loaded across a trap column (Zorbax 300SB-C_18_, 0.3 × 5 mm; Agilent Technologies, Taiwan) at a flow rate of 0.2 µL/min in HPLC buffer A, and separated on a resolving 100 mm analytical C18 column (inner diameter, 75 µm) using a 15-µm tip (New Objective, USA). The peptides were eluted with a 60-min gradient of HPLC buffer B at a flow rate of 0.25 µL/min across the analytical column. Data-dependent mode was used to detect intact peptides at a resolution of 30,000 and 10 MS/MS scans for the 10 most abundant precursor ions were used to acquire data.

For database searching, the obtained MS/MS spectra were analyzed using the Mascot algorithm (version 2.2.04; Matrix Science, Boston, MA, USA). The search was conducted against the Canis sequence database of Swiss-Prot (released on March 2021; selected for Canis lupus familiaris, 25,491 entries) from the European Bioinformatics Institute. The fragment ion mass tolerance was set to 0.5 Da and the parent ion mass tolerance was set to 10 ppm, with trypsin as the digestion enzyme. Up to one missed cleavage was allowed, and searches included the parameters for variable oxidation on methionine (+ 15.99 Da) and fixed carbamidomethylation on cysteine (+ 57 Da). A random sequence database was used to estimate false-positive rates for peptide matches.

### The label-free MS quantification

To identify the AGR2-affected secretome, the label-free MS quantification was used to compare the abundance of CM proteins derived from AGR2-expressing CMT cells with those derived from the vector-expressing control. Each CM sample was meticulously analyzed in triplicate, represented as Rep1, Rep2, and Rep3. The quantification of protein abundance was accomplished by calculating the ratio of the peptide-spectrum match (PSM) count attributed to a specific protein against the PSM counts of all identified proteins within the separate replicates. The cumulative protein abundance, represented as the normalized PSM ratio, within AGR2-expressing CM triplicates was divided by the corresponding cumulative value within vector-expressing CM triplicates. This ratio underwent a logarithmic transformation (Log2), denoted as Log2 (A/V). Proteins that displayed a Log2 (A/V) exceeding the mean + 1.5 SD were classified as AGR2-increased, while those falling below the mean − 1.5 SD were designated as AGR2-decreased.

### Immunoprecipitation

Depletion of Myc-tagged AGR2, 14-3-3ε, and α-actinin 4 in the CM was conducted by immunoprecipitation (IP). For depletion of Myc-tagged AGR2, CM of CMT-U27 transfected with an AGR2-expressing vector was incubated with Myc-Trap^®^ (ChromoTek, Germany) according to the manufacturer’s protocol. The slurry of Myc-Trap^®^ beads (25 µL per reaction) was equilibrated with dilution buffer (10 mM Tris–HCl, pH 7.5, 150 mM NaCl, 0.5 mM EDTA) three times, and the equilibrated beads were incubated with 600 µL of CM and rotated end-over-end at 4 °C for 1 h. For the depletion of 14-3-3ε or α-actinin 4 in the CM, antibodies (3 μg) against 14-3-3ε or α-actinin 4 were first incubated with Dynabeads™ Protein G (40 µL per reaction; Thermo Fisher Scientific) and rotated end-over-end at room temperature for 20 min. Subsequently, Dynabeads™ Protein G-immobilized antibodies were incubated with 600 µL of CM and rotated end-over-end at 4 °C for 16 h. The beads were retained with a magnet, and the remained CM was collected and used for a transwell migration assay. The beads were washed three times with wash buffer (10 mM Tris–HCl, pH 7.5, 150 mM NaCl, 0.5 mM EDTA, 0.05% Triton X-100), and the IP complexes were resolved with 30 µL of 2 × sampling buffer (100 mM Tris–HCl, pH 6.8, 2% β-mercaptoethanol, 4% SDS, 20% glycerol, 0.04% bromophenol blue, 100 mM EDTA) for immunoblotting analysis.

### Trichloroacetic acid precipitation of CM proteins

Trichloroacetic acid (TCA; Merck KGaA, Germany) was used to precipitate proteins in the 1–2% FBS-containing CM that was applied to a transwell migration assay. CM was mixed with TCA (20%v/v) and incubated at 4 °C overnight. The mixture was centrifuged at 14,000 rpm at 4 °C for 30 min. Precipitated protein pellets were washed with 200 μL of ice-cold acetone (Merck KGaA, Germany) twice to remove residue of TCA, and then air-dried and resuspended in a sample solution (1% sodium dodecyl sulfate (SDS), 10 mM EDTA, pH 8.0). Resuspended samples were further sonicated at 37 °C for 1 h to thoroughly dissolve the protein pellets.

### Immunoblotting

Protein extracts resolved in 1 × sampling buffer (50 mM Tris–HCl, 1% ꞵ-mercaptoethanol, 2% SDS, 10% glycerol, 0.02% bromophenol blue, 50 mM EDTA, pH 6.8) were boiled at 95 °C for 10 min, and then separated by SDS-PAGE with 9% to 15% gradient polyacrylamide gels. Protein samples were transferred to POLYSCREEN^®^ polyvinylidene difluoride (PVDF) membranes (PerkinElmer Life Science, Inc., Boston, MA, USA), blocked with BlockPRO™ Blocking Buffer (Visual Protein, Taiwan) at room temperature for 1 h, and subsequently incubated with appropriately diluted primary antibodies (listed in Table 1) at 4 °C overnight.

**Table 1:** Primary antibodies used for immunoblotting in this study

Antibody against	Cat. No	Supplier	Dilution
AGR2	ab209224	Abcam, UK	1:1000
E-cadherin	610182	BD Bioscience, USA	1:1000
Vimentin (E-5)	sc-373717	Santa Cruz Biotechnology, Inc., USA	1:1000
HER-2	MA5-13105	Invitrogen, Thermo Fisher Scientific, USA	1:1000
ER-α	MA5-13065	Invitrogen, Thermo Fisher Scientific, USA	1:1000
β-tubulin	66240-1-Ig	Proteintech, USA	1:1000
Myc, HRP	R951-25-HRP	Invitrogen, Thermo Fisher Scientific, USA	1:3000
14-3-3ε	11648-2-AP	Proteintech, USA	1:10,000
α-actinin 4	19096-1-AP	Proteintech, USA	1:10,000
Calnexin	66903-1-Ig	Proteintech, USA	1:1000
GAPDH	60004-1-Ig	Proteintech, USA	1:10,000
CHOP	15204-1-AP	Proteintech, USA	1:1000
GRP78	11587-1-AP	Proteintech, USA	1:1000
HA, HRP	26183-HRP	Invitrogen, Thermo Fisher Scientific, USA	1:3000
p-AKT	10176-2-AP	Proteintech, USA	1:1000
AKT	10176-2-AP	Proteintech, USA	1:1000
LC3B	L7543	MilliporeSigma, USA	1:2000
p-mTOR-S2448	AP0094	ABclonal, USA	1:2000
mTOR	2972	Cell Signaling Technology, USA	1:1000
CD9	IR300-981	iREAL Biotechnology, Taiwan	1:500
CD63	IR301-983	iREAL Biotechnology, Taiwan	1:500
Annexin A1	55018-1-AP	Proteintech, USA	1:2000
Albumin	16475-1-AP	Proteintech, USA	1:20,000

The membranes were then incubated with secondary horseradish peroxidase (HRP)-conjugated goat-anti-mouse or goat-anti-rabbit IgG (PerkinElmer, at 1:10,000 dilution) at room temperature for 1 h and washed with 1 × Tris-buffered saline containing 0.05% Tween-20 (TBS-T) between steps. Luminescence signals were developed with Western Lightning^®^ ECL-Pro (PerkinElmer), and images were acquired using Hansor Luminescence Image System (Hansor Polymer Technology Corp., Taiwan) with TSGel software (version 3.5). Quantification of protein bands was conducted using ImageJ (version 1.50i).

### Reagents and recombinant proteins

Tunicamycin (Cat. No. NC1771734) was purchased from Cayman Chemical (Ann Arbor, Michigan, USA). Rapamycin (Cat. No. 51031-RAP-25) and Chloroquine (Cat. No. 581005-CLQ) were purchased from Enzo Life Sciences (Farmingdale, NY, USA). 3-Methyladenine (3-MA; Cat. No. HY-19312) was purchased from Med Chem Express (MCE, Monmouth Junction, NJ, USA). Recombinant canine AGR2 (rcAGR2) was prepared as previously described [14]. Recombinant 14-3-3ε (rc14-3-3ε; Cat. No. PKSH031395) was purchased from Elabscience (Houston, Texas, USA).

### Immunofluorescence staining and confocal microscopy

Cells were seeded onto coverslips placed in a 12-well plate and grown to 50–70% confluency, and subsequently cultured in serum-free media supplemented with or without 50 nM rapamycin for 16 h. Cells were fixed with 4% paraformaldehyde containing 2% sucrose in 1 × PBS at room temperature for 20 min and then permeabilized with 0.1% Triton X-100 for 3 min and blocked with BlockPRO™ (Visual protein, Taipei, Taiwan) for 30 min. Cells were incubated with an LC3B antibody (83506S, Cell Signaling Technology, Danvers, MA, USA) at 1:100 dilution, together with a 14-3-3 antibody (Cat. No. 11648-2-AP, Proteintech) at 1:400 dilution or an α-actinin 4 antibody (Cat. No. 19096-1-AP, Proteintech) at 1:300 dilution for 90 min at room temperature, followed by staining with secondary Alexa Fluor 488-conjugated goat-anti-mouse IgG and Alexa Fluor 694-conjugated goat-anti-rabbit IgG (Molecular Probe, Thermo Fisher Scientific), respectively, at 1:200 dilution for 45 min. Nuclei were co-stained with 4’,6-diamidino-2-phenylindole (DAPI, Invitrogen) at 0.1 µg/mL in 1 × PBS. Coverslips were washed with 1 × PBS between steps and finally mounted with Fluoro-Gel (Electron Microscopy Science, USA) on slides. Images were acquired using the Zeiss LSM780 confocal laser scanning microscope (Jena, Germany) with a 63 × oil-immersion objective.

### Quantification of intracellular puncta numbers and colocalization coefficients

The number of LC3B puncta, α-actinin 4-positive LC3B puncta, or 14-3-3ε-positive LC3B puncta, was quantified using ImageJ. Initially, confocal microscopy-acquired images were filtered with the Difference of Gaussian (DoG) filter to enhance the subcellular puncta structure. To quantify the LC3B puncta per cell, individual cells in each image were selected as the regions of interest (ROIs) and subsequently analyzed using the default “Analyze Particles” plugin to count the number of LC3B puncta within each cell.

For quantifying α-actinin 4-positive LC3B puncta or 14-3-3ε-positive LC3B puncta, the default “Colocalization Threshold” plugin was employed to generate overlay images of LC3B puncta and ACTN puncta, or LC3B puncta and 14-3-3ε puncta, prefiltered with the DoG filter, highlighting the overlapping regions. Subsequently, the same ROIs used for quantifying the LC3B puncta per cell in the previous steps were applied to the overlay images, and the “Analyze Particles” plugin was used to count the number of colocalized puncta within individual cells.

The colocalization coefficient was calculated using ZEN (black edition) software from Carl Zeiss AG in Oberkochen, Baden-Württemberg, Germany. To identify LC3B puncta, α-actinin 4 puncta, or 14-3-3ε puncta, the background subtraction thresholds were established based on the fluorescence intensities of the objects. Subsequently, colocalization coefficients between isolated puncta per cell in the overlay images were determined by measuring the overlapping regions between α-actinin 4 puncta and LC3B puncta or between 14-3-3ε puncta and LC3B puncta.

### Isolation of extracellular vesicles

CMT-U27e, Ctrl-S3, KO-S10, and KO-S4 were grown in RPMI supplemented with 2% EVs-depleted FBS for 50 h at 37 °C in a humidified incubator with 5% CO_2_ supply. For individual cell clones, conditioned media (CM) were collected from cells grown to 80–90% confluency in three 10-cm culture dishes and centrifuged at 300 × *g* for 10 min and subsequently at 3000 × *g* for 10 min at room temperature to remove detached cells and cell debris, respectively. The resulting CM was subjected to the isolation of extracellular vesicles using both differential ultracentrifugation (dUC) and size exclusion chromatography (SEC). For dUC, CM was first centrifuged at 16,500 × *g* at 4 °C for 30 min using the SW 28 Ti Swinging-Bucket Aluminum Rotor (Beckman Life Sciences, Indianapolis, IN, USA). The large EV pellet was washed with 0.22-µm-filtrated DPBS (Gibco, Thermo Fisher Scientific), followed by second centrifugation at 16,500 × *g* at 4 °C for 1 h to remove non-EV contaminants. The remaining supernatants were further centrifuged at 80,000 × *g* at 4 °C for 2 h, and the small EV pellet was washed with 0.22-µm-filtrated DPBS, followed by a second centrifugation at 80,000 × *g* at 4 °C for 2 h to remove non-EV contaminants. The washed large and small EVs were resuspended in 120 µL of 0.22-µm-filtrated DPBS and stored at − 80 °C before further analysis. The remained EV-depleted supernatants were used for a transwell migration assay or immunoblotting.

For SEC, the qEVoriginal/70 nm Gen 2 Column (Izon Science Ltd., New Zealand) was used to isolate EVs. Cell debris-removed CM was first concentrated with Vivaspin^®^ 20 (100 kD cutoff; GE Healthcare) and then added to the qEV column. Once the CM sample was filled in the column, 500 µL of 0.22-µm-filtrated and degassed DPBS was added to the top of the column. Flow-through fractions were immediately collected as follows: void buffer fractions 1 to 6 and sample fractions 1 to 14 (500 µL each), by the manufacturer’s instruction. The collected fractions were stored at − 80 °C until further use. For a transwell migration assay, 500 µL of 2% FBS-containing RPMI supplemented with 100 µL of the EV fraction (Fraction 1) or DPBS was placed in the bottom well as the attractant.

### Nanoparticles tracking analysis (NTA)

NTA was employed to determine the absolute size distribution and concentration of extracellular vesicles (EVs). The analysis was conducted using the NanoSight NS300 instrument (NanoSight, Minton Park, UK) in conjunction with NanoSight NTA software (version 3.4; NanoSight) for both data acquisition and analysis. Particles were automatically tracked and sized based on their Brownian motion and diffusion coefficient. Filtered PBS served as the control and blank samples. The NTA measurement conditions were standardized as follows: temperature maintained at 24.0 ± 0.5 °C, viscosity at 0.99 ± 0.01 cP, frames per second set at 25, and a measurement time of 60 s. The detection threshold remained consistent across all samples. Each sample underwent five recordings to ensure accuracy and reliability of the results.

### Transmission electron microscopy (TEM)

TEM was utilized to investigate the morphology of EVs. Initially, isolated EVs were resuspended in 4% paraformaldehyde (50–100 μL), and 10 μL aliquots were deposited onto Formvar/carbon-coated EM grids. The grids were then covered, and membrane adsorption was carried out for 20 min in a dry environment. Subsequently, the grids (with the membrane side down) were transferred to drops of PBS (100 μL) using clean forceps for washing, followed by retransfer to a 50 μL drop of 1% glutaraldehyde for 5 min. Afterwards, the grid underwent eight washes with distilled water, each lasting 2 min. Following this, contrast staining was achieved by immersing the grid in a 50 μL drop of uranyl acetate solution for 5 min. Finally, the grid was embedded in 50 μL of methyl cellulose-UA for 10 min on ice. Upon removal of the grid using stainless steel loops, excess fluid was blotted, and the grid was air-dried. The prepared grid was then examined under an electron microscope (JEM 1230, JEOL Ltd., Tokyo, Japan) operating at 80 kV.

### Specimen collection

Pre-surgical serum samples were collected from 17 dogs afflicted with CMT who underwent mastectomy at the Veterinary Medical Teaching Hospital (VMTH), National Chung Hsing University (NCHU), between 2017 and 2019. Additionally, serum samples were collected from 15 privately owned, age-matched healthy female dogs for comparison purposes. Blood samples were drawn into serum separating tubes (SSTs) and allowed to clot at room temperature for 30 min before being centrifuged at 2500 × *g*, 4 °C, for 15 min. The resulting sera were supplemented with a protease inhibitor cocktail (VWP Life Science, Avantor, Radnor Township, PA, USA), divided into 50 μL aliquots, and stored at − 80 °C until utilization.

Paired samples of CMT tissues and non-involved normal mammary gland tissues were collected from 9 out of the 17 CMT patients who underwent simple or bilateral mastectomy at the VMTH, NCHU. These tissues subsequently utilized for collecting the tissue-immersed PBS, mimicking interstitial fluids. All procedures were conducted in compliance with relevant guidelines and regulations approved by the Institutional Animal Care and Use Committee (IACUC) of NCHU (IACUC Number: 109–002). Diagnosis of CMTs was confirmed through radiography and histopathological examination of surgically excised tissues. Classification, histopathological grade, and clinical stage of CMTs were determined on the basis of the modified WHO-TNM system [3].

### Preparation of the tissue-immersed PBS

Paired samples of CMT tissues and non-involved normal mammary gland tissues (5 × 5 × 5 mm^3^) were harvested during surgical procedures. Upon weighting, the tissues were promptly processed as previously described [67]. To minimize blood contamination, the tissues were thoroughly washed with ice-cold PBS and then dissected into 1–3 mm^3^ fragments using scalpels. The cut tissues were placed into 1.5-mL microcentrifuge tubes and subjected to further washed by ice-cold PBS until the supernatant was clear. Subsequently, the cut tissues were incubated with 600 μL of PBS for 1 h in a humidified incubator at 37 °C containing 5% CO_2_. Following centrifuging at 8000 × *g* for 15 min at 4 °C, the resulting supernatants were promptly treated with a protease inhibitor mixture (2 μL/mL; VWP Life Science) and stored at − 80 °C for subsequent analysis.

### Statistical analysis

The statistical analysis was conducted by using GraphPad Prism V8.4 software (GraphPad Inc., San Diego, CA, USA). The two-tailed unpaired t-test was applied to evaluate experimental differences between groups, and the Mann–Whitney U test was utilized to assess differences between clinical samples. *p* < 0.05 was considered statistically significant.

## Results

### Enhanced AGR2 expression promotes CMT cell chemotaxis through modulating extracellular milieu

**Fig. 1:**
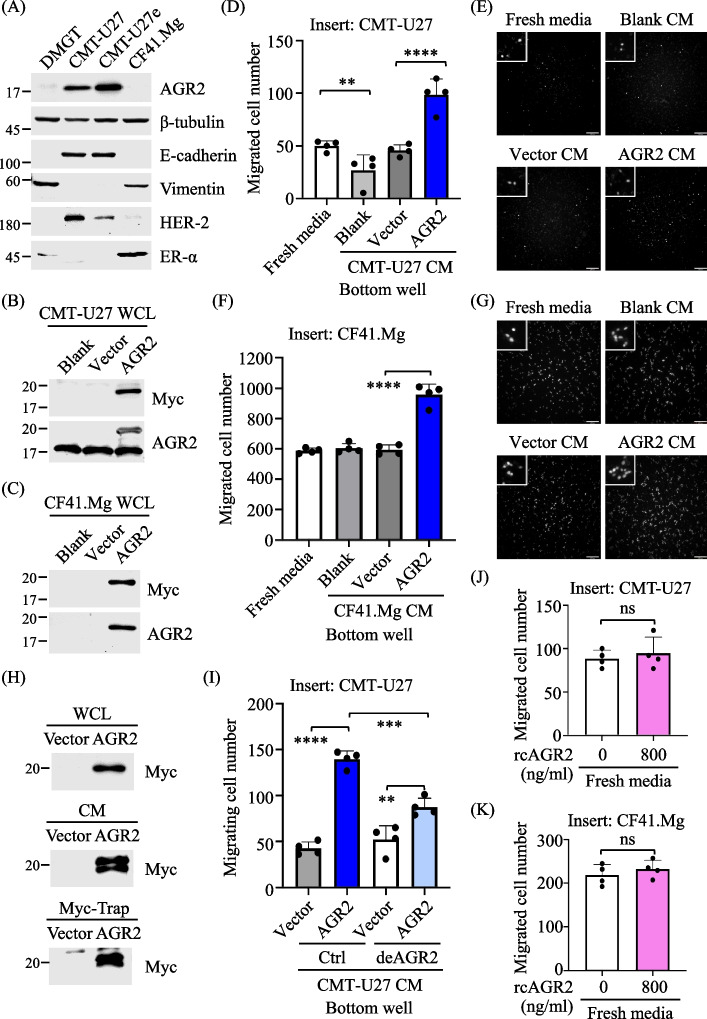
Ectopic expression of AGR2 modulated extracellular milieu of CMT cells, promoting CMT cell chemotaxis. **A** Characterization of CMT cell lines. Expression of AGR2, E-cadherin, vimentin, HER-2, or ER-⍺ in individual cell lines was analyzed by immunoblotting with specific antibodies. CMT-U27 (**B**) and CF41.Mg (**C**) were transfected with pcDNA3.1-myc.His-AGR2 or the mock vector and grown in 2% FBS-containing RPMI and DMEM, respectively, for 24 h. Whole-cell lysates (WCL) of the transfectants were analyzed by immunoblotting to confirm the expression of Myc-tagged AGR2. Conditioned media (CM) of the transfectants were collected and placed in the bottom well for a transwell migration assay, in which the responding cells were seeded in the top insert and stained with Hoechst during a 16-h incubation. Cells in the insert were fixed for image acquisition using an epifluorescence microscope with a 10 × objective. **D, F** The number of migrated cells was counted and presented as the mean + SD of three independent experiments. **E, G** Representative images of migrated cells per field were shown. **H** Myc-Trap-based precipitation conducted the depletion of AGR2 in the CM of AGR2-expressing CMT-U27. Expression of AGR2 in WCL or CM and Myc-Trap-precipitated AGR2 were verified by immunoblotting. **I** As described above, the AGR2-depleted CM (denoted deAGR2) or the untreated control was placed in the bottom well for a transwell migration assay. **J, K** Fresh 2% FBS-containing media supplemented with or without rcAGR2 (800 ng/mL) were placed in the bottom well for a transwell migration assay. For **C, F, I–K**, statistical significance was determined by a two-tailed unpaired *t*-test. *p < 0.05; **p < 0.01; ****p < 0.0001

To investigate the impact of enhanced AGR2 expression on the extracellular milieu of CMT cells, we conducted immunoblotting to assess AGR2 expression status in CMT cell lines: CMT-U27, CMT-U27e, CF41.Mg, and DMGT. CMT-U27 and CMT-U27e displayed an epithelial morphology, characterized by E-cadherin positivity and Vimentin negativity, and expressed the human epidermal growth factor receptor 2 (HER-2) but not the estrogen receptor α (ER-α), as depicted in Fig. 1A. In contrast, CF41.Mg and DMGT exhibited a mesenchymal phenotype, lacking E-cadherin and expressing Vimentin, and expressed ER-α but little to no HER-2. Endogenous AGR2 was detected in CMT-U27 and found to be expressed at elevated levels in the subline CMT-U27e, whereas AGR2 expression was barely detectable in CF41.Mg and DMGT.

Subsequently, we transfected CMT-U27, CF41.Mg, and DMGT with AGR2-expressing plasmids to enhance AGR2 expression. Confirmation of ectopic Myc-tagged AGR2 expression in the transfectants was achieved through immunoblotting (Fig. 1B, C, and Fig. S1A, respectively). To evaluate the functional consequences of altered AGR2 expression, we collected spent conditioned media (CM) of the transfectants and employed a transwell migration assay. Figure 1D and E illustrate that CM from AGR2-expressing CMT-U27 significantly enhanced the chemotaxis of CMT cells compared with the CM from vector-expressing or non-transfected control cells. Likewise, CM from AGR2-expressing CF41.Mg and DMGT also exhibited chemotaxis-promoting activity (Fig. 1F, G, and supplementary Fig. S1B, respectively).

To verify the impact of AGR2 on the extracellular milieu of human breast cancer cells, we transfected both invasive MDA-MB-231 and non-invasive MCF7 cells with an expression vector encoding human AGR2. Subsequently, we evaluated the chemotaxis-promoting effect of the resulting CM. MDA-MB-231 expressed undetectable levels of endogenous AGR2, while MCF7 exhibited high levels of endogenous AGR2 expression. The expression of ectopic AGR2 in both cell lines was confirmed by immunoblotting, as shown in Fig. S1C and E, respectively. Furthermore, CM collected from both MDA-MB-231 and MCF7 cells expressing ectopic AGR2 demonstrated chemotaxis-promoting activity compared with CM collected from the vector-expressing or blank control (supplementary Fig. S1D and F, respectively). These findings suggest that the phenomena observed in CMT cells also occur in human breast cancer cells.

To ascertain whether the chemotaxis effect could be attributed to secreted AGR2, we conducted Myc-Trap-based immunoprecipitation (IP) to deplete extracellular AGR2 from the CM of AGR2-expressing CMT-U27. Immunoblotting analysis confirmed the presence of AGR2 in CM trapped by Myc-Trap (Fig. 1H). We then applied this AGR2-depleted CM (deAGR2) to a transwell migration assay. As depicted in Fig. 1I, AGR2-depleted CM still retained the ability to attract CMT cells, albeit to a lesser extent than the complete CM. Moreover, the addition of recombinant AGR2 (rcAGR2) to fresh CM showed no chemotaxis effect (Fig. 1J and K). These findings suggest that enhanced AGR2 expression modulates components in the extracellular milieu, contributing to CMT cell chemotaxis.

### Proteomics analysis unveils the AGR2-affected secretome in CMT cells

**Fig. 2:**
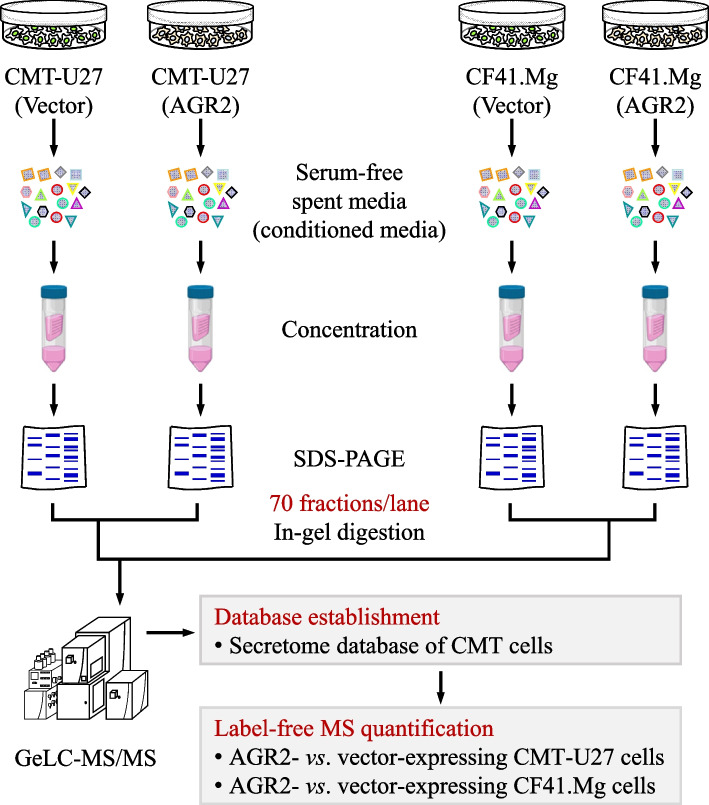
Identification of AGR2-affected secretome of CMT cells. Schematic diagram for identification of AGR2-affected secretome. Serum-free conditioned media (CM) were prepared from CMT cells (CMT-U27 or CF41.Mg) which had been transfected with pcDNA3.1-myc.His-AGR2 or the mock vector and were grown for another 24 h to 90% confluency. The CM samples were concentrated and resolved by SDS-PAGE and subsequently stained with 0.5% Coomassie Brilliant Blue G-250. Individual protein lanes were cut into gel slices and applied to in-gel digestion, and the resulting peptides were analyzed using a GeLC-MS/MS-based proteomics pipeline. The abundance of identified proteins was determined by label-free MS quantification

**Fig. 3:**
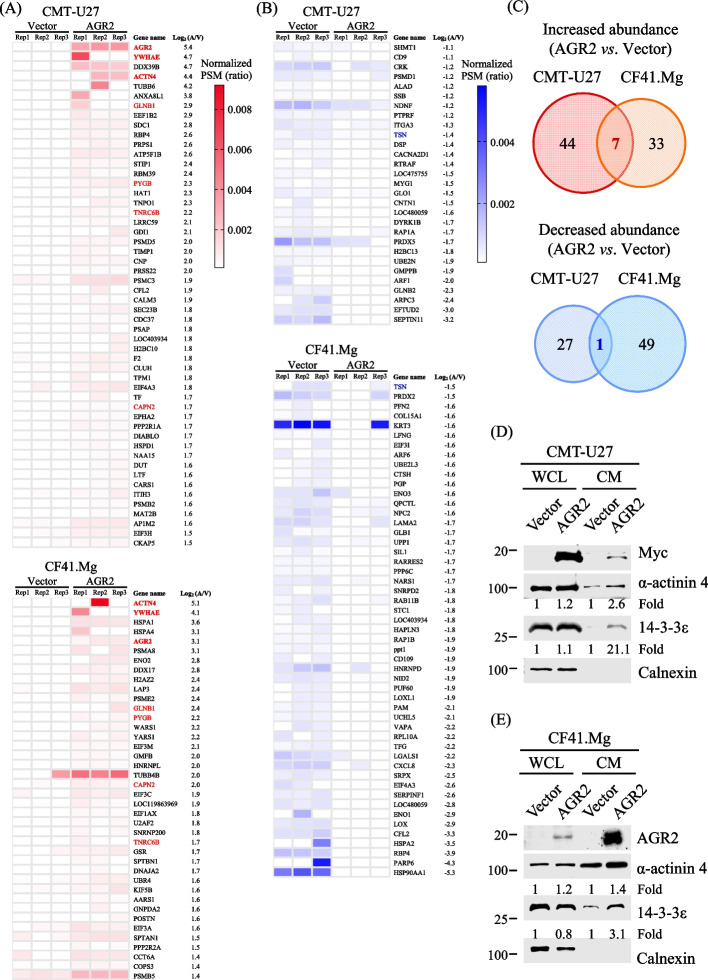
Differentially present proteins in the CM of AGR2-expressing CMT cells. **A, B** Heatmap of the proteins identified as AGR2-increased (**A**) and AGR2-decreased (**B**) in the CM of CMT-U27 or CF41.Mg. Each CM sample was triply analyzed (Rep1, Rep2, Rep3). Protein abundance was determined by normalizing PSMs of specific proteins to total PSMs in replicates. Cumulative normalized PSM ratios of AGR2-expressing CM were divided by vector-expressing CM. This ratio underwent logarithmic transformation (Log2) as Log2 (A/V). Proteins with Log2 (A/V) above mean + 1.5 SD were classified as AGR2-increased; below mean-1.5 SD were designated as AGR2-decreased. AGR2-increased and AGR2-decreased proteins which are in common in both CMT-U27 CM and CF41.Mg CM are highlighted in red (**A**) and blue (**B**), respectively, and are shown in the overlap of Venn diagrams (**C**). **D, E** Verification of increased 14-3-3ε and α-actinin 4 levels in the CM of AGR2-expressing CMT cells. CMT-U27 (**D**) or CF41.Mg (**E**) cells transfected with the AGR2-expressing or the mock vector were grown in serum-free CM for 24 h, and the CM samples were collected and concentrated. The concentrated CM and whole-cell lysate (WCL) were analyzed by immunoblotting with indicated specific antibodies. Levels of 14-3-3ε and α-actinin 4 in the AGR2-expressing group were presented as fold changes to that in the vector group. Calnexin was used as a negative control in the CM

To decipher the secretome influenced by AGR2 and its association with chemotaxis, we utilized gel-enhanced liquid chromatography-tandem mass spectrometry (GeLC-MS/MS) to analyze the CM from AGR2-expressing CMT-U27 and CF41.Mg cells, which were effectively transfected, in comparison to their vector-expressing controls (Fig. 2). In total, 798 proteins in CMT-U27 and 788 proteins in CF41.Mg were successfully identified and quantified (supplementary Table S1 and  Table S2, respectively). Among these proteins, 51 and 40 CM proteins were identified as AGR2-increased, while 28 and 50 CM proteins were designated as AGR2-decreased in CMT-U27 and CF41.Mg, respectively (Fig. 3A–C). Intriguingly, seven CM proteins exhibited increased abundance in both CMT-U27 and CF41.Mg cells (Fig. 3A and C), including AGR2, 14-3-3ε (gene: YWHAE), and α-actinin-4 (gene: ACTN4), all of which demonstrated prominent increases in magnitude (Fig. 3A). Furthermore, translin (TSN) was identified as common AGR2-decreased in both CMT-U27 and CF41.Mg (Fig. 3B and C).

### AGR2 expression augments the release of 14-3-3ε and α-actinin-4 in the CM

Given that 14-3-3ε and α-actinin 4 were among the top-ranked CM proteins increased by AGR2 and were shared by both CMT cell lines, we conducted experiments to validate whether AGR2 expression led to elevated levels of 14-3-3ε and α-actinin 4 in the CM of CMT cells. As depicted in Fig. 3D and E, ectopic expression of AGR2 in CMT-U27 and CF41.Mg, respectively, resulted in increased release of 14-3-3ε and α-actinin 4 into the CM. Notably, ectopic expression of AGR2 had minimal impact on the protein expression levels of 14-3-3ε and α-actinin 4 in cell lysates. Furthermore, we confirmed that ectopic expression of AGR2 in MCF7 and MDA-MB-231 also enhanced the release of 14-3-3ε and α-actinin 4, as shown in Fig. S2A and B, respectively.

In contrast, ectopic AGR2 expression did not induce the release of an ER protein, Calnexin, in the same experimental context (Fig. 3D and E), implying that the observed events were not indicative of a general ER disorganization or rupture caused by ectopic AGR2 expression.

### Genetic depletion of AGR2 leads to the diminished release of 14-3-3ε and α-actinin 4 in the CM, alongside impaired chemotaxis activity

**Fig. 4:**
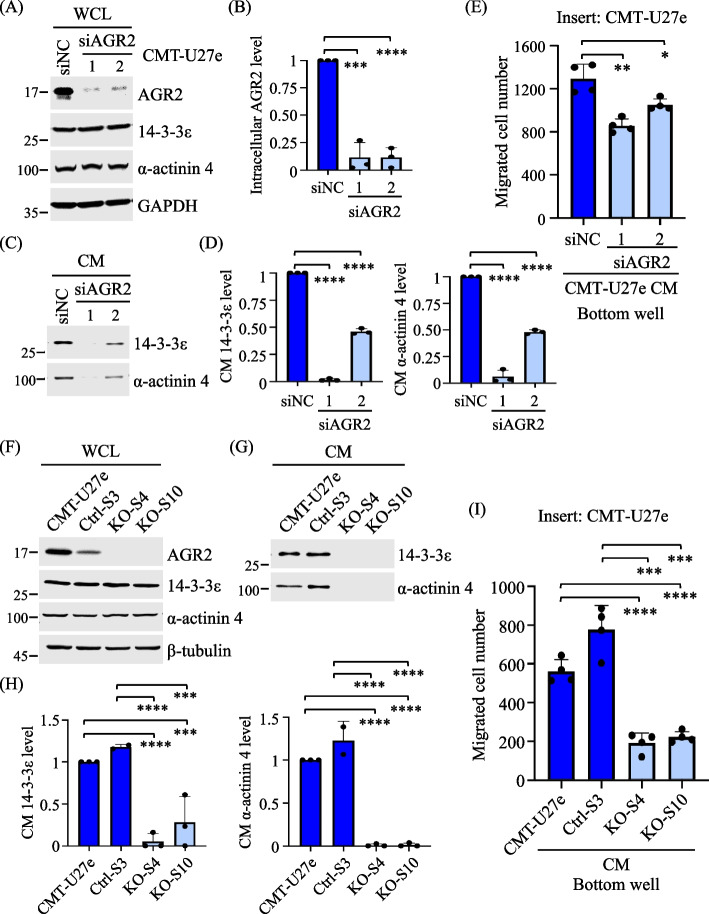
Genetic depletion of AGR2 impaired 14-3-3ε and α-actinin 4 release in the CM of CMT cells. **A** CMT-U27e transfected with *AGR2*-targeting siRNA (siAGR2 1 or 2) or negative control siRNA (siNC) was grown in 1% FBS-containing RPMI for 30 h. WCL and CM samples were collected and subjected to immunoblotting analysis. Protein bands were quantified and normalized to that of GAPDH, which was used as an internal control. AGR2 expression levels in siAGR2 transfectants were presented as a ratio to that in siNC transfectants. Data were presented as the mean + SD of three independent experiments, shown in **B**. **C** CM samples collected from (**A**) were TCA-precipitated, and 14-3-3ε and α-actinin 4 levels were analyzed by immunoblotting and quantified as described above. Data are presented as the mean + SD of three independent experiments, shown in **D**. **E** CM collected from (**A**) was applied to a transwell migration. Results are presented as the mean + SD of three independent experiments. **F, G** CMT-U27e, a control cell clone Ctrl-S3, and two AGR2-KO clones, KO-S4 and KO-S10, were grown in 1% FBS-containing RPMI for 24 h. WCL and CM samples were collected and analyzed by immunoblotting, as shown in **F** and **G**, respectively. 14-3-3ε and α-actinin 4 levels in individual CM samples are presented as a ratio to that in CMT-U27e, as shown in **H**. **I** CM collected from individual cell clones was applied to a transwell migration assay. Results are presented as the mean + SD of three independent experiments *p < 0.05; **p < 0.01; ***p < 0.001; ****p < 0.0001 (two-tailed unpaired *t*-test)

We proceeded to investigate whether depletion of AGR2 would impair the release of 14-3-3ε and α-actinin 4 into the CM of CMT cells and influence the chemotaxis activity of the CM. Initially, we employed RNA interference (RNAi) to silence AGR2 expression in CMT-U27e by transfecting with siRNA duplexes targeting the canine AGR2-encoding gene. Immunoblotting analysis confirmed the efficacy of AGR2 silencing, with an efficiency exceeding 75% (Fig. 4A and B). Importantly, AGR2 knockdown significantly reduced the extracellular levels of 14-3-3ε and α-actinin 4 (Fig. 4C and D), while their expression levels remained unaffected (Fig. 4A). Subsequently, the transwell migration assay revealed that the CM from AGR2-depleted CMT-U27e exhibited diminished chemotaxis activity compared with the control (Fig. 4E).

To further evaluate the impact of AGR2 on the release of 14-3-3ε and α-actinin 4, we generated AGR2-knockout (KO) CMT-U27 cell clones using a CRISPR/Cas9-based methodology. Immunoblotting analysis confirmed the complete absence of AGR2 expression in selected AGR2-KO clones, namely KO-S10 and KO-S4 (Fig. 4F). Significantly, the levels of 14-3-3ε and α-actinin 4 in the CM of AGR2-KO cells were markedly reduced compared with those in the control CMT-U27e or Ctrl-S3 (Fig. 4G and H). Furthermore, the CM from AGR2-KO cells displayed substantially diminished chemotaxis activity in comparison to the control CM (Fig. 4I). These results collectively confirm that AGR2 effectively regulates the release of 14-3-3ε and α-actinin 4 into the CM, which in turn influences CMT cell chemotaxis.

### Silencing the expression of 14-3-3ε or α-actinin 4 impairs the chemotaxis activity of AGR2-modulated CM

**Fig. 5:**
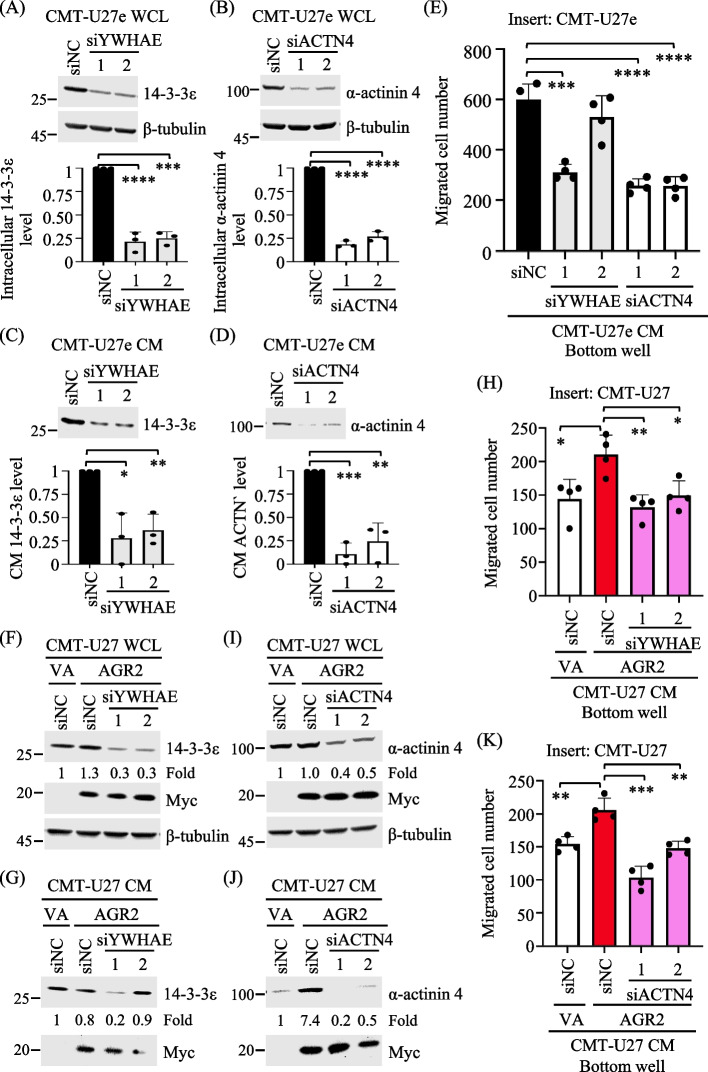
Knockdown of 14-3-3ε or α-actinin 4 diminished the chemotaxis conferred by AGR2-modulated CM. **A**, **B** CMT-U27e transfected with 14-3-3ε-targeting siRNA, siYWHAE (**A**), α-actinin 4-targeting siRNA, siACTN4 (**B**), or negative control siRNA (siNC) were grown in 1% FBS-containing RPMI for 30 h. WCL samples were harvested and analyzed by immunoblotting. 14-3-3ε and α-actinin 4 levels were normalized to β-tubulin levels in individual samples, and the resulting values in siYWHAE- or siACTN4-transfectants were presented as a ratio to that in siNC. **C**, **D** 14-3-3ε and α-actinin 4 levels in the CM samples collected respectively from (**A**) and (**B**) were analyzed by immunoblotting and processed as above. **E** CM samples collected from (**B, D**) were applied to a transwell migration assay. **F, G, I, J** CMT-U27e cells were first transfected with the indicated siRNA (75 nM) and subsequently transfected with pcDNA3.1-myc.His-AGR2 or the mock control 6 h later. After 8 h incubation, the culture media were replaced with 1% FBS-containing RPMI, and cells were grown for another 20 h until WCL and CM were collected. Levels of indicated proteins in individual WCL (**F, I**) and CM (**G, J**) were analyzed and quantified as described above. **H, K** CM samples collected from (**I, J**) were applied to a transwell migration assay. All quantitation data shown are the mean + SD of three independent experiments. *p < 0.05; **p < 0.01; ***p < 0.001; ****p < 0.0001 (two-tailed unpaired *t*-test)

To ascertain whether reducing the release of 14-3-3ε and α-actinin 4 would diminish the chemotaxis activity of CM, we employed siRNA-mediated RNAi to silence the expression of 14-3-3ε or α-actinin 4 in CMT-U27e. Immunoblotting analysis confirmed the reduced expression of 14-3-3ε and α-actinin 4 in the corresponding siRNA transfectants (Fig. 5A and B), accompanied by the reduced levels of 14-3-3ε and α-actinin 4 in the CM (Fig. 5C and D). Subsequently, the transwell migration assay revealed that the reduced levels of CM 14-3-3ε or α-actinin 4 resulted in the impaired chemotaxis activity of CM (Fig. 5E).

To further elucidate if 14-3-3ε and α-actinin 4 mediate the chemotaxis activity of AGR2-modulated CM, we silenced the expression of 14-3-3ε or α-actinin 4 in CMT-U27 which co-expressed ectopic AGR2. Immunoblotting analysis verified the expression of Myc-tagged AGR2 and the attenuated expression of 14-3-3ε and α-actinin 4 in the corresponding transfectants, as shown in Fig. 5F and I. The levels of 14-3-3ε and α-actinin 4 were also diminished in the CM of AGR2-expressing CMT-U27 co-transfected with specific siRNAs, as compared with the control (Fig. 5G and J). Furthermore, the diminished levels of 14-3-3ε and α-actinin 4 in the CM compromised the chemotaxis activity of AGR2-modulated CM (Fig. 5H and K). The cumulative findings demonstrate that AGR2 effectively regulates the release of 14-3-3ε and α-actinin 4, thereby enhancing CMT cell chemotaxis.

### AGR2 facilitates the release of 14-3-3ε and α-actinin 4, primarily in non-vesicular form and minorly in extracellular vesicles

Since 14-3-3ε and α-actinin 4 lack a signal peptide required for the conventional ER-to-Golgi secretion, we hypothesized that their release likely occurs through unconventional protein secretion (UPS), such as the extracellular vesicle (EV)-mediated secretion [68, 69]. To investigate whether AGR2 regulates the release of 14-3-3ε and α-actinin 4 via EVs, we initially employed differential ultracentrifugation (dUC; supplementary Fig. S3A) to isolate large EVs (lEVs) and small EVs (sEVs) derived from CMT-U27e, Ctrl-S3, KO-S10 or KO-S4. We subjected lEVs and sEVs derived from Ctrl-S3 cells to nanoparticle tracking analysis (NTA) and transmission electron microscopy (TEM) to confirm the efficiency of EV isolation. NTA results showed that the mean diameters of lEVs and sEVs were 141.7 ± 2.2 nm and 112.3 ± 1.9 nm, respectively (Fig. S3B). TEM images depicted the morphology of the isolated lEV and sEV (Fig. S3C).

Furthermore, immunoblotting results indicated that the levels of 14-3-3ε and α-actinin 4 in the CM were not significantly affected by EV depletion across all the cell lines (Fig. S3D). However, a fraction of 14-3-3ε and α-actinin 4 was detected in lEVs (mainly) and sEVs (minority) derived from CMT-U27e or Ctrl-S3. Notably, the levels of 14-3-3ε and α-actinin 4 in the EVs were significantly reduced in AGR2-KO cells (supplementary Fig. S3E).

To confirm the impact of AGR2 on the delivery of 14-3-3ε and α-actinin 4 via EVs, we conducted size exclusion chromatography (SEC) to isolate the EVs derived from control or AGR2-KO cells (supplementary Fig. S4A). Immunoblotting analysis revealed that 14-3-3ε and α-actinin 4 were present in the CD9-positive EV fractions, particularly Fraction 1, but mostly were detected in the protein fractions (Fractions 9–14) of CMT-U27e (supplementary Fig. S4B), consistent with the previous dUC results. NTA results showed that the mean diameter of EVs was 115.4 ± 2.7 nm, ranging from 70 to 322 nm (Fig. S4C). The morphology of EVs with varied sizes was confirmed using TEM (supplementary Fig. S4D).

Furthermore, we compared the levels of 14-3-3ε and α-actinin 4 in the EV fractions from control cells with those from AGR2-KO cells. The results confirmed reduced levels of 14-3-3ε and α-actinin 4 in the EV fractions from AGR2-KO cells (supplementary Fig. S4E).

To assess the contribution of EV to the CM-driven chemotaxis, we utilized both the complete CM and EV-depleted CM in a transwell migration assay. The results revealed that EV depletion had minimal effect on the chemotaxis activity of CM in all the cell lines (supplementary Fig. S3F), indicating that 14-3-3ε and α-actinin 4 are primarily released as vesicle-free proteins that enhance chemotaxis.

To evaluate the chemotaxis activity of EVs, we subjected EV fractions (Fraction 1) from CMT-U27e, Ctrl-S3, or AGR2-KO cells to a transwell migration assay. Supplementary Fig. S4F illustrated that the EVs derived from AGR2-KO cells displayed diminished chemotaxis activity compared with EVs from control cells, suggesting that the EV-delivered 14-3-3ε and α-actinin 4 might contribute to this effect.

### AGR2 controls the release of 14-3-3ε and α-actinin 4 upon ER stress and autophagy induction

**Fig. 6:**
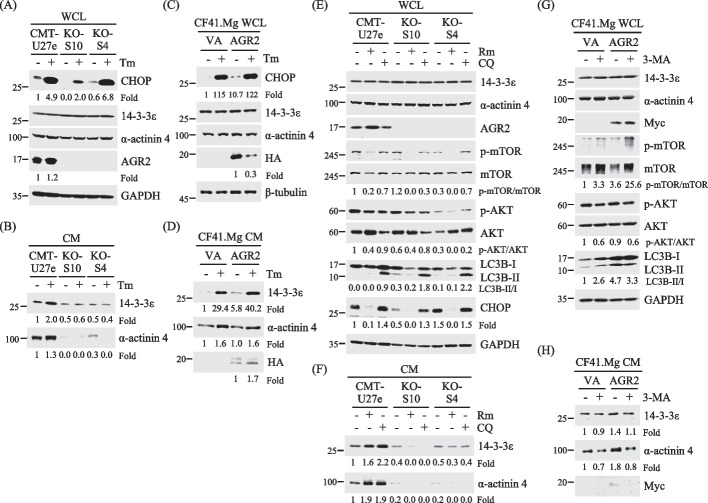
AGR2 modulated the release of 14-3-3ε and α-actinin 4 upon ER stress and autophagy induction. **A**, **B** CMT-U27e or AGR2-KO clones (KO-S10 and KO-S4) were cultured in 1% FBS-containing RMPI and treated with or without 50 nM tunicamycin (denoted Tm) for 14 h. Cell lysate (WCL) samples were collected and subjected to immunoblotting analysis with antibodies specific to indicated proteins. The CM samples were further TCA-precipitated to analyze 14-3-3ε or α-actinin 4 levels with immunoblotting (**B**). **C**, **D** CF41.Mg transfected with pcDNA3.1-HA-AGR2 or the mock control were subsequently cultured in 1% FBS-containing DMED for 14 h with or without addition of 50 nM Tm. Levels of the indicated proteins in WCL (**C**) and CM (**D**) were analyzed by immunoblotting. **E**, **F** CMT-U27e, KO-S10, or KO-S4 were cultured in serum-free RPMI and treated with 300 nM rapamycin (denoted Rm) or 40 µM chloroquine (denoted CQ) for 16 h. Levels of the indicated proteins in WCL (**E**) and CM (**F**) were analyzed by immunoblotting. **G**, **H** CF41.Mg transfected with pcDNA3.1-myc.His-AGR2 or the mock control was cultured in serum-free DMEM supplemented with or without 3-MA (2 mM) for 16 h. Levels of the indicated proteins in WCL (**G**) and CM (**H**) were analyzed by immunoblotting. The present results were representative data from three independent experiments

Next, we investigated the role of AGR2 in regulating the release of 14-3-3ε and α-actinin 4 during specific cancer progression-related stress conditions, including the unfolded protein response (UPR)-induced ER stress and autophagy [70–72]. To accomplish this, we exposed CMT-U27e and AGR2-KO cells (KO-S10 and KO-S4) to 50 nM tunicamycin treatment in media containing 1% FBS and then collected cell lysates and CM samples for immunoblotting analysis. Figure 6A illustrates the upregulated expression of an ER stress indicator, C/EBP homologous protein (CHOP), in response to tunicamycin-induced ER stress in all the tested cells, while basal CHOP levels in AGR2-KO cells declined. Furthermore, tunicamycin treatment led to an increased release of α-actinin 4 and 14-3-3ε from CMT-U27e, but this effect was impaired in AGR2-KO cells (Fig. 6B; replicate results seen in supplementary Fig. S5A and S5B).

Conversely, tunicamycin treatment induced CHOP expression in CF41.Mg cells and ectopic AGR2 expression intensified this stress response (Fig. 6C), accompanied by the enhanced release of 14-3-3ε and α-actinin 4 (Fig. 6D; replicate results seen in supplementary Fig. S5C and S5D). Similarly, these phenomena were also observed in MDA-MB-231 and DMGT cells (supplementary Fig. S2B).

Considering the involvement of autophagy in unconventional protein secretion [71, 73], we explored the role of autophagy in the AGR2-controlled release of 14-3-3ε and α-actinin 4. We cultured CMT-U27e and AGR2-KO cells in serum-free media supplemented with 300 nM rapamycin or 40 mM chloroquine, which inhibits autophagosome-lysosome fusion, to induce autophagy. Immunoblotting analysis revealed that rapamycin treatment led to the inhibition of phosphorylation of the mammalian target of rapamycin (p-mTOR), accompanied by a reduction of AKT phosphorylation and CHOP expression in all the analyzed cells (Fig. 6E). Additionally, chloroquine treatment resulted in the processing of microtubule-associated protein 1 light-chain 3B (LC3B)-I to LC3B-II in all the treated cells, indicating autophagy activation. Notably, both rapamycin and chloroquine treatments facilitated the release of 14-3-3ε and α-actinin 4 in CMT-U27e cells (Fig. 6F), and similar effects were observed in DMGT, MDA-MB-231, and MCF7 cells (supplementary Fig. S2C). However, this effect was significantly impaired in both AGR2-KO cells (Fig. 6F; replicate results seen in supplementary Fig. S6A and S6B).

To confirm the involvement of autophagy in the AGR2-controlled release of 14-3-3ε and α-actinin 4, we ectopically expressed AGR2 in CF41.Mg cells and subsequently treated the cells with 3-MA to inhibit autophagy. In Fig. 6G, it was evident that ectopic expression of AGR2 led to an increased level of p-mTOR, along with enhanced processing of LC3B-II to LC3B-I, compared to the vector-expressing control. In this scenario, 3-MA treatment resulted in an elevated level of p-mTOR, a decreased level of p-AKT, and a reduced LC3B-II-to-LC3B-I ratio in the AGR2-expressing cells. Furthermore, 3-MA reduced the AGR2-controlled release of 14-3-3ε and α-actinin 4 by 0.79-fold and 0.44-fold, respectively (Fig. 6H; replicate results seen in supplementary Fig. S6C and S6D). These findings reveal that the AGR2-controlled release of 14-3-3ε and α-actinin 4 is intricately linked to ER stress and autophagic processes.

### Knockout of AGR2 results in the accumulation of α-actinin 4 and reduced translocation of 14-3-3ε in the autophagosome

**Fig. 7:**
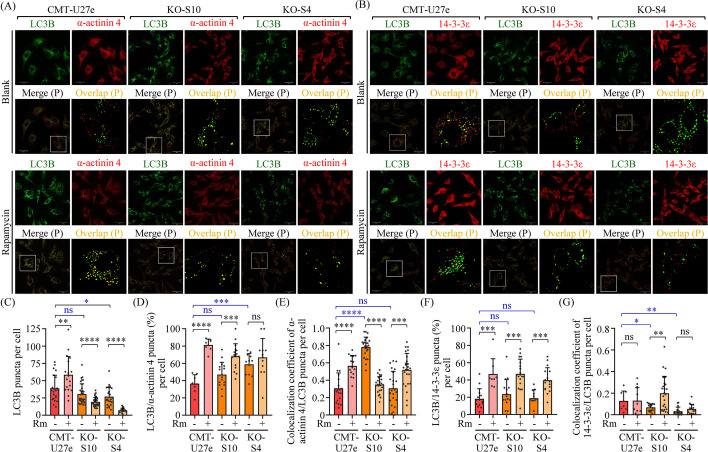
Depletion of AGR2 resulted in aberrant translocation of 14-3-3ε or α-actinin 4 to the autophagosome. CMT-U27e, KO-S10, and KO-S4 seeded on coverslips were cultured in serum-free RPMI for 16 h, with or without adding 50 nM rapamycin (Rm). The cells were subsequently fixed and subjected to immunofluorescence staining of α-actinin 4 (**A**) or 14-3-3ε (**B**), together with LC3B. The resulting images were acquired using confocal microscopy. Puncta exhibiting LC3B (i.e., autophagosomes), α-actinin 4, or 14-3-3ε were identified using a Difference of Gaussian processing filter and presented in overlay images, denoted Merge (P). Scale bar, 20 µm. Colocalization of α-actinin 4 puncta with LC3B puncta, or 14-3-3ε puncta with LC3B puncta, were shown as overlap regions (yellow) in the enlargement, denoted Overlap (P). The number of LC3B puncta per cell (**C**) and the percentage of the LC3B puncta exhibiting colocalization with α-actinin 4 (**D**) or with 14-3-3ε (**F**) across all the experimental groups were quantified. Colocalization coefficients were measured for the puncta showing α-actinin 4-LC3B colocalization (**E**) and those showing 14-3-3ε-LC3B colocalization (**G**) per cell. The data in panels (**C**) through (**G**) were presented as the mean + SD

To investigate the involvement of autophagic processes in the AGR2-controlled release of α-actinin 4 and 14-3-3ε, we examined the intracellular localization of α-actinin 4 or 14-3-3ε in conjunction with LC3B in CMT-U27e and two AGR2-KO cells under serum starvation or rapamycin treatment. The results revealed that LC3B-positive puncta (representing autophagosomes) were observed in all analyzed cells under serum starvation (Fig. 7A and B). Upon rapamycin treatment, the autophagosome numbers were increased in CMT-U27e cells; however, the autophagosome numbers decreased instead in both KO-S10 and KO-S4 cells (Fig. 7C). In this context, we examined whether α-actinin 4 or 14-3-3ε was translocated to the autophagosomes and if such events were affected in AGR2-KO cells. We used the percentage of autophagosomes containing α-actinin 4 or 14-3-3ε per cell, along with colocalization coefficients between α-actinin 4 and autophagosomes or between 14-3-3ε and autophagosomes, to evaluate the impact of AGR2 on the translocation of α-actinin 4 or 14-3-3ε to the autophagosomes. Under serum starvation, we observed that the percentage of α-actinin 4-containing autophagosomes was increased in KO-S4 cells compared to CMT-U27e cells (Fig. 7D). Although, in KO-S10 cells, the increase in the percentage of α-actinin 4-containing autophagosomes did not achieve a statistical significance (*p* = 0.08), the colocalization coefficients were significantly higher than those in CMT-U27e cells (Fig. 7E). Additionally, rapamycin treatment increased the percentage of α-actinin 4-containing autophagosomes and/or the colocalization coefficients in either CMT-U27e or AGR2-KO cells, indicating that the rapamycin-induced translocation of α-actinin 4 was unaffected. Together, these results suggest that AGR2 knockout may impair the autophagic flux and lead to the accumulation of α-actinin 4 in the autophagosomes instead of secretion.

Furthermore, when examining the colocalization of 14-3-3ε and autophagosomes, we observed that the percentage of 14-3-3ε-containing autophagosomes per cell was lower than the percentage of α-actinin 4-containing autophagosomes in all analyzed cell lines (Fig. 7F). Although the percentage of 14-3-3ε-containing autophagosomes per cell was unaffected in all analyzed cell lines, the colocalization coefficients were markedly decreased in both AGR2-KO cells compared to CMT-U27e (Fig. 7G). Collectively, these data imply that the AGR2 knockout may hinder the translocation of a small portion of 14-3-3ε to the autophagosomes, resulting in a reduced release of 14-3-3ε.

### Extracellular 14-3-3ε and α-actinin 4 confer the chemotaxis effect of AGR2-modulated CM

**Fig. 8:**
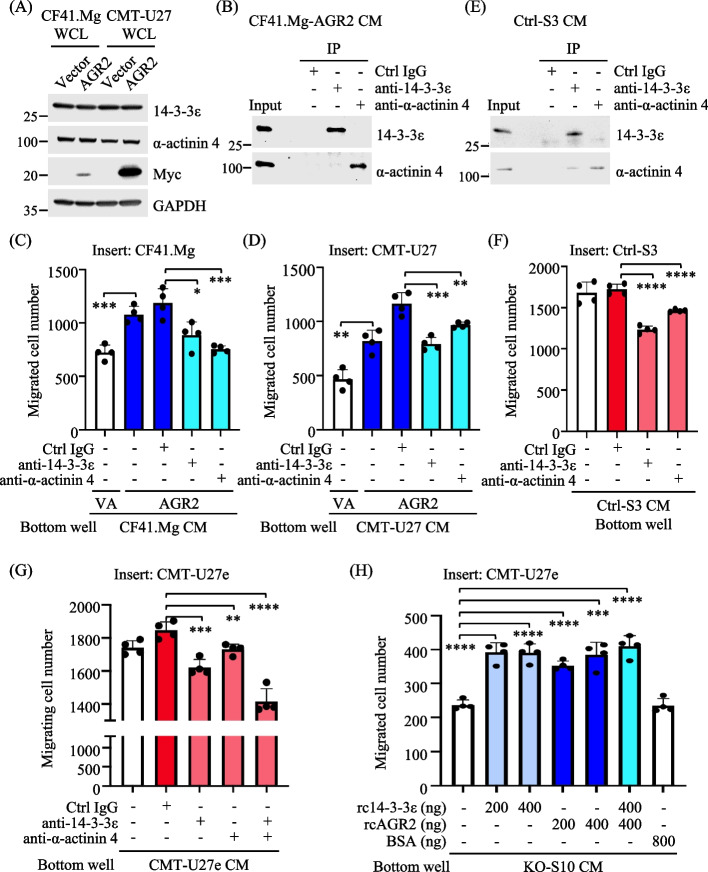
Extracellular 14-3-3ε and α-actinin 4 conferred the chemotaxis effect of AGR2-modulated CM. **A** WCL and CM samples of CF41.Mg or CMT-U27 transfected with pcDNA3.1-myc.His-AGR2 or the mock control were collected as described previously. Levels of indicated proteins in individual WCL samples were analyzed by immunoblotting. **B** CM of AGR-expressing CF41.Mg was deprived of 14-3-3ε or α-actinin 4 by immunoprecipitation (IP). Input CM and IP protein products were analyzed by immunoblotting. **C**, **D** CM samples before and after IP were subjected to a transwell migration assay. **E** CM samples of Ctrl-S3 cells grown at sub-confluency were collected and applied to IP, followed by immunoblotting analysis as described in **B**. **F**, **G** CM samples of Ctrl-S3 (**F**) or CMT-U27e (**G**) cells supplied with the indicated antibodies (3 μg each) were placed in the bottom well for a transwell migration assay. **H** CM of KO-S10 supplied with rcAGR2, rc14-3-3ε, or BSA at the indicated concentration was placed in the bottom well for a transwell migration assay. Data of all transwell migration assays were presented as the mean + SD (*n* = 4), and a two-tailed unpaired t-test determined statistical significance. *p < 0.05; **p < 0.01; ***p < 0.001; ****p < 0.0001

To confirm that the chemotaxis activity of AGR2-modulated CM was associated with 14-3-3ε and α-actinin 4, we ectopically expressed AGR2 in both CF41.Mg and CMT-U27 cells. Subsequently, we conducted immunoprecipitation (IP) to deplete 14-3-3ε or α-actinin 4 from the CM or inhibited 14-3-3ε or α-actinin 4 in the CM by incubating with specific antibodies. Immunoblotting analysis confirmed the ectopic expression of AGR2 in both cell lines (Fig. 8A). We then subjected the CM from AGR2-expressing cells to IP using specific antibodies against 14-3-3ε or α-actinin 4. As depicted in Fig. 8B, 14-3-3ε or α-actinin 4 in the CM was successfully immunoprecipitated. We then applied the CM samples, both with and without the IP-based depletions, to a transwell migration assay. It was evident that depletion of 14-3-3ε or α-actinin 4 from the CM of AGR2-expressing CF41.Mg or CMT-U27 significantly diminished the CM-driven chemotaxis (Fig. 8C and D).

We then sought to confirm the contribution of 14-3-3ε and α-actinin 4 in the chemotaxis activity of the CM from Ctrl-S3 by inhibiting these proteins with specific antibodies. Initially, we conducted IP to validate the effectiveness of the antibodies in capturing the target proteins within the CM (Fig. 8E). Subsequently, we applied the CM from Ctrl-S3, supplemented with or without anti-14-3-3ε or anti-α-actinin 4, to a transwell migration assay. The results confirmed that inhibiting 14-3-3ε or α-actinin 4 led to a reduction in the CM-driven chemotaxis (Fig. 8F). We further explored whether 14-3-3ε and α-actinin 4 cooperated in the chemotaxis activity. For this purpose, we applied the CM from CMT-U27e to a transwell migration assay, incubating the CM with anti-14-3-3ε, anti-α-actinin 4, or both. As shown in Fig. 8G, inhibiting either 14-3-3ε or α-actinin 4 resulted in a reduction in the CM-mediated chemotaxis. Moreover, inhibiting both 14-3-3ε and α-actinin 4 amplified the reduction in the chemotaxis activity, indicating their cooperative roles in this event. Of note, a portion of α-actinin 4 was co-immunoprecipitated with 14-3-3ε from the CM (Fig. 8B and E), suggesting that these proteins may cooperate via a protein–protein interaction.

Considering the availability of recombinant proteins, we first validated the chemotaxis-driven effect of 14-3-3ε in the CM. We supplemented the CM from AGR2-KO cells (KO-S10) with varying concentrations of recombinant 14-3-3ε proteins (rc14-3-3ε). As shown in Fig. 8H, the addition of rc14-3-3ε to the CM increased the CM-driven chemotaxis, compared with the blank control or the CM supplemented with bovine serum albumin (BSA). In comparison, the addition of rcAGR2 to the CM also enhanced the chemotaxis activity. Notably, we did not observe a dose-dependent or cooperative effect between the CM supplemented with rc14-3-3ε, rcAGR2, or both.

In conclusion, our findings provide evidence that extracellular 14-3-3ε and α-actinin 4 collaboratively contribute to the chemotaxis activity of AGR2-modulated CM. This phenomenon occurs in parallel with the presence of extracellular AGR2.

### Levels of 14-3-3ε and α-actinin 4 increase in CMT tissue-immersed PBS and sera of CMT-afflicted dogs

**Fig. 9:**
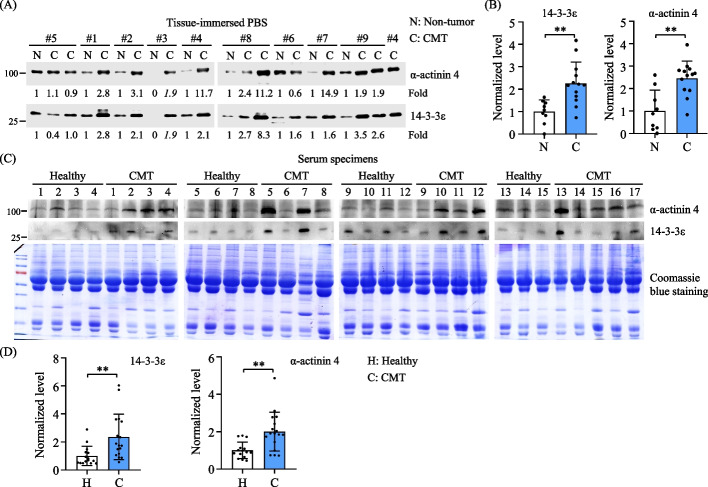
Elevated levels of 14-3-3ε and α-actinin 4 in CMT tissue-immersed PBS and sera of CMT-afflicted dogs. **A** Paired samples of CMT tissues (labeled C) and non-involved mammary gland tissues (labeled N) were obtained from nine female dogs afflicted with CMT. The tissue-immersed PBS samples were collected after one-hour incubation, and the tissue-released proteins were precipitated with TCA for immunoblotting analysis (10 μg of each sample) of 14-3-3ε and α-actinin 4. The CMT sample of patient #4 was replicated in two blots. Results were quantified and presented as fold changes of CMT over paired non-tumor samples. For patient#3, a fold change of CMT was calculated by comparing to the mean of all non-tumor samples. **B** Elevated levels of 14-3-3ε and α-actinin 4 were observed in CMT tissue-immersed PBS compared with non-tumor tissue-immersed samples. Data from all samples were normalized by the mean of total non-tumor samples. Statistical analysis was conducted using the Mann–Whitney U test. **p < 0.01. **C** Sera were collected from 17 female dogs afflicted with CMT and 15 age-matched healthy female dogs. Each serum sample (1 μL) was diluted in PBS, mixed with 4 × sampling buffer and subjected to SDS-PAGE with two gels. One gel was stained with Coomassie Brilliant Blue G-250, while the other was used for immunoblotting analysis. To quantify the levels of 14-3-3ε and α-actinin 4 in serum samples, the blot intensity of 14-3-3ε or α-actinin 4 was divided by the intensity of the entire lane of proteins stained with Coomassie Brilliant Blue for each sample. The resulting ratio was then normalized by the mean ratio of all healthy samples to acquire a normalized level for comparison. **D** Elevated levels of 14-3-3ε and α-actinin 4 were observed in sera from CMT-afflicted dogs compared with those in sera from healthy dogs. Statistical analysis was conducted with the Mann–Whitney U test. **p < 0.01

To assess the clinical relevance of the release of 14-3-3ε and α-actinin 4, we initially obtained paired samples of CMT tissues and non-involved mammary gland tissues from 9 female dogs afflicted with CMT (supplementary Table S3). Tissue-immersed PBS samples were collected to extract the tissue-released proteins for subsequent immunoblotting analysis of 14-3-3ε and α-actinin 4. The results revealed elevated levels of both 14-3-3ε and α-actinin 4 in CMT tissue-immersed PBS compared with the paired non-tumor sample (Fig. 9A). Overall, CMT tissues released significantly higher levels of 14-3-3ε and α-actinin 4 than non-tumor tissues (Fig. 9B).

Next, we investigated the presence of 14-3-3ε and α-actinin 4 in canine sera and its correlation with CMT. Immunoblotting analysis was conducted to detect these proteins in sera collected from 17 female CMT-afflicted dogs and 15 age-matched healthy female dogs (Table S3, Additional file 3). To quantify the levels of 14-3-3ε and α-actinin 4 in serum samples, the blot intensity of 14-3-3ε or α-actinin 4 was divided by the intensity of the entire lane of proteins stained with Coomassie blue for each sample (Fig. 9C). The resulting ratio was then normalized by the mean ratio of all healthy samples to acquire a normalized level for comparison. The results confirmed elevated levels of 14-3-3ε and α-actinin 4 in the sera of CMT-afflicted dogs compared with those in the sera of healthy dogs (Fig. 9D), suggesting clinical relevance of CMT cell-released 14-3-3ε and α-actinin 4.

## Discussion

**Fig. 10:**
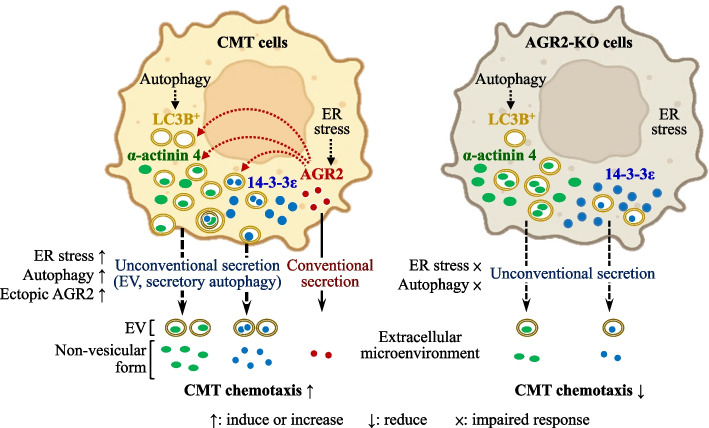
Schematic illustration of a proposed model for how AGR2 controls the release of 14-3-3ε and α-actinin 4 to promote chemotaxis of CMT cells. AGR2 functions as a stress sensor, regulating proteostasis by controlling the release of unconventional secretory proteins. Upon serum starvation, tunicamycin-induced ER stress, or rapamycin-induced autophagy, AGR2 expression promotes the release of 14-3-3ε and α-actinin 4 into the extracellular microenvironment, thereby enhancing chemotaxis in CMT cells. This controlled release of 14-3-3ε and α-actinin 4 by AGR2 involves extracellular vesicle (EV)-mediated delivery and secretory autophagy. Depletion of AGR2 leads to a reduced release of 14-3-3ε and α-actinin 4 in response to serum starvation, ER stress, or autophagy induction. Additionally, the absence of AGR2 can result in diminished uptake of 14-3-3ε within the LC3B^+^ autophagosome and impaired export of α-actinin 4 through the LC3B^+^ autophagosome

In this study, we discover a novel role for AGR2 in facilitating the unconventional secretion of 14-3-3ε and α-actinin 4, alongside its conventional secretion. This process modulates a pro-chemotaxis extracellular microenvironment (Fig. 10). AGR2 has previously been shown to regulate the folding and secretion of its client proteins, e.g., MUC2 and MUC5, through the classical secretory pathway. Our present study unveils the interconnections between the intracellular function of AGR2 and the regulation of unconventional protein secretion encompassing multiple routes (e.g., EV and secretory autophagy) for externalizing proteins that lack a signal peptide, such as 14-3-3ε and α-actinin 4. Unconventionally secreted 14-3-3ε and α-actinin 4 are functional as paracrine (Fig. 8), and their extracellular roles could be distinct from their intracellular functions, thereby augmenting their oncogenic impacts.

Moreover, we confirmed the AGR2-modulated unconventional release of 14-3-3ε and α-actinin 4 in both CMT and human breast cancer lines representing diverse molecular subtypes, including CMT-U27 and CMT-U27e (ER^−^/HER2^+^, E-cadherin^+^/Vimentin^−^), CF41.Mg and DMGT (ER^+^/HER2^−^, E-cadherin^−^/Vimentin^+^), MCF7 (ER^+^/HER2^+^, E-cadherin^+^/Vimentin^−^), and MDA-MB-231 (ER^−^/HER2^−^, E-cadherin^−^/Vimentin^+^). These findings suggest the influence of AGR2 on the release of 14-3-3ε and α-actinin 4 may transcend specific cell types, potentially affecting a diverse range of cellular contexts.

The molecular mechanism underlying how AGR2 regulates the release of 14-3-3ε and α-actinin 4 currently remains elusive. Apparently, the release of 14-3-3ε and α-actinin 4 is uncoupled from the conventional secretion of AGR2. AGR2 secretion is inhibited by brefeldin A, which inhibits the ER-Golgi vesicle transport, while the release of 14-3-3ε and α-actinin 4 is enhanced by brefeldin A instead (Fig. S6C). In agreement with the notion that the unconventional protein secretion is often linked to cellular stresses [70, 73], the release of 14-3-3ε and α-actinin 4 is responsive to tunicamycin-triggered ER stress (Fig. 6B, D; Fig. S2B, S2C), serum starvation, and rapamycin-induced autophagy (Fig. 6F; Fig. S2C) that also cause autophagosomes to form (Fig. 7A–C). Under these stress conditions, however, depletion of AGR2 results in a significant reduction in the release of 14-3-3ε and α-actinin 4 (Fig. 6B, F) and the formation of autophagosomes (Fig. 7C). In contrast, ectopic expression of AGR2 intensifies this stress-related protein secretion (Fig. 6D; Fig. S2B), which can be diminished by addition of an autophagy inhibitor, 3-MA (Fig. 6H). Furthermore, the absence of AGR2 likely hinders the engulfment of 14-3-3ε into the autophagosome (Fig. 7G) and the release of α-actinin 4 through the autophagosome, leading to reduced 14-3-3ε translocation and increased α-actinin 4 accumulation in these LC3B-labeled compartments, respectively (Fig. 7D, E). Collectively, these findings suggest that AGR2 may contribute to maintaining balanced proteostasis by regulating the unconventional protein secretion, partially involving the autophagic flux, i.e., secretory autophagy [68], as depicted in the proposed model (Fig. 10).

Although 14-3-3ε and α-actinin 4 have been identified as EV contents, our study demonstrates that AGR2 controls the release of 14-3-3ε and α-actinin 4 via both EVs and non-vesicular routes (Fig. S3D, S3E, and Fig. S4E), with the latter delivering the majority of extracellular 14-3-3ε and α-actinin 4. As a result, non-vesicular 14-3-3ε and α-actinin 4 mainly confer the pro-chemotaxis effects (Fig. S3F), which are impaired by depleting 14-3-3ε or α-actinin 4 from the CM (Fig. 8C–G) but are increased by supplementing recombinant 14-3-3ε in the CM (Fig. 8H). It is speculated that cytosolic 14-3-3ε and α-actinin 4 may associate with a chaperon or a membrane receptor facilitating their translocation into the lumen of an autophagosome. Additionally, 14-3-3ε and α-actinin 4 may also be engulfed within an intra-lumen vesicle (ILV), which subsequently fuses with an autophagosome. The 14-3-3ε or α-actinin 4-containing autophagosome eventually fuse with the plasma membrane to release 14-3-3ε or α-actinin 4 in non-vesicular forms or within the ILVs corresponding to EVs, similar to the well-known unconventional secretion of IL-1β [74]. On the other hand, we observed that only 20% of autophagosomes per cell encompass 14-3-3ε (Fig. 7D), compared with the observation that 40% of autophagosomes contain α-actinin 4 (Fig. 7F). This leads us to hypothesize that 14-3-3ε may be preferentially translocated into the endosome or lysosome, which directly fuses with the plasma membrane to release soluble 14-3-3ε, similar to the unconventional secretion of the fatty acid binding protein 4 (FABP4) [75]. Rationally, both α-actinin 4 and 14-3-3ε protein sequences possess in silico predicted KFERQ-like motifs, as identified in IL-1β and FABP4 protein sequences, which bind to the chaperon or the receptor required for facilitating their translocation into the relevant compartments. Those motifs include ^52^QRKTF^56^, ^213^DKLRK^217^, ^214^KLRKD^218^, ^361^QTKLR^365^, ^416^EKFRQ^420^, ^753^QILTR^757^ in α-actinin 4, and ^222^QLLRD^226^ in 14-3-3ε, as predicted by using KFERQ finder V0.8 (https://rshine.einsteinmed.edu/). Our study suggests that 14-3-3ε and α-actinin 4 could be unconventionally secreted via chaperon-mediated autophagy, and AGR2 appears to play active roles in multiple steps during this process. Further investigation is warranted due to the clinical significance of AGR2 and unconventionally secreted oncogenic proteins.

This study has certain limitations. Firstly, we observed the effect of AGR2-modulated secretome on chemotaxis using a simple culture system in its current state. To rigorously validate these findings, transitioning to a three-dimensional culture of CMT cells co-incubated with recipient cells or an in vivo model is crucial. Moreover, there is currently a lack of clinical correlations between expression levels of AGR2, extracellular levels of 14-3-3ε and α-actinin 4, and the status of CMT. To assess the clinical relevance, we validated elevated levels of 14-3-3ε and α-actinin 4 in the PBS containing CMT tissue-released proteins as well as in sera from CMT-afflicted dogs, utilizing a small cohort of specimens (Fig. 9). These results underscore the importance of further endeavors to analyze the correlation between the expression levels of AGR2, 14-3-3ε and α-actinin 4 in tumor cells, as well as the levels of serum AGR2, 14-3-3ε and α-actinin 4 under stress conditions, using specimens from an expanding number of CMT patients.

Recently, 14-3-3ε and α-actinin 4 have been identified in cancer-derived EVs in sera or plasma of cancer patients [61, 62], while their extracellular oncogenic roles await thorough investigation. A related study reveals that human colorectal cancer cells release vesicles containing 14-3-3ξ along with β-catenin, which can be taken up by neighboring cells and enhance their migration ability via activation of the Wnt signaling [76]. Intracellularly, 14-3-3 proteins are known for their regulation of multiple signaling pathways that govern critical processes in cancer via interacting with various partners. The changes in environmental conditions will result in the loss of homeostatic 14-3-3 interactions and trigger new interactions [52]. However, whether or how extracellular 14-3-3ε reaches proteins other than its intracellular partners to exert cancer-promoting functions remains unclear. In this study, we demonstrate that 14-3-3ε associates with α-actinin 4 in the CM (Fig. 8B, E) to promote cell chemotaxis synergistically (Fig. 8G). It has been shown that extracellular 14-3-3 proteins bind to aminopeptidase N (APN, CD13) and thereby induce the transcription of matrix metalloproteinase genes [63, 77]. Further investigation is needed to elucidate if extracellular 14-3-3ε and α-actinin 4 co-opt to bind to APN or other surface receptors of recipient cells in the microenvironment, and how EV-delivered 14-3-3ε and α-actinin 4 transduce relevant signaling pathways in the recipient cells to modulate the tumor microenvironment.

## Conclusions

In summary, this present study provides new insights into the roles of AGR2 in coping with the complex interplay between ER stress, pro-survival unfolded protein response (UPR), autophagy, and unconventional secretion of oncogenic proteins, such as 14-3-3ε and α-actinin 4. Sustained ER stress and autophagy have been considered mechanisms underlying tumor progression, metastasis, and resistance to anticancer agents [70, 78]. In this perspective, unconventionally released proteins and EV contents from cancer cells due to stress could be oncogenic and favor a pro-oncogenic tumor microenvironment. Further investigation into the impact of extracellular 14-3-3ε and α-actinin 4 on cancer cell phenotypes and therapy responses, coupled with an analysis of their concentrations in cancer patients, may suggest new avenues in the management and therapy of cancers. Addressing secreted 14-3-3ε and α-actinin 4 in addition to AGR2 in the microenvironment could offer novel insights into cancer treatment strategies.

### Supplementary Information

Supplementary Material 1: **Table S1.** CMT-U27_protein ID and quantification.

Supplementary Material 2: **Table S2.** CF41.Mg_protein ID and quantification.

Supplementary Material 3: **Table S3.** Signalments of healthy female dogs and dogs with malignant mammary tumors

Supplementary Material 4: **Fig. S1.** Ectopic expression of AGR2 modulated extracellular milieu of several cancer cell lines to promote cell chemotaxis. The CMT cell line DMGT (A, B) was transfected with pcDNA3.1-myc.His-AGR2 or the mock vector and grown in 1% FBS-containing DMEM for 24 h. Additionally, the human breast adenocarcinoma cell line MDA-MB-231 (C, D) or MCF7 (E, F) was transfected with an expression vector for human AGR2 under similar condition settings. (A, C, E) Whole-cell lysates (WCL) of the transfectants were analyzed by immunoblotting to confirm the expression of Myc-tagged AGR2. Conditioned media (CM) of the transfectants were collected and placed in the bottom well for a transwell migration assay. Cells in the insert were fixed for image acquisition using an epifluorescence microscope with a 10 × objective. (B, D, F) The number of migrated cells was counted and presented as the mean + SD of three independent experiments. Statistical significance was determined by a two-tailed unpaired t-test. *, *p* < 0.05; **, *p* < 0.01. **Fig. S2.** AGR2 modulated the release of 14-3-3ε and α-actinin 4 in several cancer cell lines. (A) MCF7 transfected with pcDNA3.1-myc.His-hAGR2 or the mock control were subsequently cultured in DMEM containing 1% FBS for 24 h. The levels of the indicated proteins in WCL and CM were analyzed by immunoblotting. (B) DMGT or MDA-MB-231 transfected with pcDNA3.1-myc.His-AGR2, pcDNA3.1-myc.His-hAGR2, or the mock control were subsequently cultured in DMEM containing 1% FBS for 14 h with or without addition of 50 nM tunicamycin (Tm) following transfection. The levels of the indicated proteins in WCL and CM were analyzed by immunoblotting using antibodies specific to indicated proteins. (C) DMGT, MDA-MB-231, or MCF7 were cultured in 1% FBS-containing DMEM and treated with 100 nM rapamycin (Rm) or 40 µM chloroquine (CQ) for 20 h. The levels of the indicated proteins in WCL and CM were analyzed by immunoblotting. These results represent data from three independent experiments. **Fig. S3.** Extracellular vesicles were dispensable for the chemotaxis conferred by AGR2-modulated CM. (A) Schematic diagram for comprehensive isolation of extracellular vesicles (EVs) with varied sizes by differential ultracentrifugation. (B) Nanoparticle tracking analysis (NTA) was performed on the isolated large (l) EVs and small (s) EVs derived from control Ctrl-S3 cells. (C) Transmission electron microscopy (TEM) images depict the isolated lEV and sEV (indicated by arrowheads). (D) The CM samples before and after EV depletion, and the WCL of CMT-U27e, Ctrl-S3, KO-S10, and KO-S4, respectively, were analyzed by immunoblotting with antibodies specific to the indicated proteins. CD9 and CD63 are considered markers for exosomes with smaller sizes, and Annexin A1 is considered a marker for microvesicles with larger sizes. (E) Isolated lEVs and sEVs originating from individual cells were analyzed by immunoblotting with specific antibodies as indicated. (F) CM samples with and without EV depletion (denoted EV-present and EV-depleted CM, respectively) were applied to a transwell migration assay. Data are presented as the mean + SD of three independent experiments. **Fig. S4.** AGR2 knockout led to a reduction in the EV-delivered 14-3-3ε and α-actinin 4 and the EV-mediated chemotaxis. (A) The schematic diagram for isolation of EV-enriched fractions by the size exclusion chromatography (SEC) using the qEV Isolation Columns. (B) CM from CMT-U27e cells was first concentrated with VivaSpin 20 (> 100 kD) and applied to the SEC. Individual fractions collected throughout the SEC, along with the concentrated CM (> 100 kD) and the flowthrough (< 100 kD) subsequently concentrated for the second time, were analyzed by immunoblotting with antibodies specific to 14-3-3ε, α-actinin 4, CD9, or Albumin. Albumin was used as an indicator for the EV-free protein fractions. (C) Nanoparticle tracking analysis (NTA) was conducted on the isolated EVs derived from CMT-U27e cells in qEV Fraction 1. (D) Transmission electron microscopy (TEM) images illustrate the isolated EVs (indicated by arrowheads) in qEV Fraction 1. (E) The CM fractions of CMT-U27e, Ctrl-S3, KO-S10, or KO-S4 collected during the SEC, together with the corresponding input CM (> 100 kD) and the flowthrough (< 100 kD), were analyzed by immunoblotting. (F) The EV-enriched fractions (Fraction 1) derived from individual cells were applied to a transwell migration assay. Data are presented as the mean + SD of three independent experiments, and the statistical significance was determined by a two-tailed unpaired t-test. *, *p* < 0.05; **, *p* < 0.01. **Fig. S5.** Experimental replicates of the immunoblotting analysis which are shown in Fig. 6 (A to D). (A, B) Experimental replicates of the immunoblotting data shown in Fig. 6A and Fig. 6B. (C, D) Experimental replicates of the immunoblotting data shown in Fig. 6C and Fig. 6D. **Fig. S6.** Experimental replicates of the immunoblotting analysis which are shown in Fig. 6 (E to H). (A, B) Experimental replicates of the immunoblotting data shown in Fig. 6E and Fig. 6F. (C, D) Experimental replicates of the immunoblotting data shown in Fig. 6G and Fig. 6H.

Supplementary Material 5: Original immunoblots in this study.

## Data Availability

All data generated or analyzed during this study are included in this published article and its supplementary information files. Original immunoblots are provided in Supplementary Material 5. The MS raw data for proteome analysis were deposited on the ProteomeXchange Consortium website (http://proteomecentral.proteomexchange.org) via the PRIDE partner repository [79], data set identifier: PXD047008.

## Abbreviations

ACTN4: Alpha-actinin 4
AGR2: Anterior gradient 2
CHOP: C/EBP homologous protein
CM: Conditioned media
CMT: Canine mammary tumors
ER: Endoplasmic reticulum
ER: Estrogen receptor
EV: Extracellular vesicle
HER2: Human epidermal growth factor receptor 2
LC3B: Microtubule-associated protein 1 light-chain 3B
mTOR: Mammalian target of rapamycin
SDS-PAGE: Sodium dodecyl sulfate–polyacrylamide gel electrophoresis
UPR: Unfolded protein response
YWHAE: Tyrosine 3-monooxygenase/tryptophan 5-monooxygenase activation protein epsilon
