# Supplementary material for: AGR2-mediated unconventional secretion of 14-3-3ε and α-actinin-4, responsive to ER stress and autophagy, drives chemotaxis in canine mammary tumor cells
Source: Cell Mol Biol Lett. 2024 May 31;29:84. doi: 10.1186/s11658-024-00601-w (PMC11140979; doi:10.1186/s11658-024-00601-w)
Supplement: Supplementary file 3 — Supplementary Material 3: Table S3. Signalments of healthy female dogs and dogs with malignant mammary tumors [file 11658_2024_601_MOESM3_ESM.pdf]

Table S3. Signalments of healthy female dogs and dogs with malignant mammary tumors

	Age (yr)	Breed	Spay	Tumor subtype	Stage
HA-1	12	Golden Retriever	Yes		
HA-2	15	Crossbreed	Yes		
HA-3	4	Dobermann	Yes		
HA-4	11	Golden Retriever	Yes		
HA-5	13	Chihuahua	Yes		
HA-6	6.1	Crossbreed	Yes		
HA-7	16	Crossbreed	Yes		
HA-8	3.5	Crossbreed	Yes		
HA-9	7	Shiba	No		
HA-10	4.5	Dachshund	Yes		
HA-11	4	Taiwanese Dog	Yes		
HA-12	10	Crossbreed	Yes		
HA-13	1.8	Crossbreed	Yes		
HA-14	10	Schnauzer	Yes		
HA-15	6	Jack Russel	Yes		
CMT-1	8	Maltese	Yes	Simple carcinoma	1
CMT-2	2	Golden Retriever	Yes	Intraductal papillary carcinoma	1
CMT-3	8	Poodle	No	Mixed-type carcinoma	1
CMT-4	12	Cocker spaniel	Yes	Complex carcinoma	1
CMT-5	8	Maltese	No	Complex carcinoma<br>Mixed-type carcinoma	1
CMT-6	11	Shih-Tzu	No	Mixed-type carcinoma	2
CMT-7	12	Crossbreed	Yes	Simple carcinoma	2
CMT-8	8	Maltese	No	Spindle cell carcinoma<br>Simple carcinoma	2
CMT-9	16	Crossbreed	Yes	Complex carcinoma<br>Simple carcinoma	3
CMT-10	13	Maltese	Yes	Simple carcinoma	3
CMT-11	10	Dachshund	No	Complex carcinoma	3
CMT-12	8	Poodle	No	Simple carcinoma	3
CMT-13	7	Toy Poodle	Yes	Intraductal papillary carcinoma<br>Ductal carcinoma<br>Complex carcinoma	4
CMT-14	8	Shih-Tzu	No	Intraductal papillary carcinoma	4
CMT-15	9	Maltese	Yes	Malignant myoepithelioma<br>Intraductal papillary carcinoma<br>Complex carcinoma	4
CMT-16	11	Crossbreed	No	Mixed-type carcinoma	5
CMT-17	3.5	Golden Retriever	Yes	Simple carcinoma	5
