# Supplementary figures and images for: AGR2-mediated unconventional secretion of 14-3-3ε and α-actinin-4, responsive to ER stress and autophagy, drives chemotaxis in canine mammary tumor cells
Source: Cell Mol Biol Lett. 2024 May 31;29:84. doi: 10.1186/s11658-024-00601-w (PMC11140979; doi:10.1186/s11658-024-00601-w)

Fig. S1

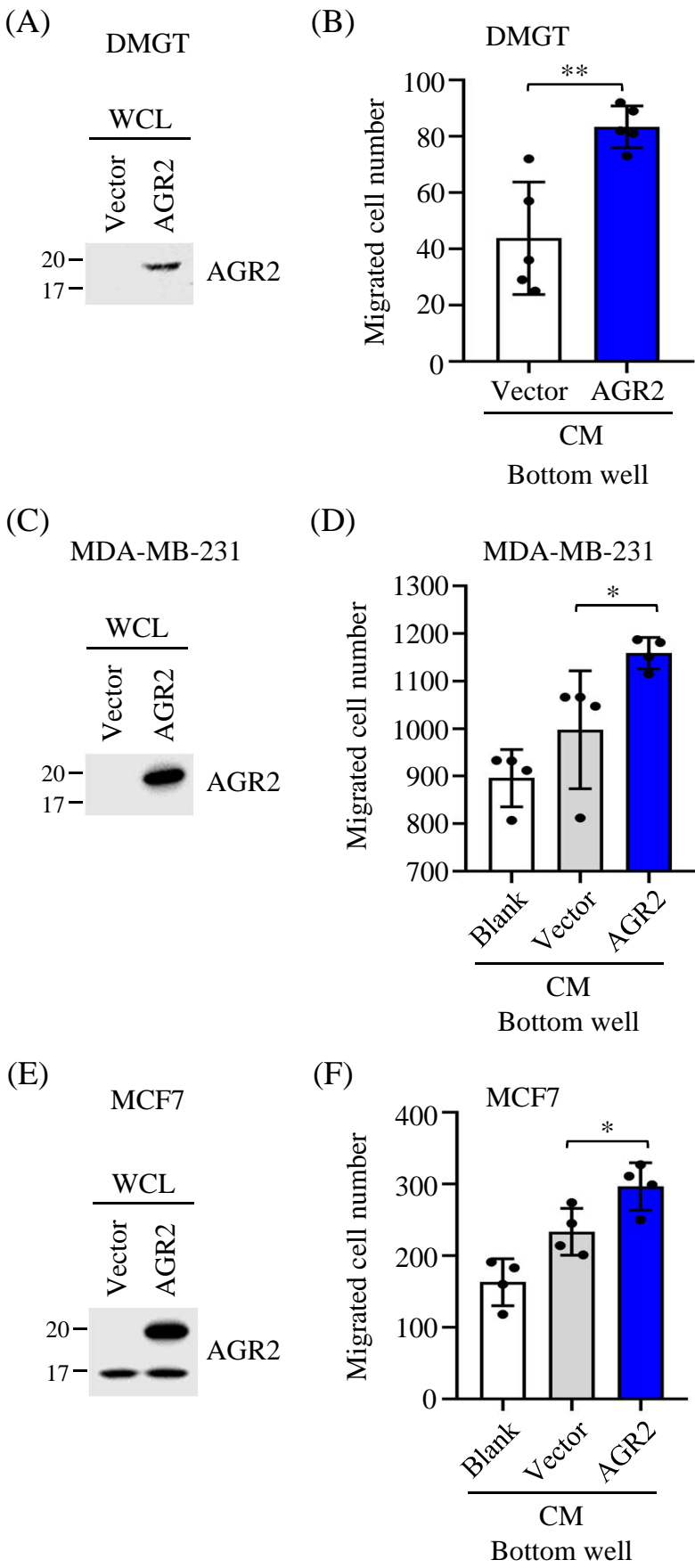

Fig. S2

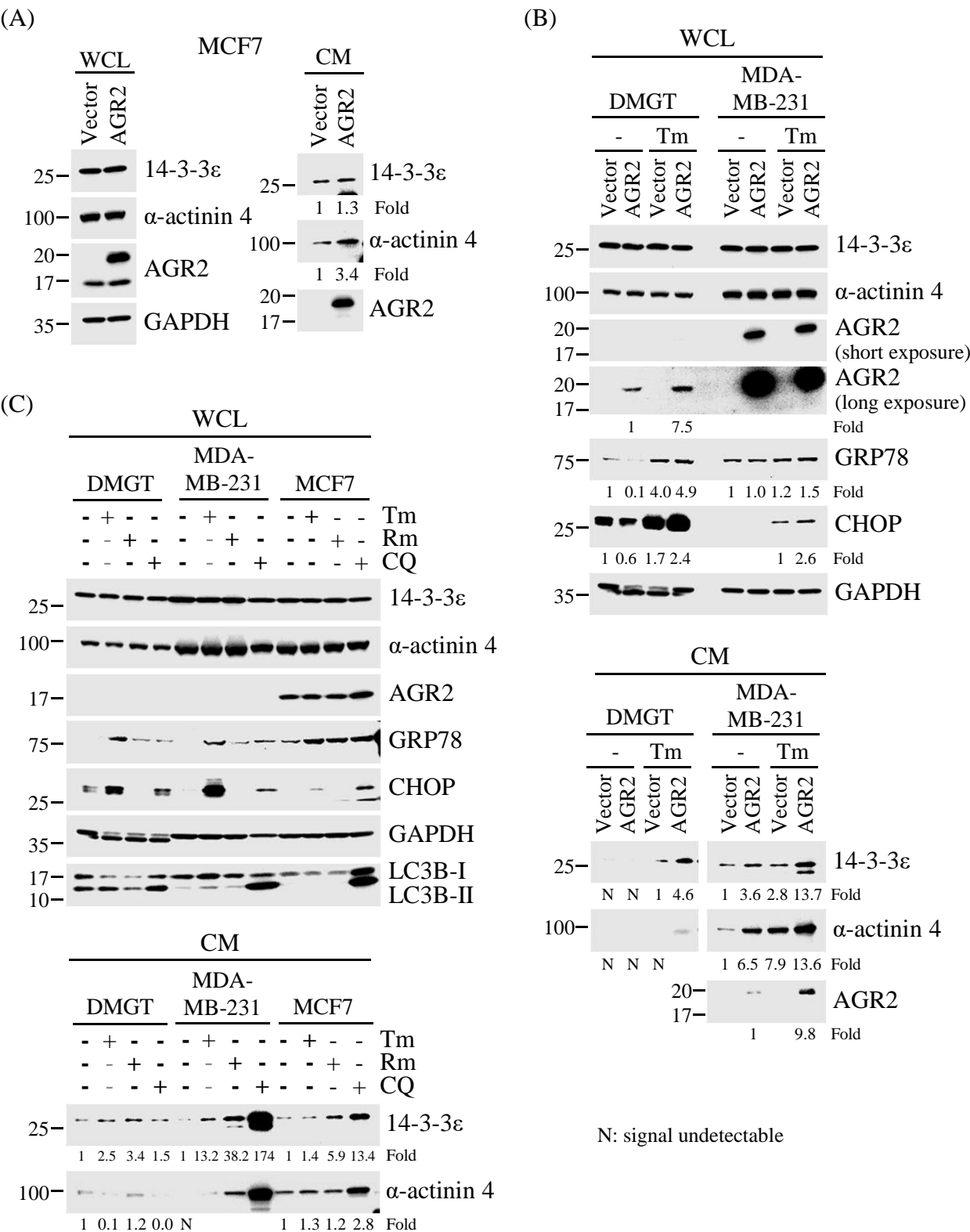

Fig. S3

(A)

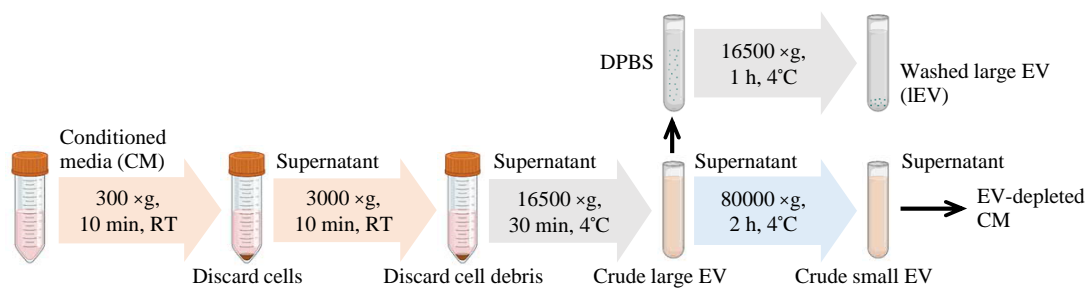

(B)

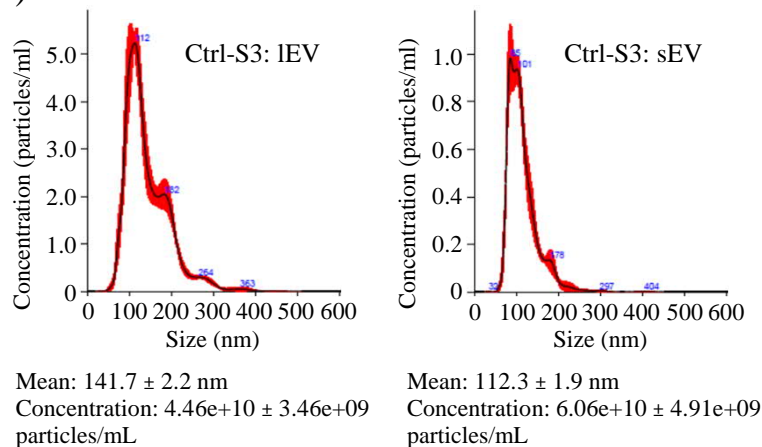

(C)

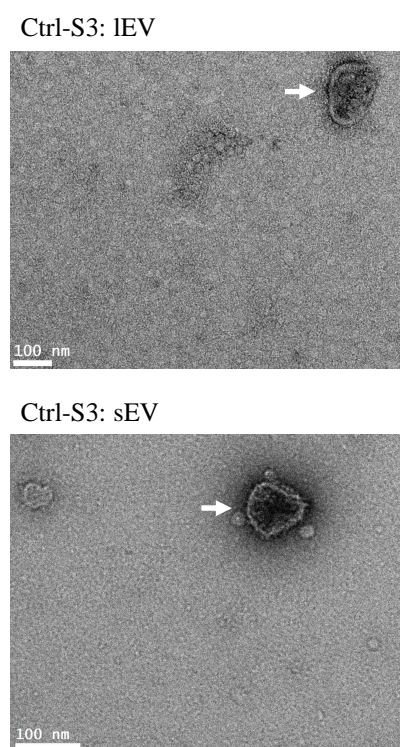

(D)

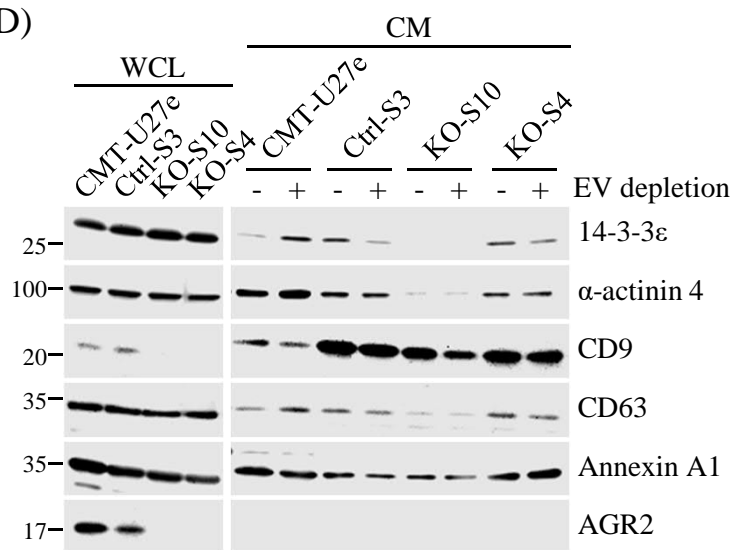

(E)

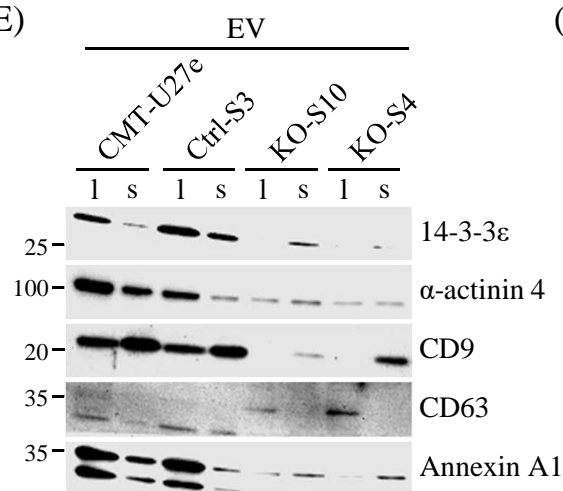

(F)

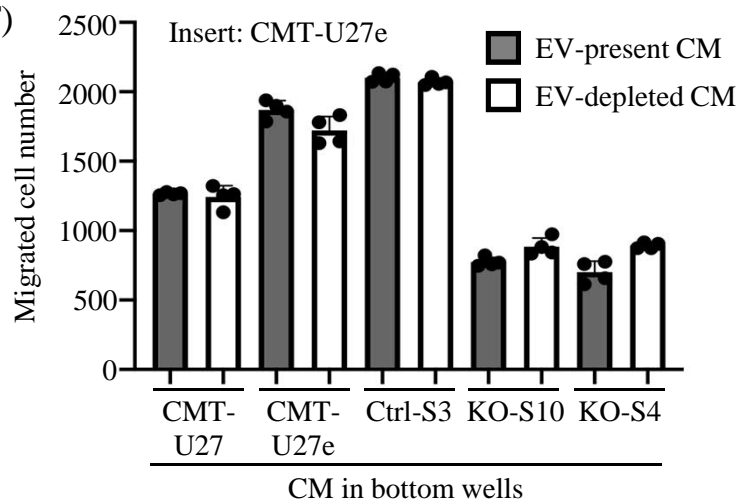

Fig. S4

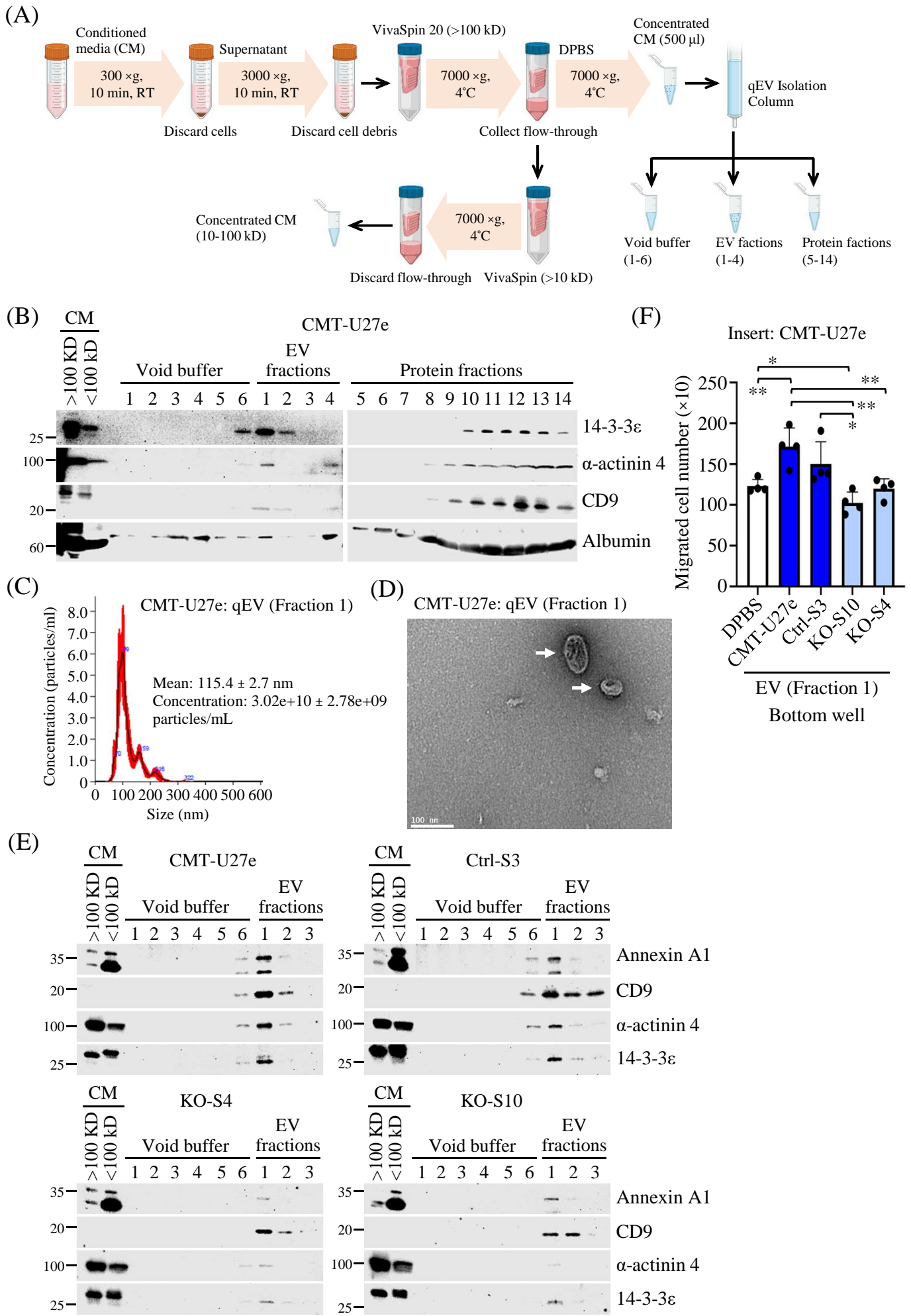

Fig. S5

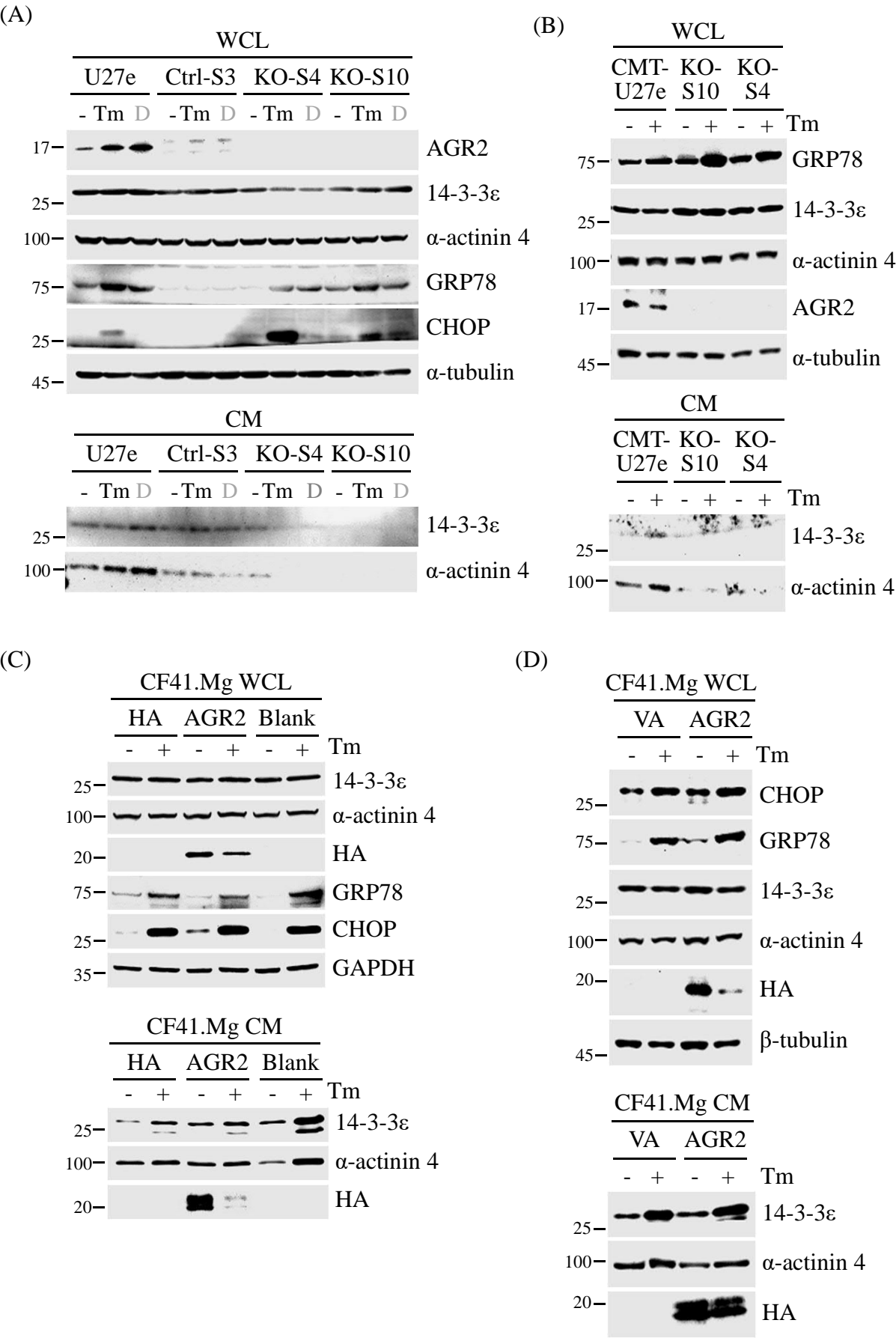

Fig. S6

(A)

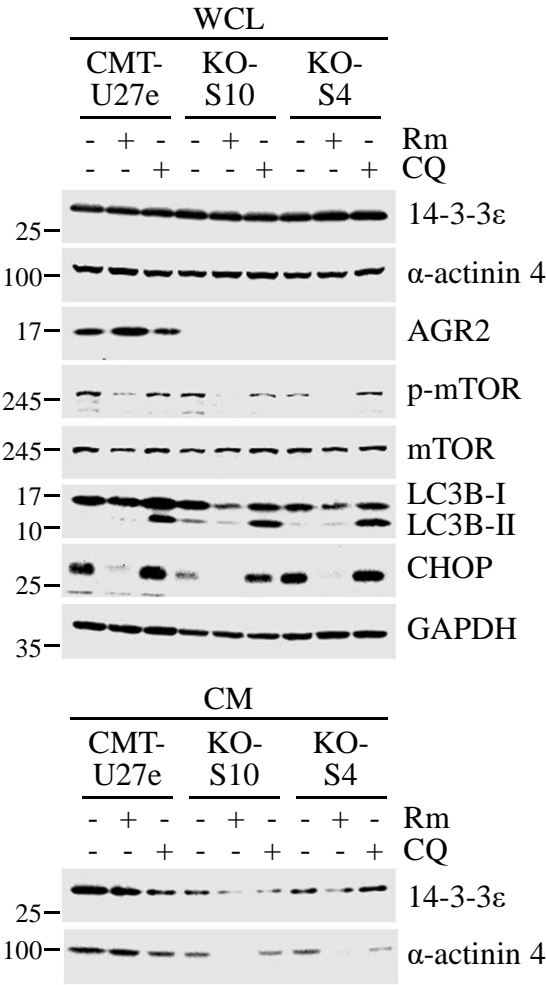

(B)

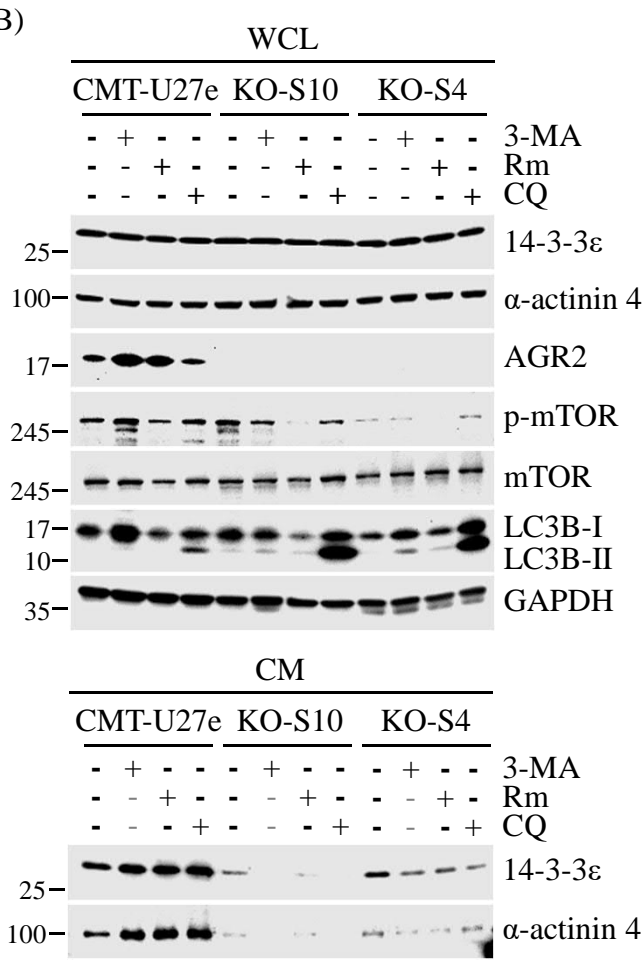

(C)

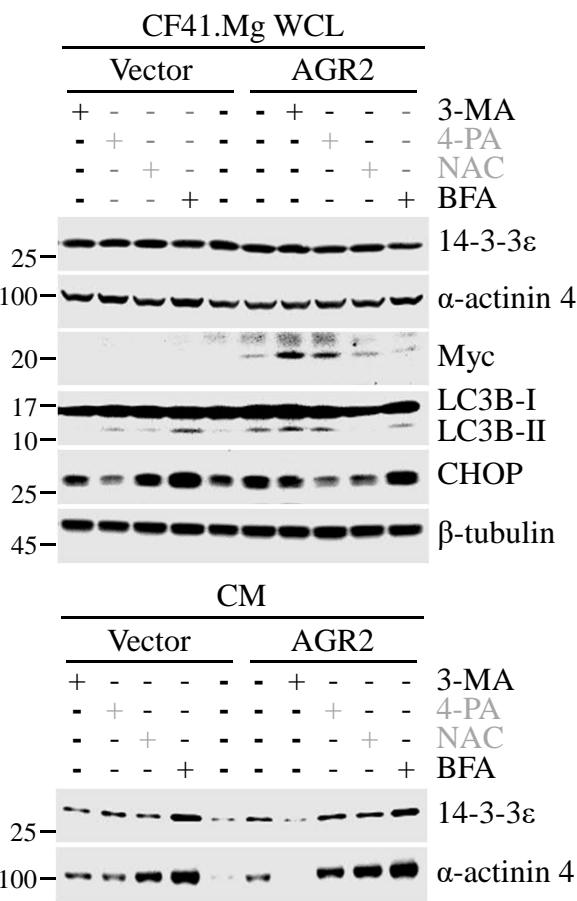

(D)

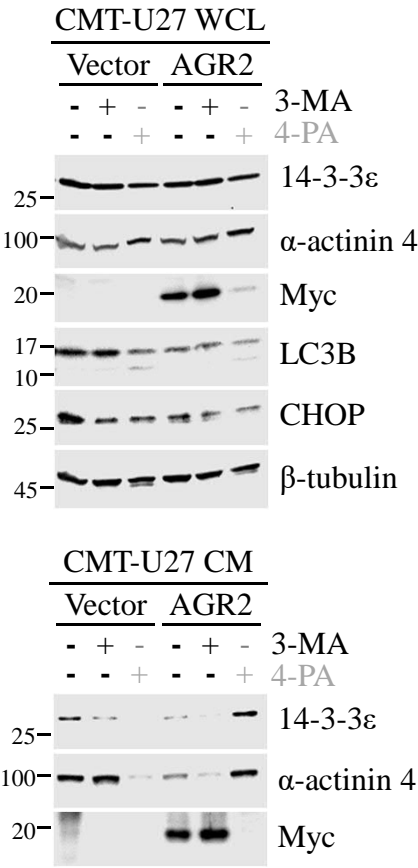

Supplement: Supplementary file 4 — Supplementary Material 4: Fig. S1. Ectopic expression of AGR2 modulated extracellular milieu of several cancer cell lines to promote cell chemotaxis. The CMT cell line DMGT (A, B) was transfected with pcDNA3.1-myc.His-AGR2 or the mock vector and grown in 1% FBS-containing DMEM for 24 h. Additionally, the human breast adenocarcinoma cell line MDA-MB-231 (C, D) or MCF7 (E, F) was transfected with an expression vector for human AGR2 under similar condition settings. (A, C, E) Whole-cell lysates (WCL) of the transfectants were analyzed by immunoblotting to confirm the expression of Myc-tagged AGR2. Conditioned media (CM) of the transfectants were collected and placed in the bottom well for a transwell migration assay. Cells in the insert were fixed for image acquisition using an epifluorescence microscope with a 10 × objective. (B, D, F) The number of migrated cells was counted and presented as the mean + SD of three independent experiments. Statistical significance was determined by a two-tailed unpaired t-test. *, p < 0.05; **, p < 0.01. Fig. S2. AGR2 modulated the release of 14-3-3ε and α-actinin 4 in several cancer cell lines. (A) MCF7 transfected with pcDNA3.1-myc.His-hAGR2 or the mock control were subsequently cultured in DMEM containing 1% FBS for 24 h. The levels of the indicated proteins in WCL and CM were analyzed by immunoblotting. (B) DMGT or MDA-MB-231 transfected with pcDNA3.1-myc.His-AGR2, pcDNA3.1-myc.His-hAGR2, or the mock control were subsequently cultured in DMEM containing 1% FBS for 14 h with or without addition of 50 nM tunicamycin (Tm) following transfection. The levels of the indicated proteins in WCL and CM were analyzed by immunoblotting using antibodies specific to indicated proteins. (C) DMGT, MDA-MB-231, or MCF7 were cultured in 1% FBS-containing DMEM and treated with 100 nM rapamycin (Rm) or 40 µM chloroquine (CQ) for 20 h. The levels of the indicated proteins in WCL and CM were analyzed by immunoblotting. These results represent data from [file 11658_2024_601_MOESM4_ESM.pdf]
