# Supplementary material for: AGR2-mediated unconventional secretion of 14-3-3ε and α-actinin-4, responsive to ER stress and autophagy, drives chemotaxis in canine mammary tumor cells
Source: Cell Mol Biol Lett. 2024 May 31;29:84. doi: 10.1186/s11658-024-00601-w (PMC11140979; doi:10.1186/s11658-024-00601-w)

Fig. 1A

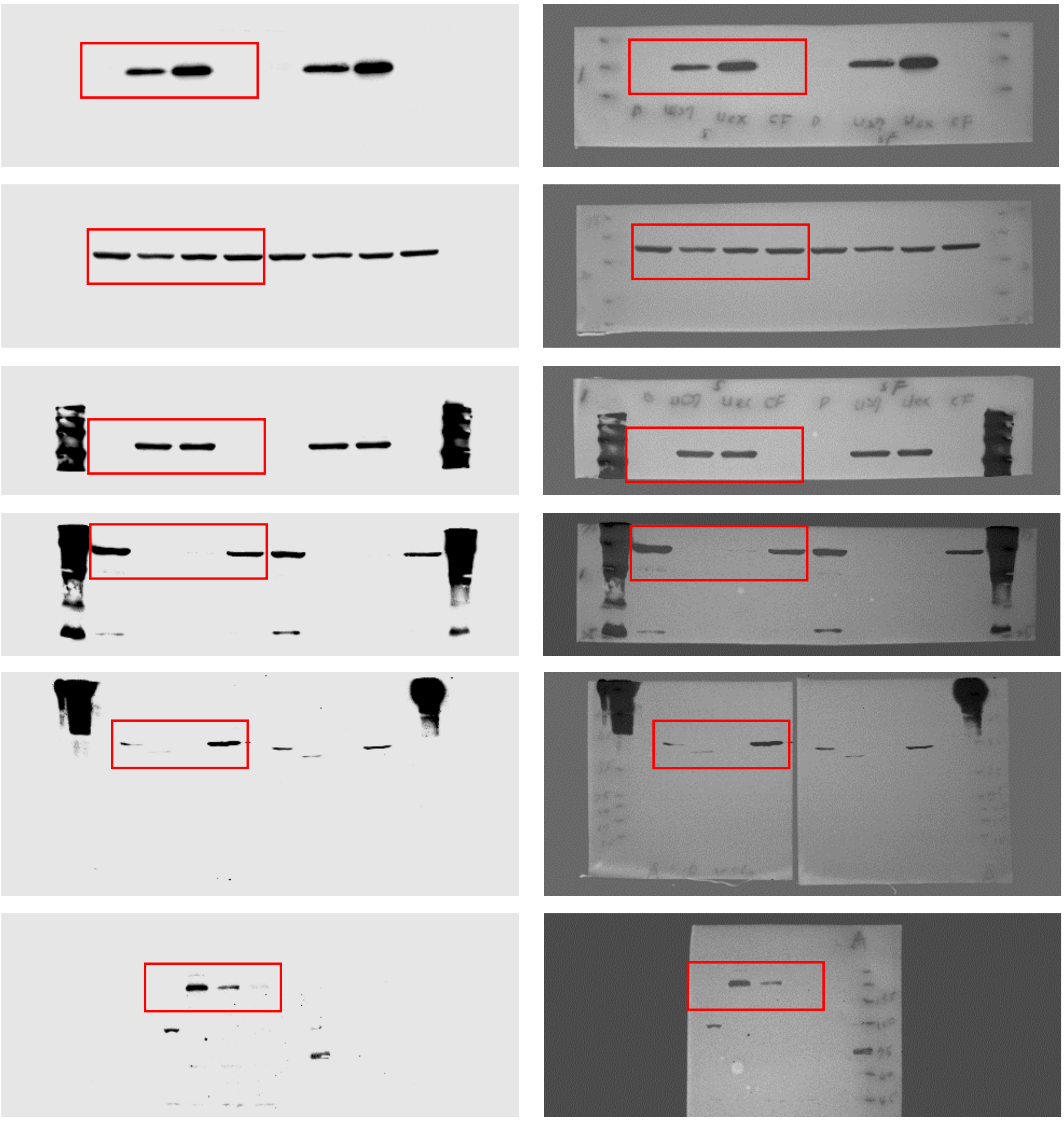

AGR2

β-tubulin

E-cadherin

Vimentin

ER-α

HER-2

Fig. 1B

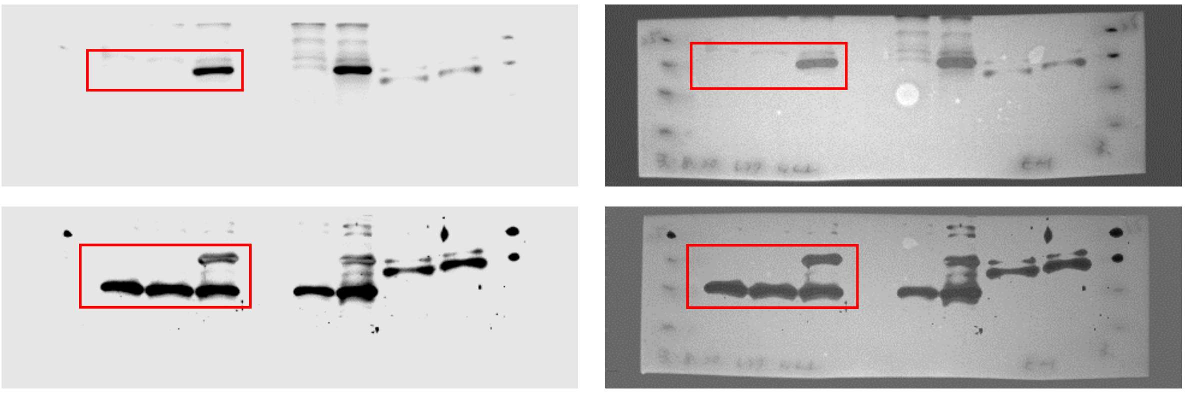

Myc

AGR2

Fig. 1C

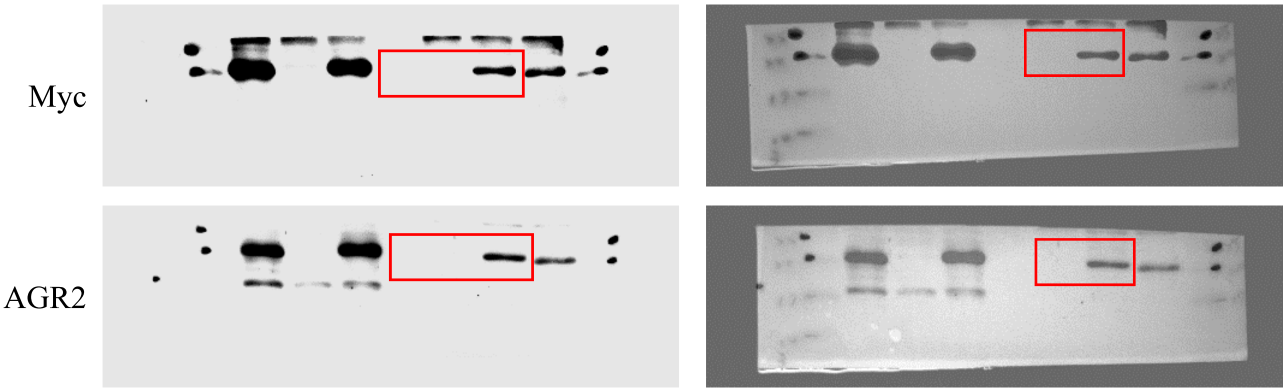

Fig. 1H

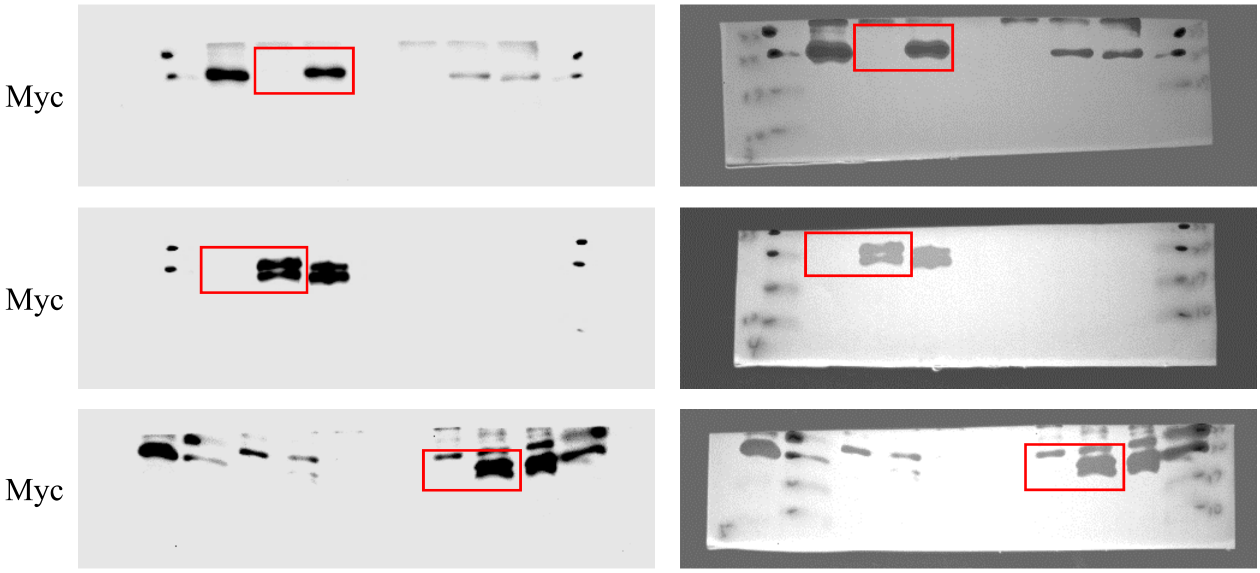

Fig. 3D

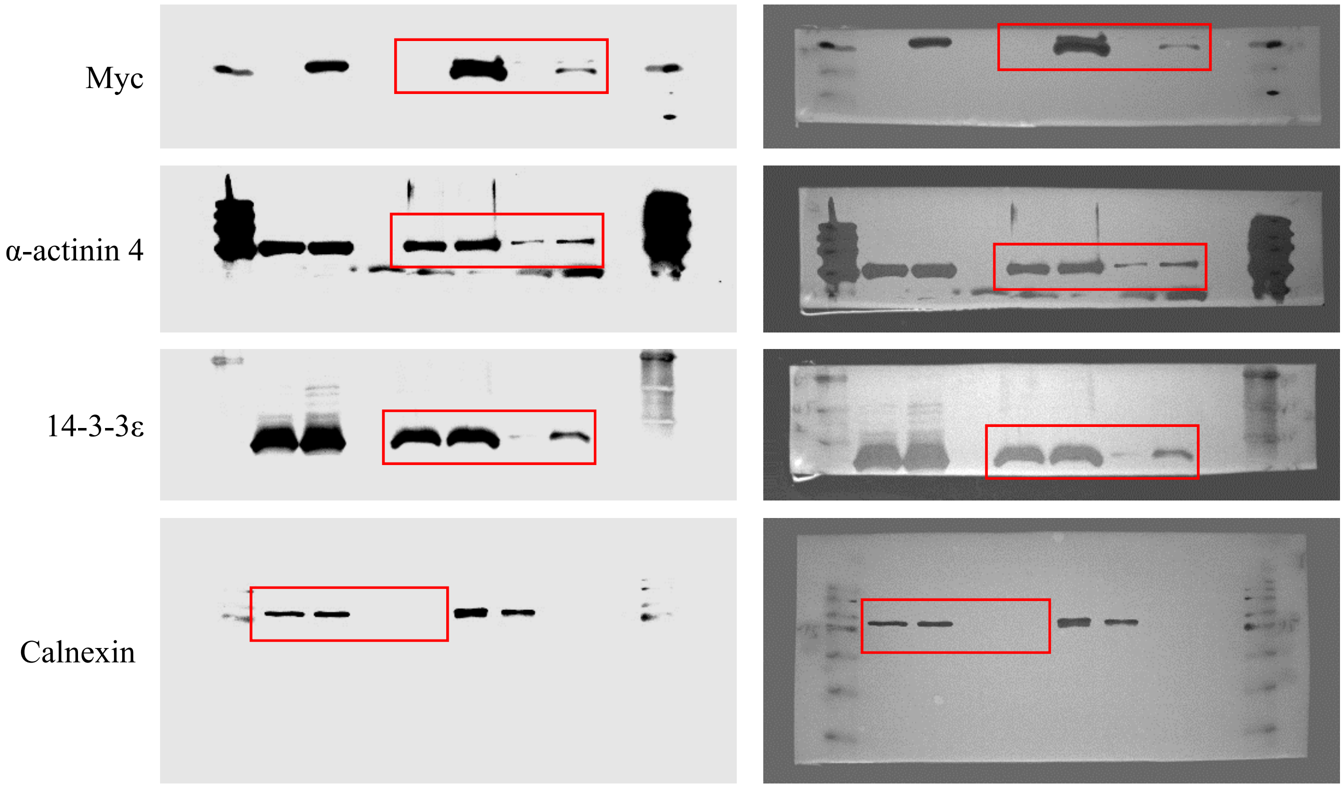
Myc
α-actinin 4
14-3-3ε
Calnexin

Fig. 3E

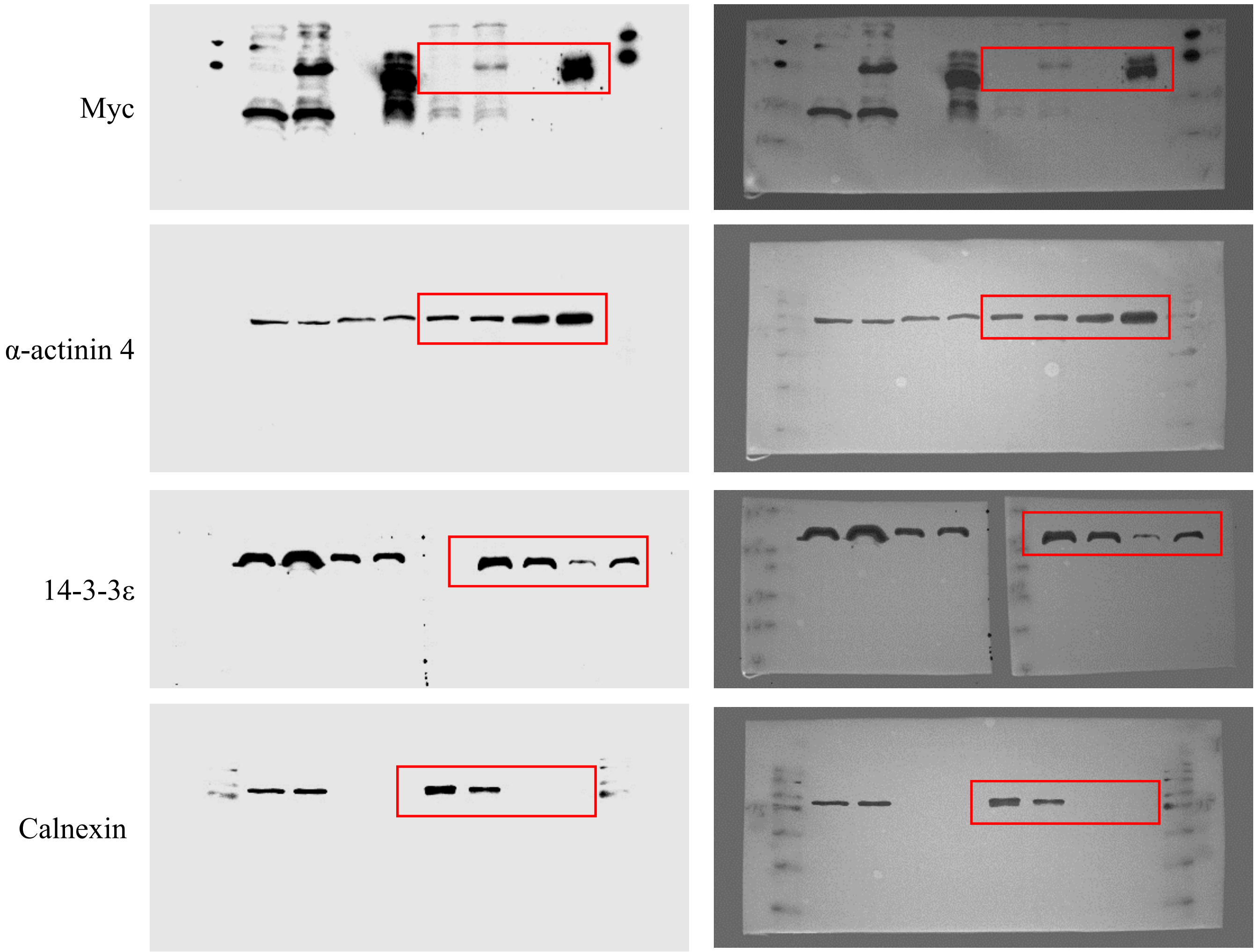
Myc
α-actinin 4
14-3-3ε
Calnexin

Fig. 4A

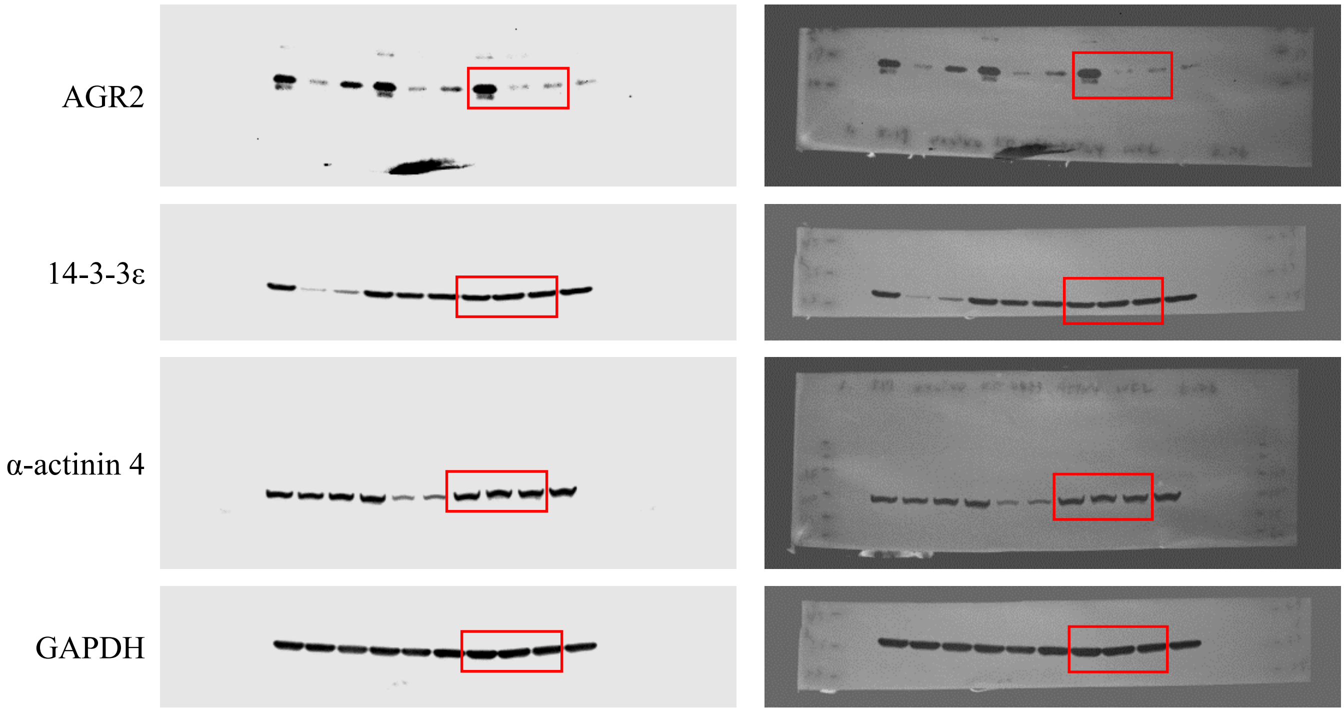

Fig. 4C

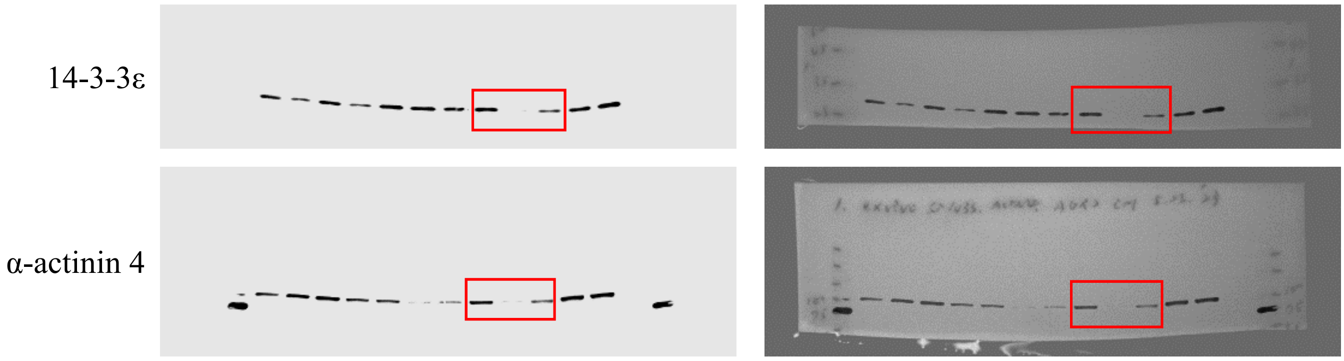

Fig. 4F

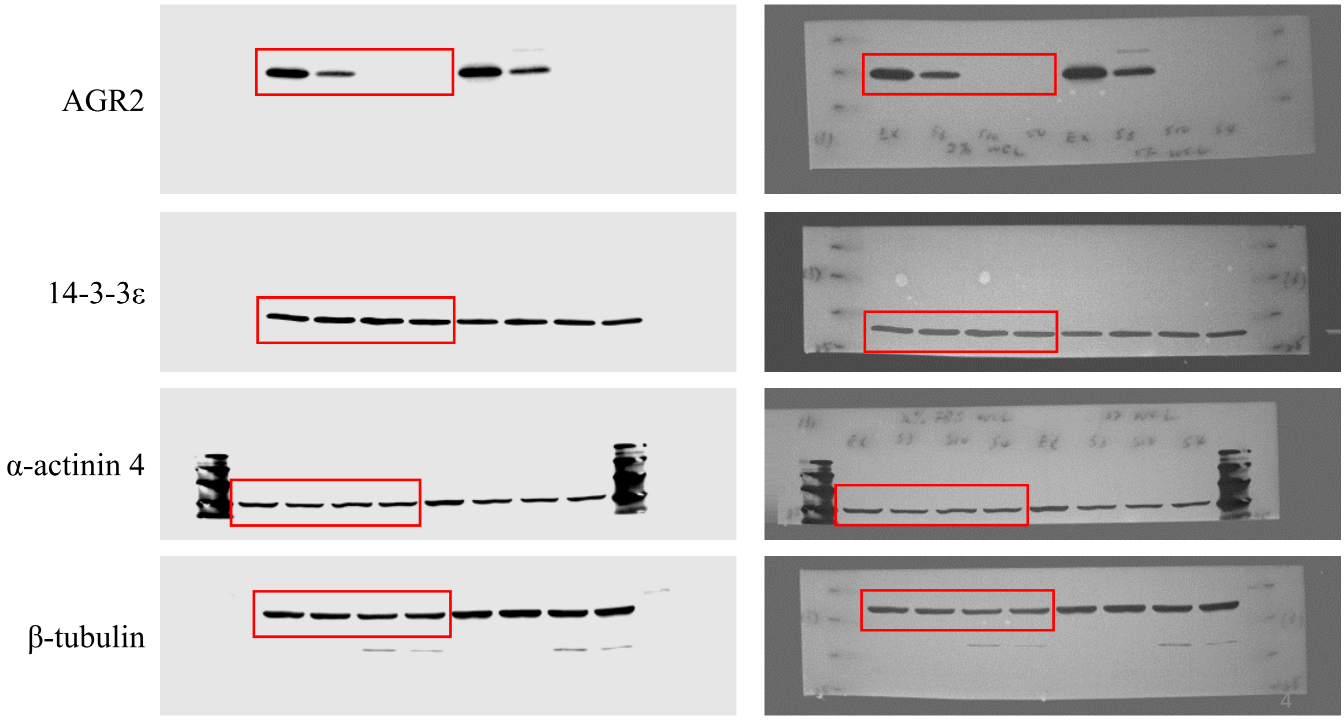

Fig. 4G

14-3-3ε

α-actinin 4

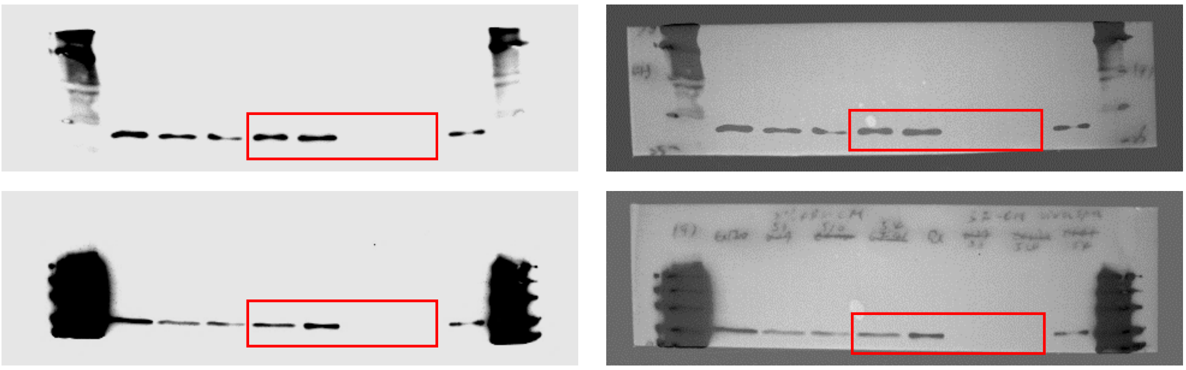

Fig. 5A

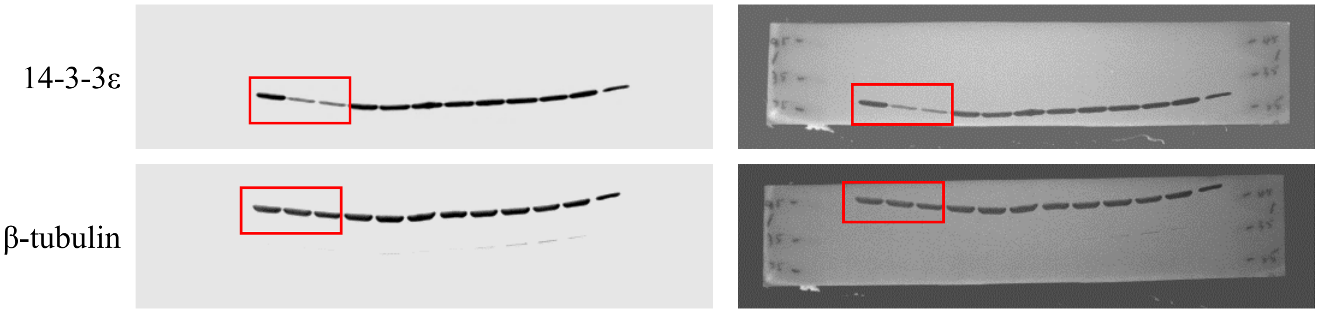

Fig. 5B

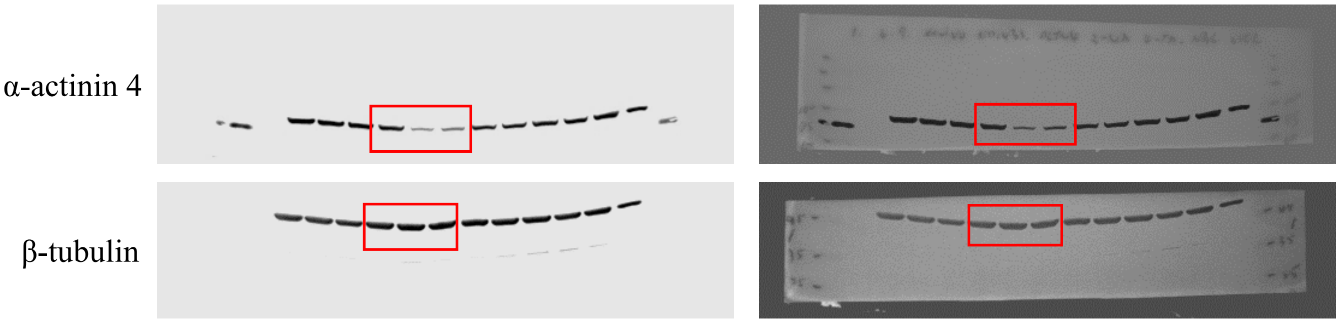

Fig. 5C

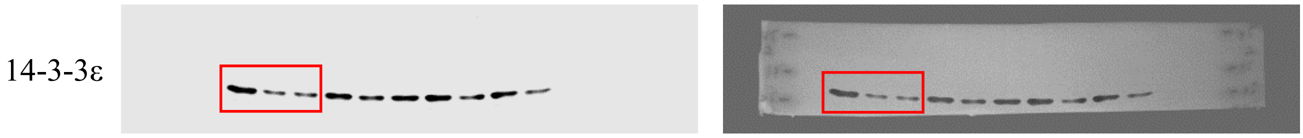

Fig. 5D

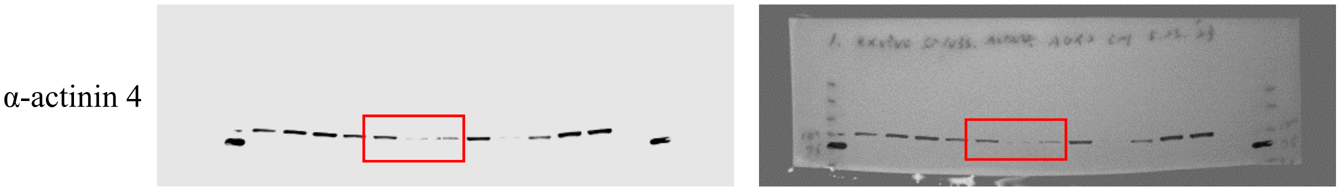

Fig. 5F

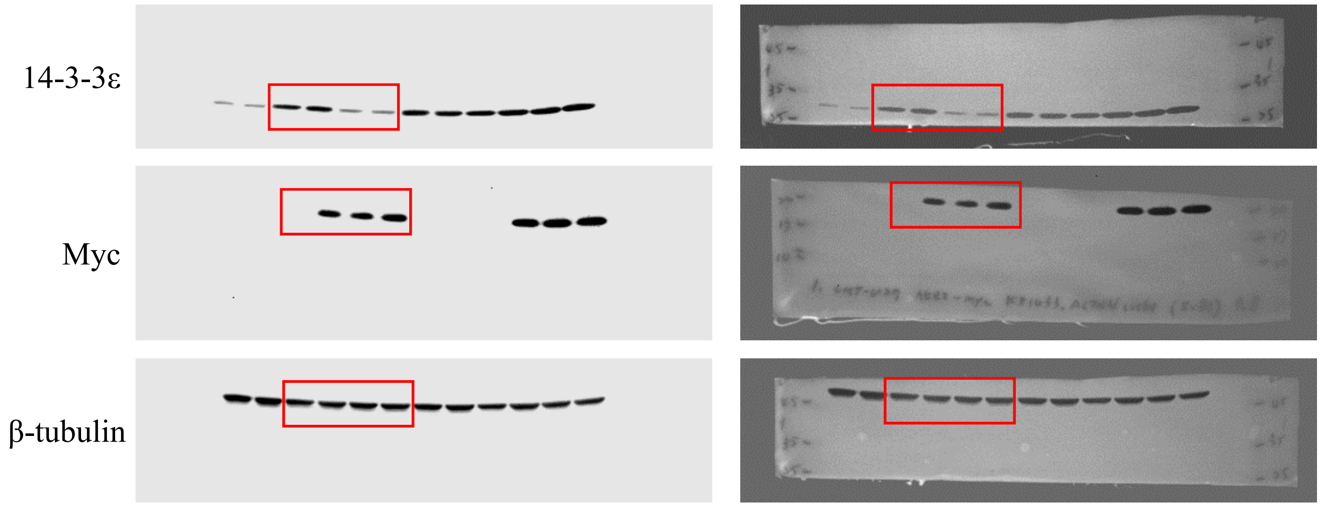

Fig. 5G

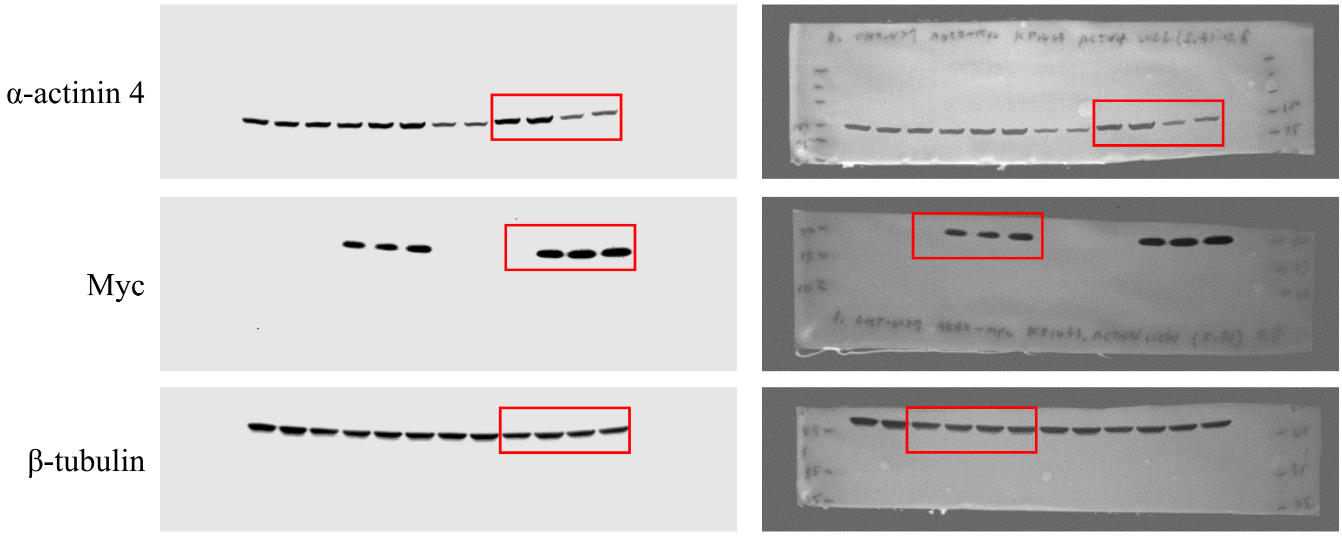

Fig. 5I

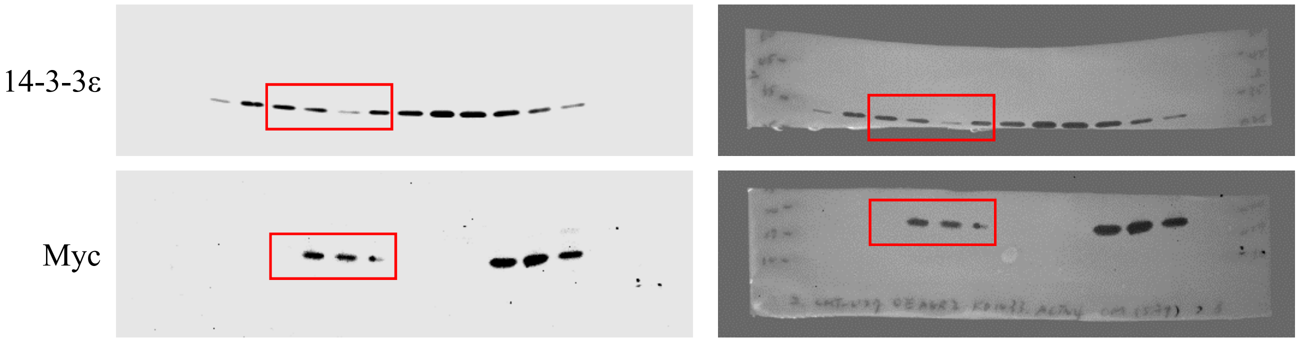

Fig. 5J

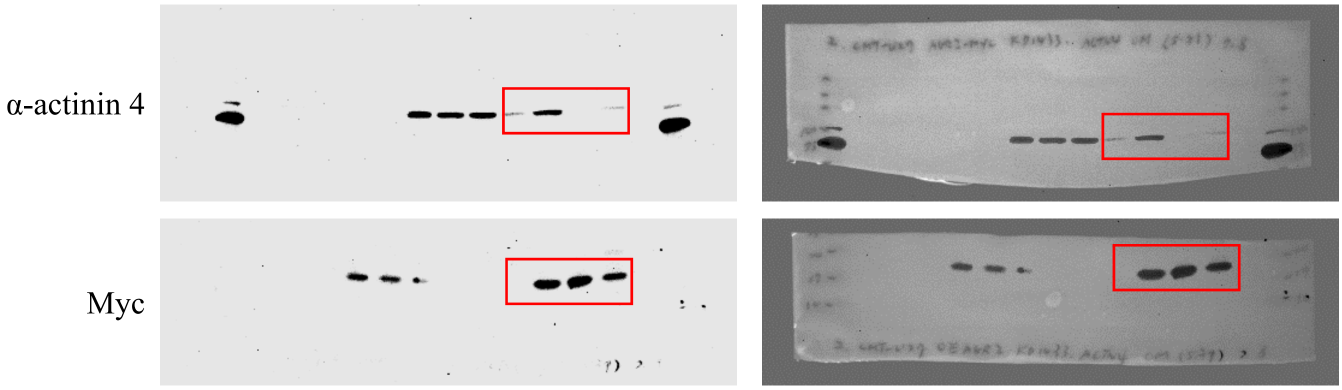

Fig. 6A

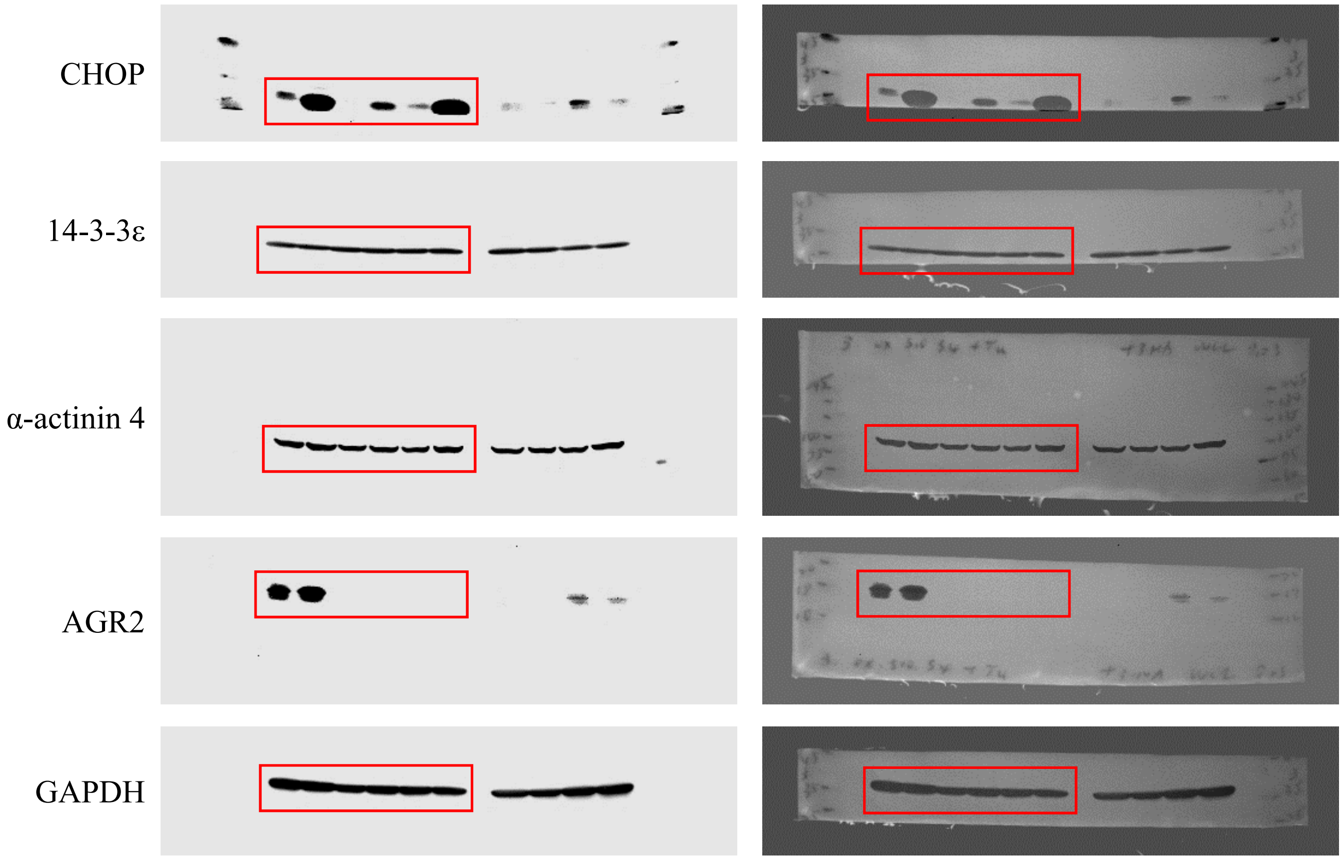

Fig. 6B

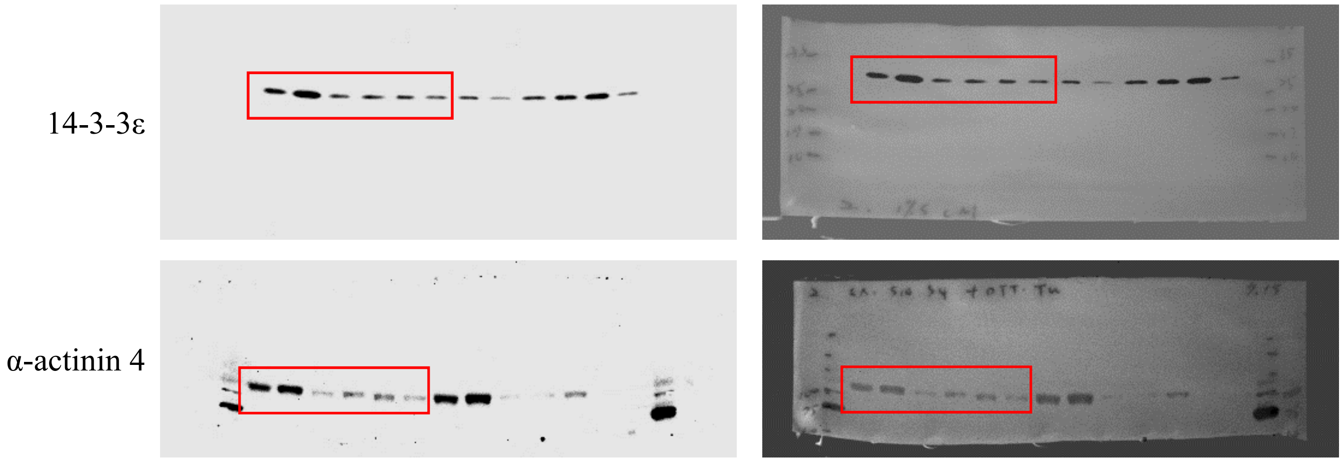

Fig. 6C

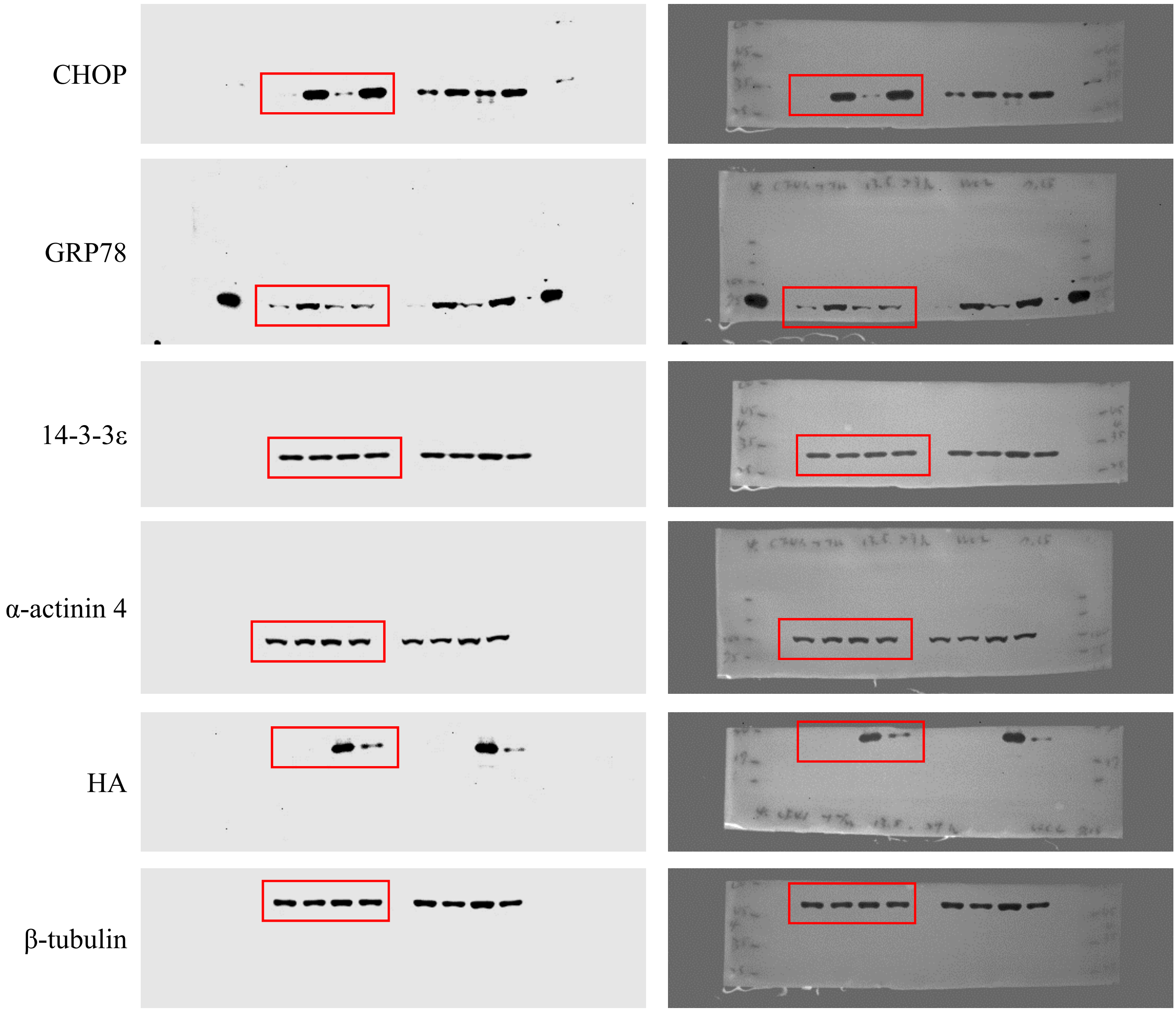

Fig. 6D

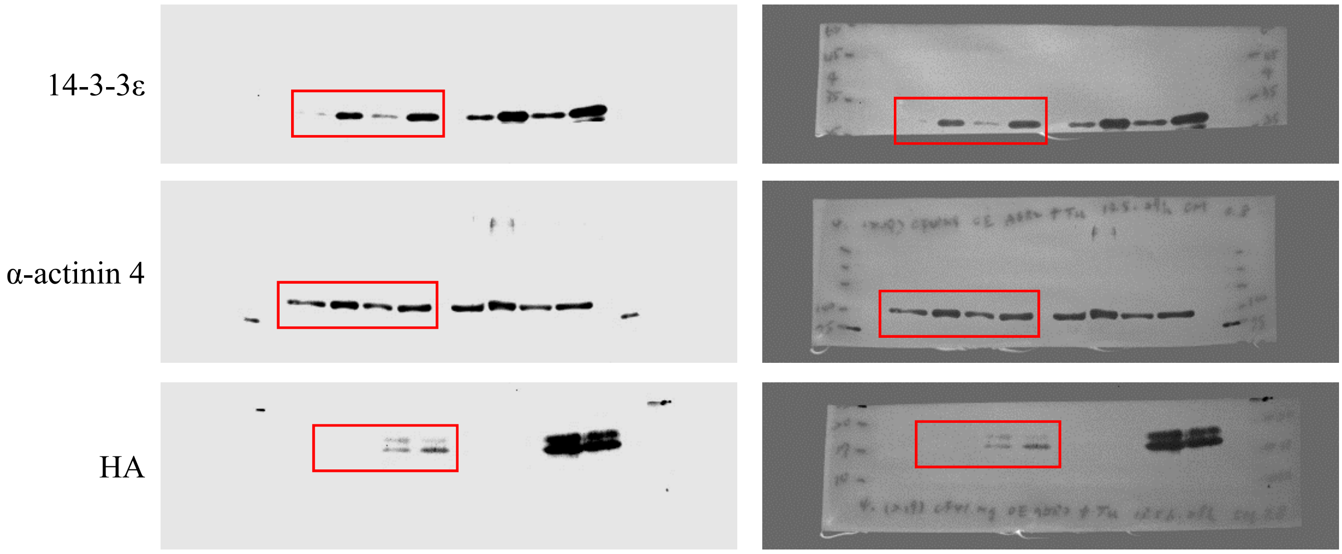

Fig. 6E

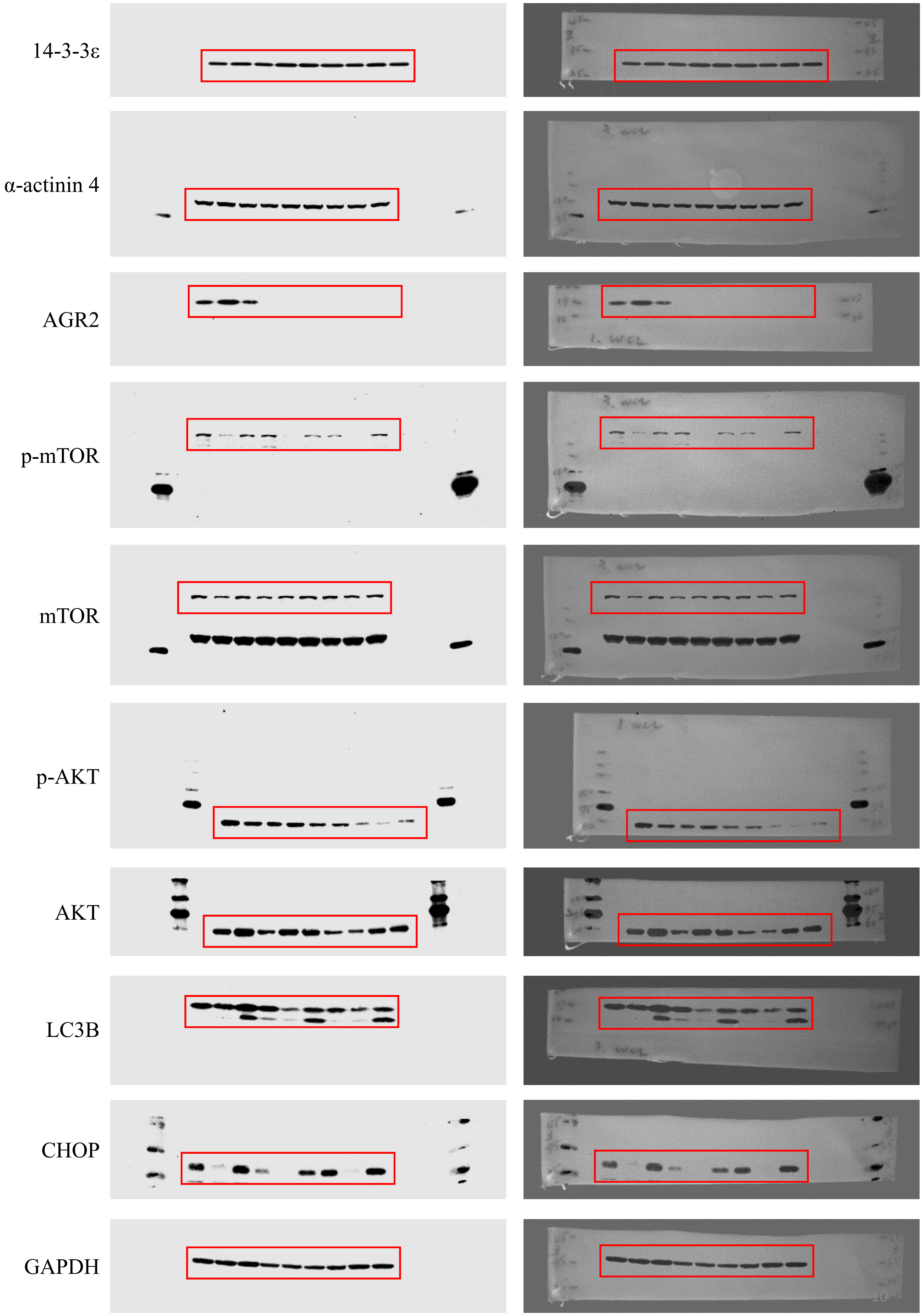

Fig. 6F

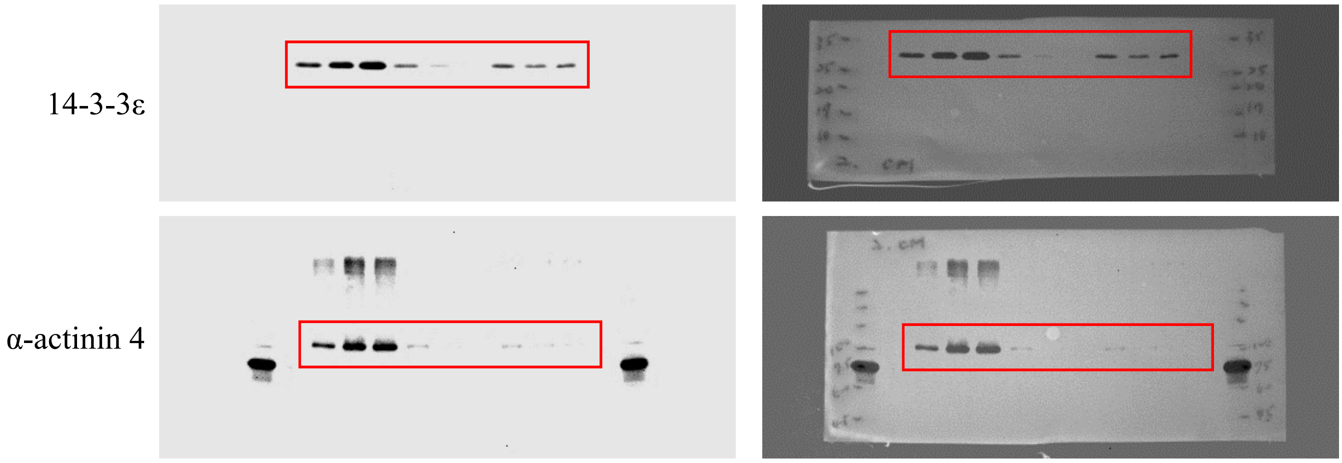

Fig. 6G

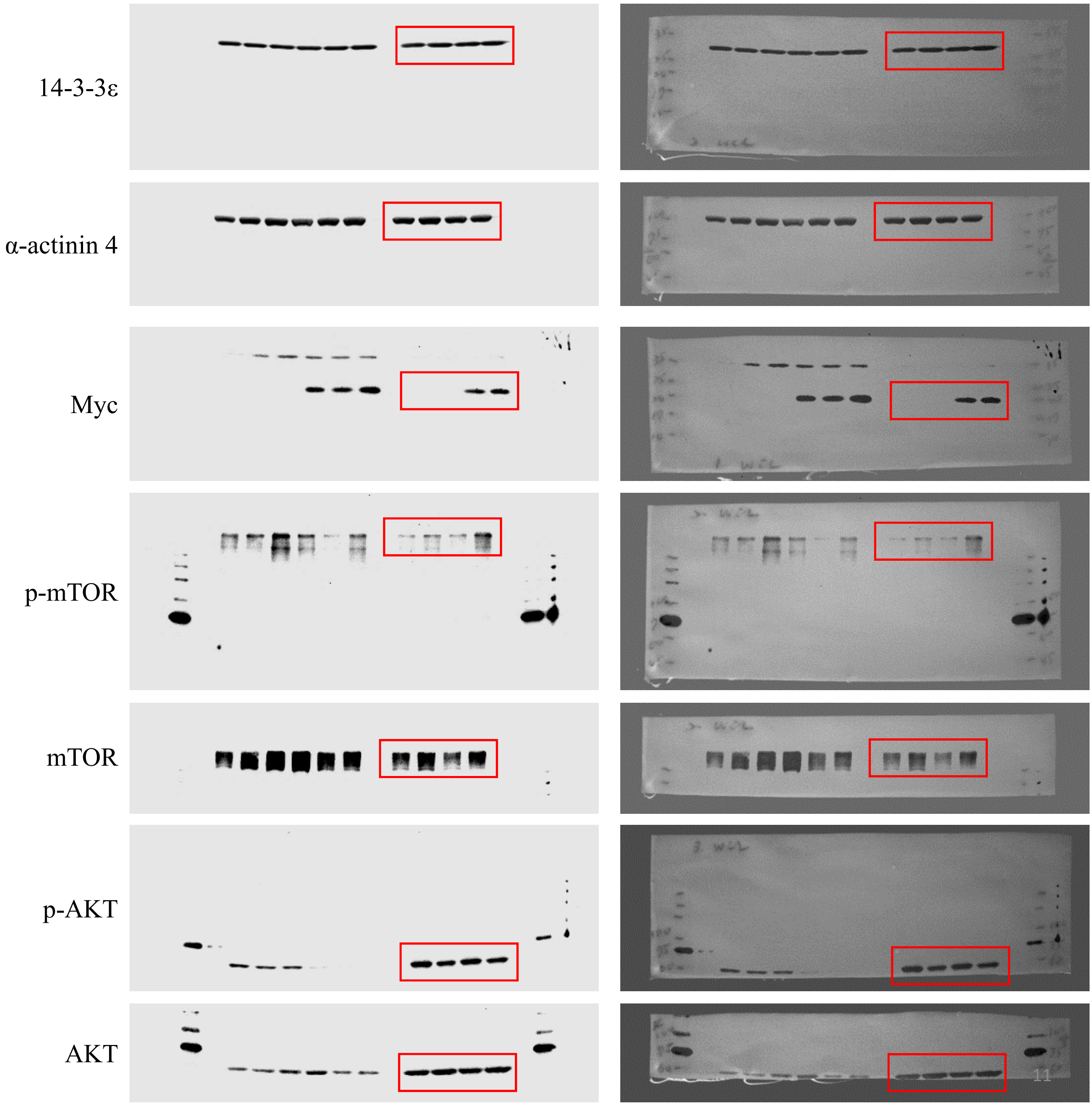

Fig. 6G

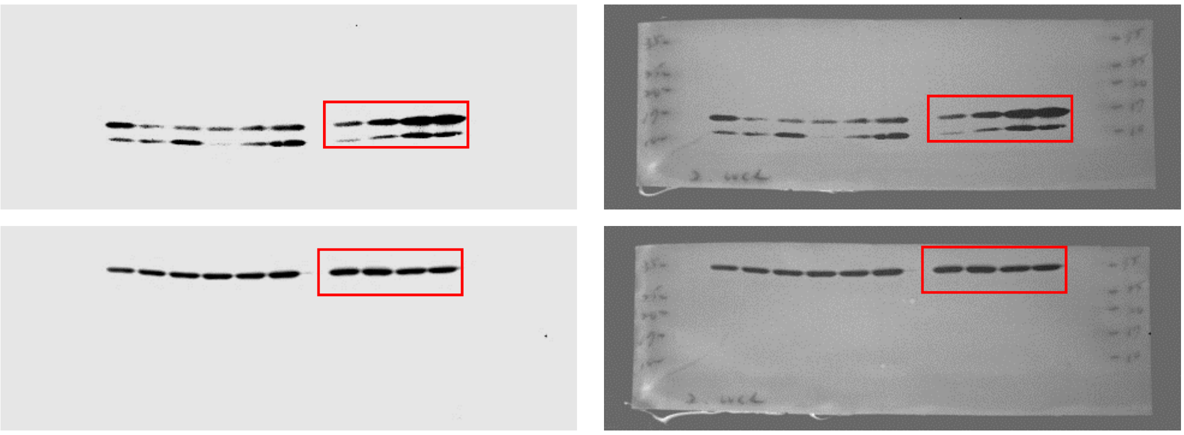

LC3B

GAPDH

Fig. 6H

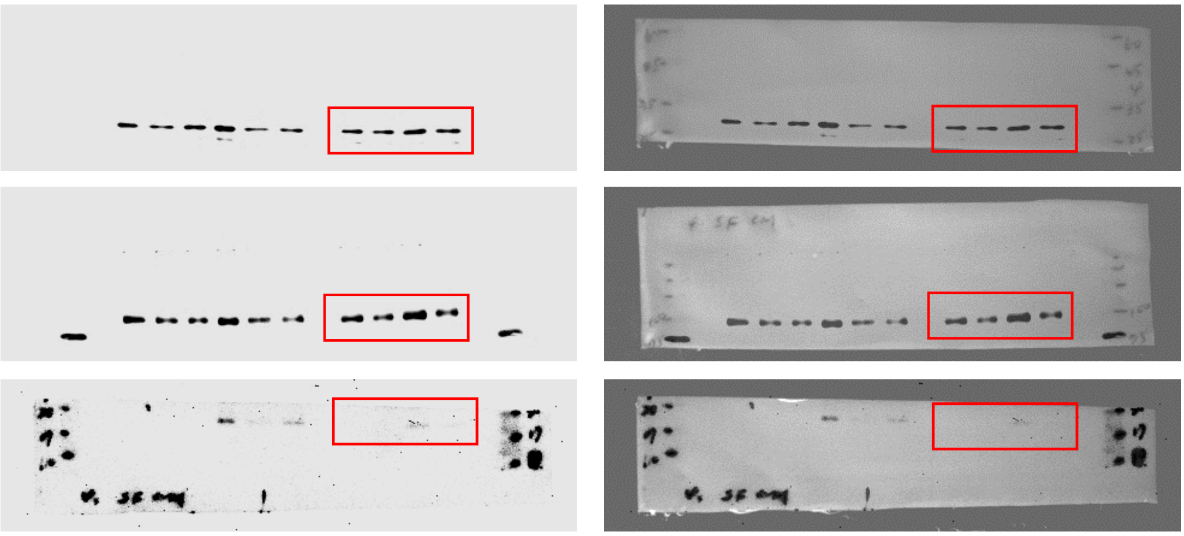

14-3-3ε

α-actinin 4

HA

Fig. 8A

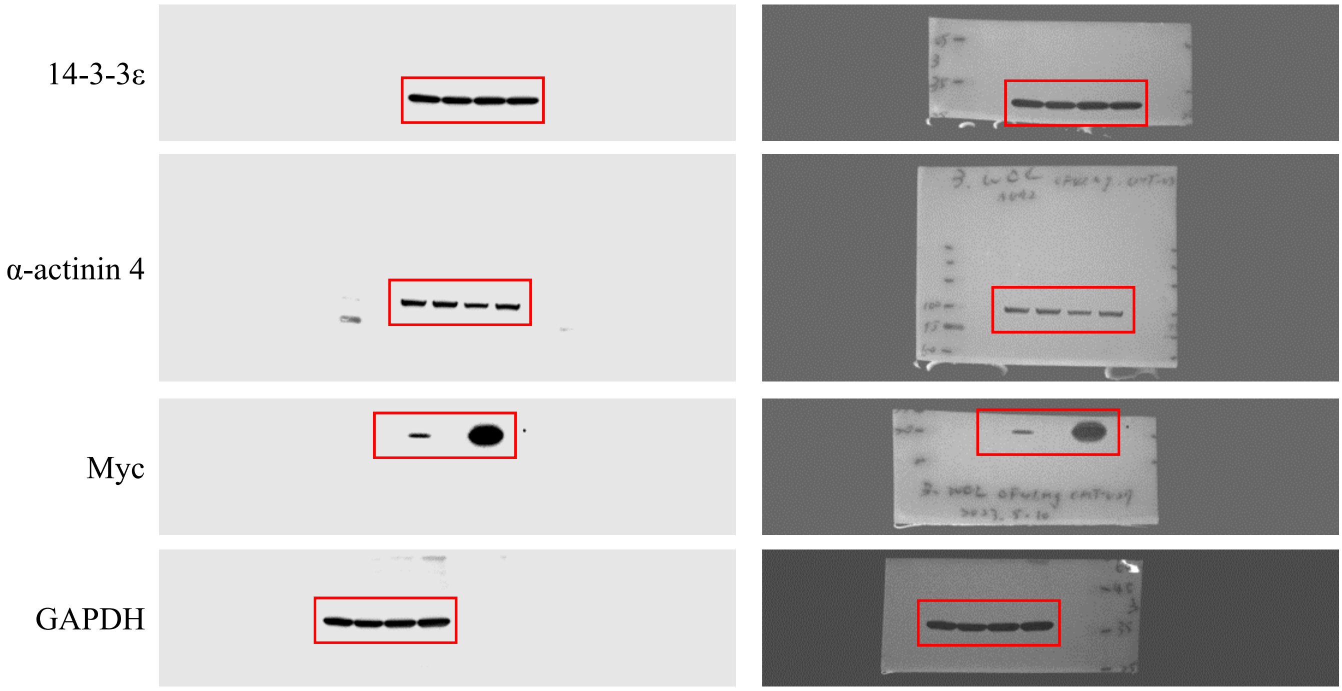

Fig. 8B

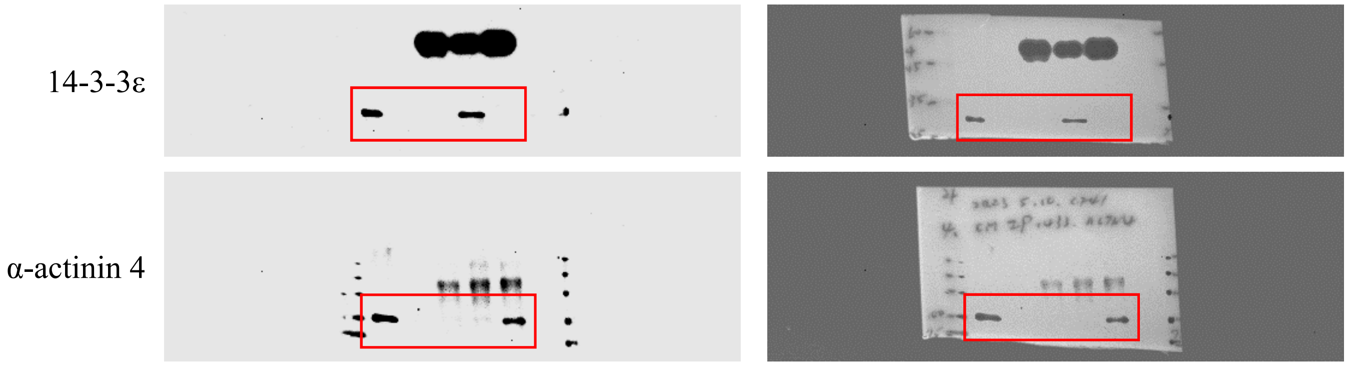

Fig. 8E

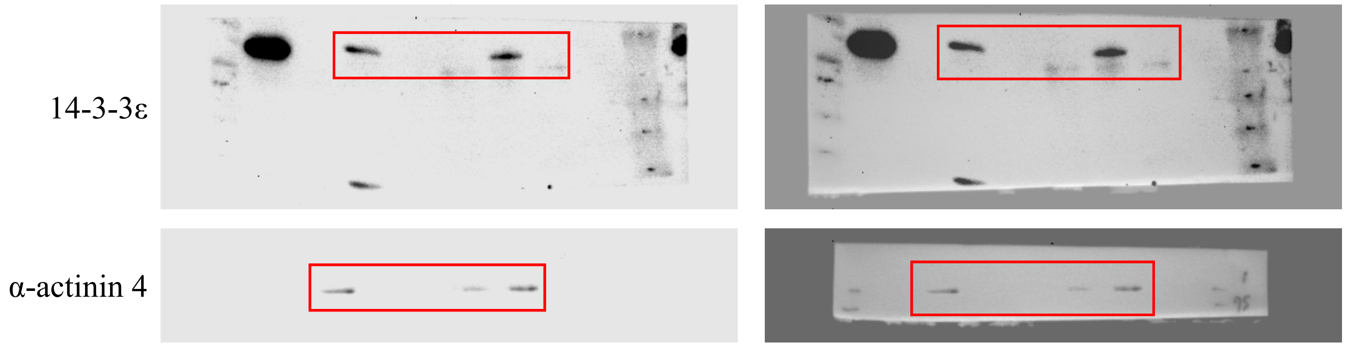

Fig. 9A

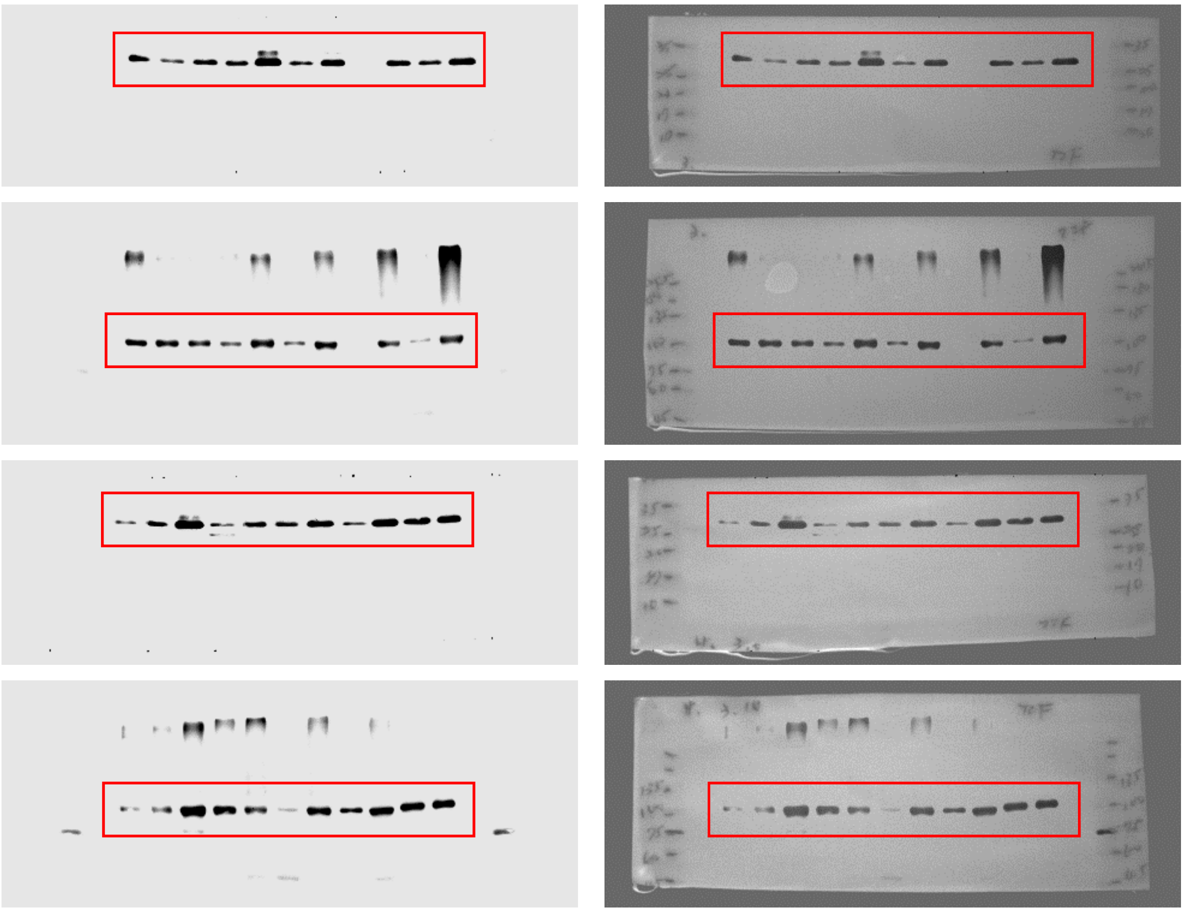

14-3-3ε

α-actinin 4

14-3-3ε

α-actinin 4

Fig. 9C

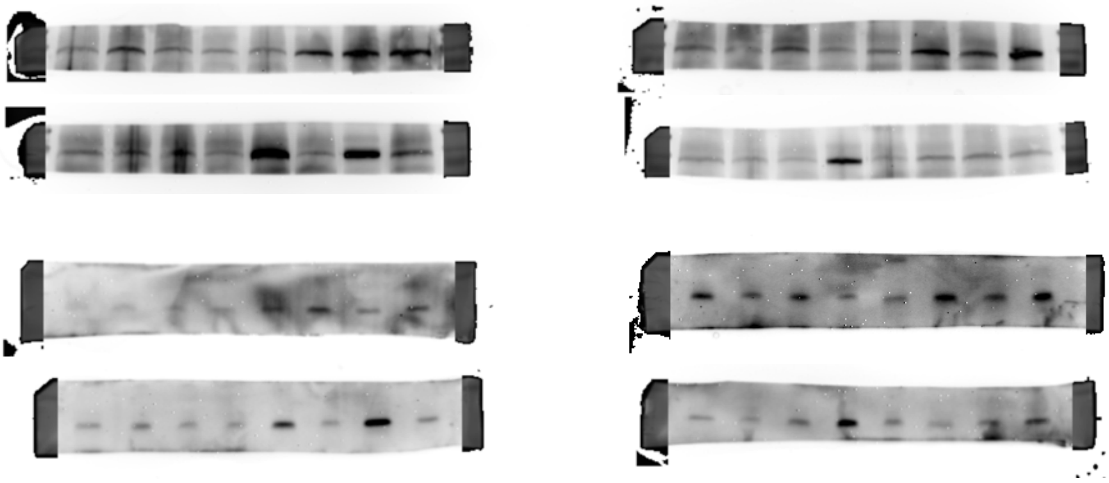

α-actinin 4

14-3-3ε

Fig. S1A

AGR2

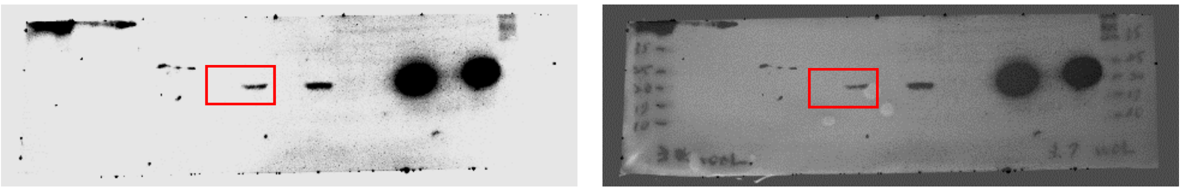

Fig. S1C

AGR2

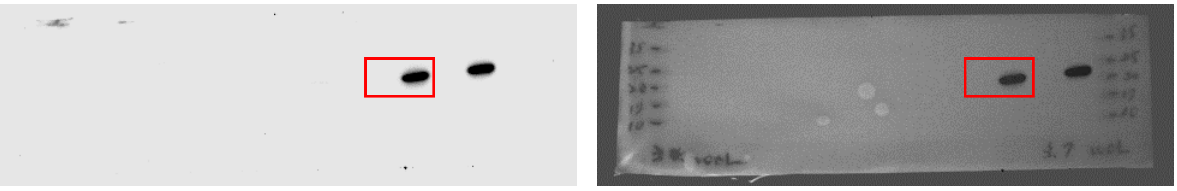

Fig. S1E

AGR2

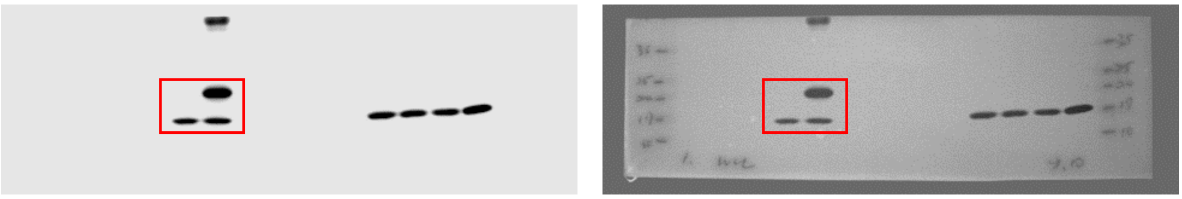

Fig. S2A

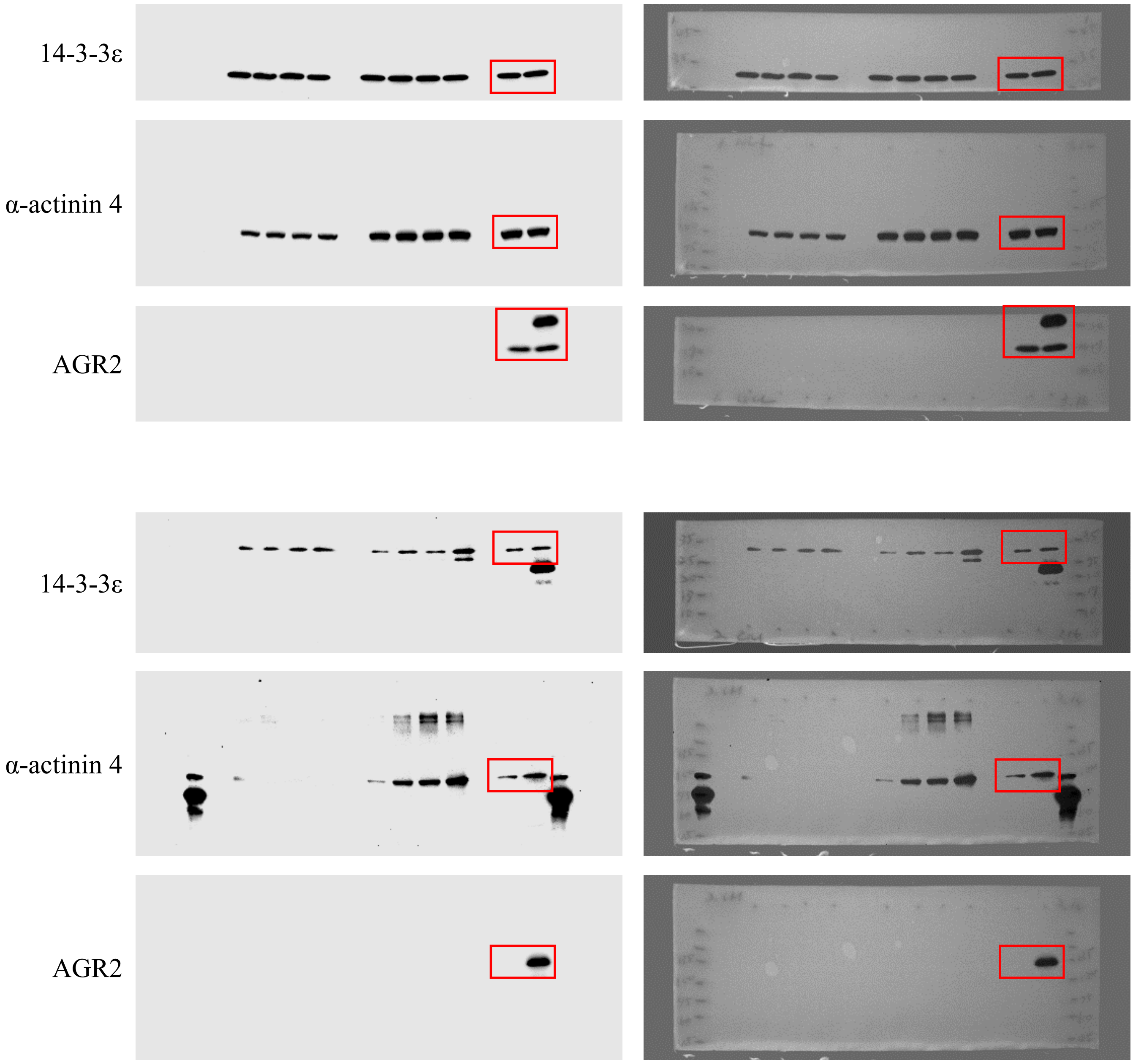

Fig. S2B

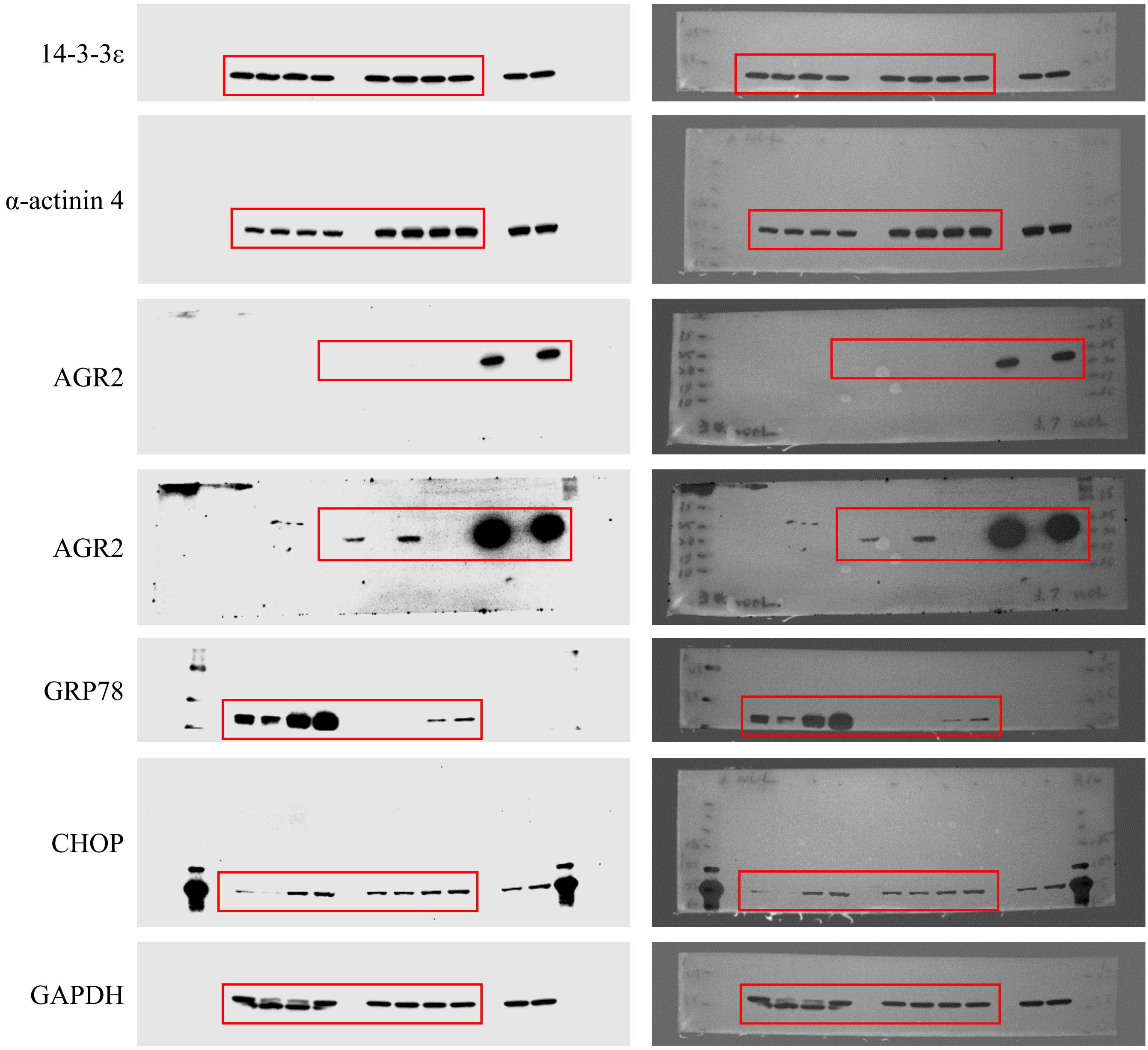

Fig. S2B

14-3-3ε

α-actinin 4

14-3-3ε

α-actinin 4

AGR2

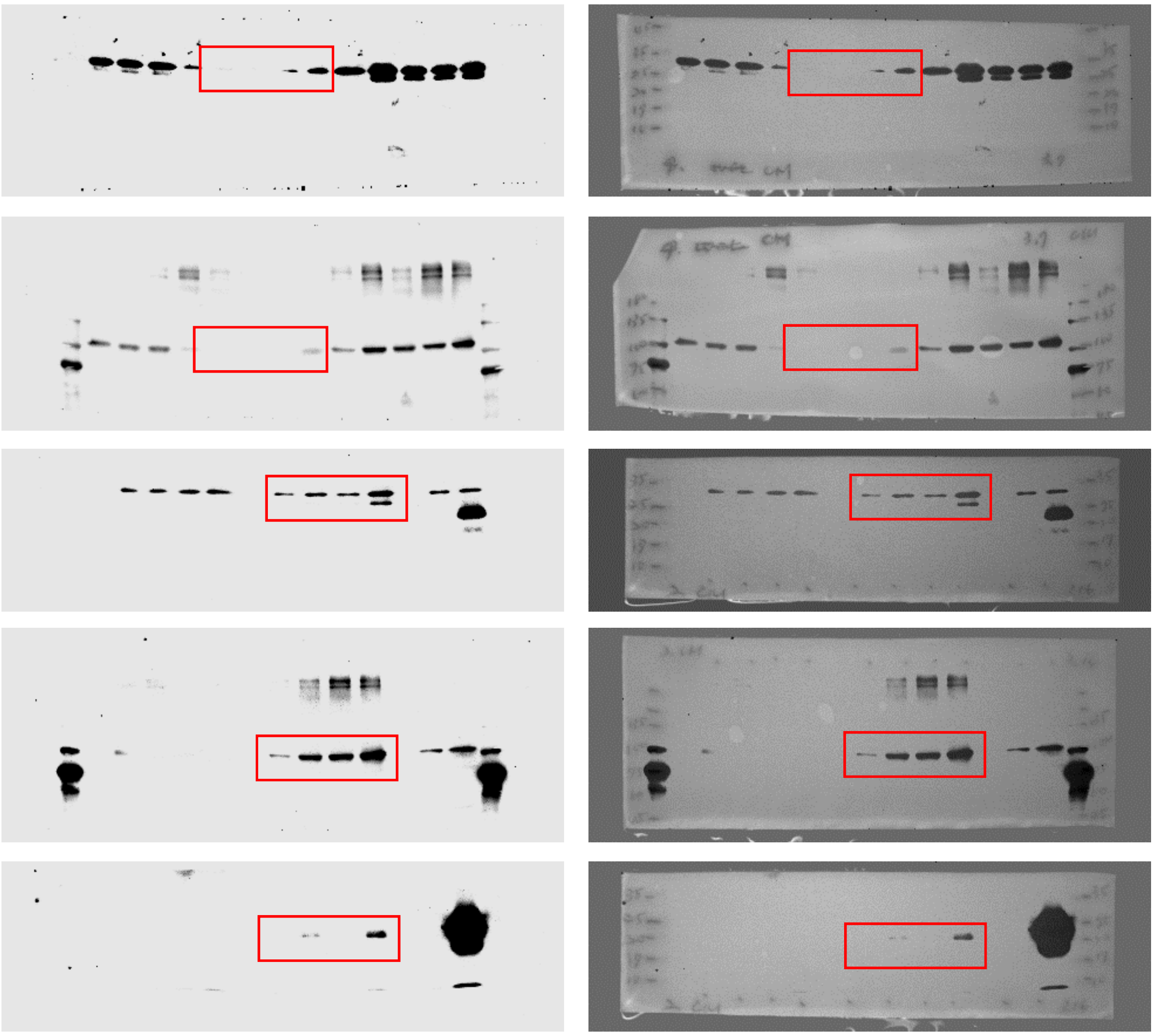

Fig. S2C

14-3-3ε

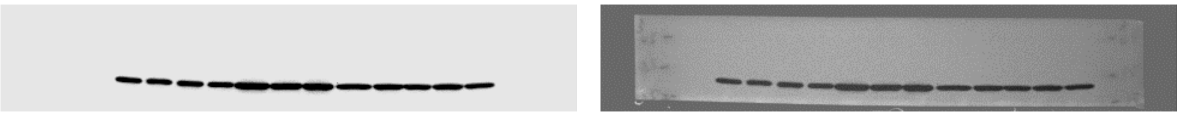

α-actinin 4

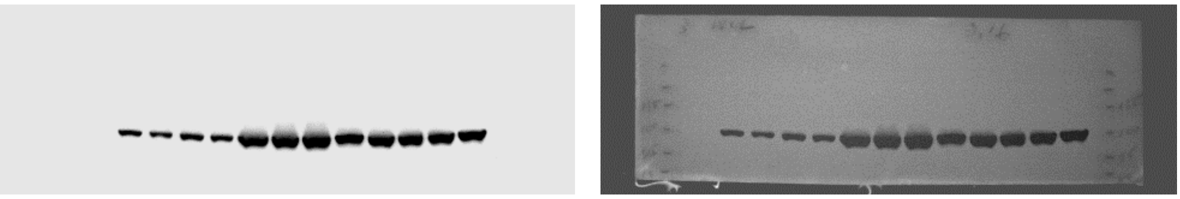

AGR2

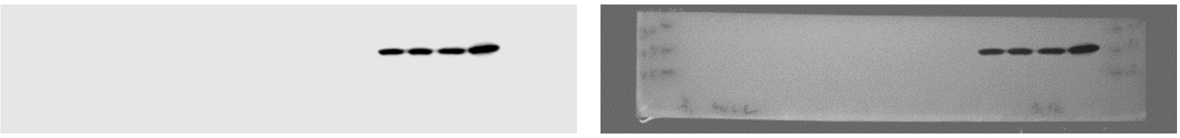

GRP78

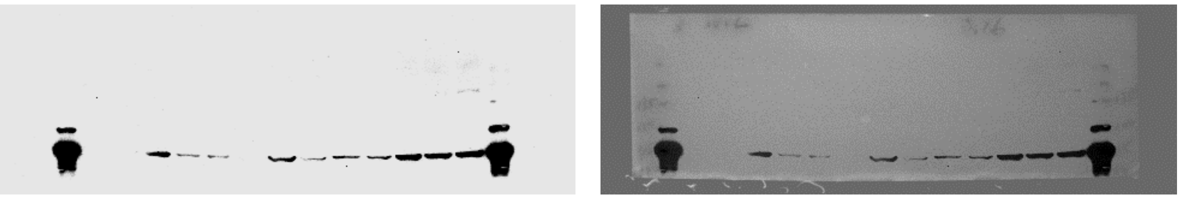

CHOP

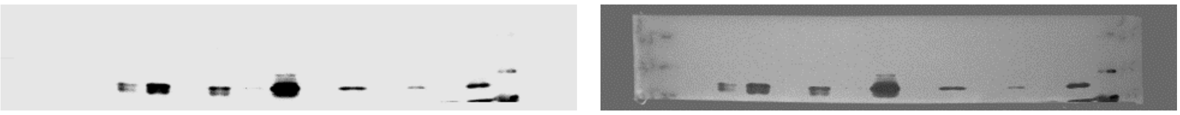

GAPDH

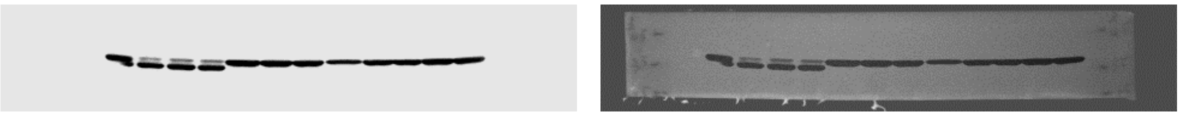

LC3B

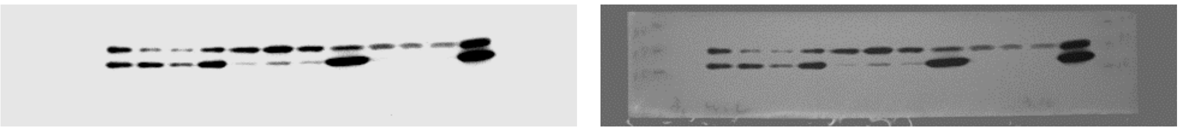

14-3-3ε

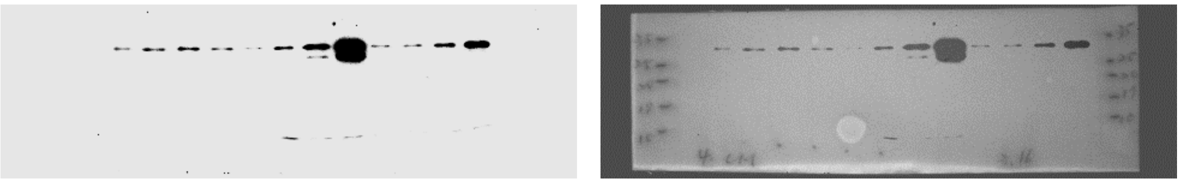

α-actinin 4

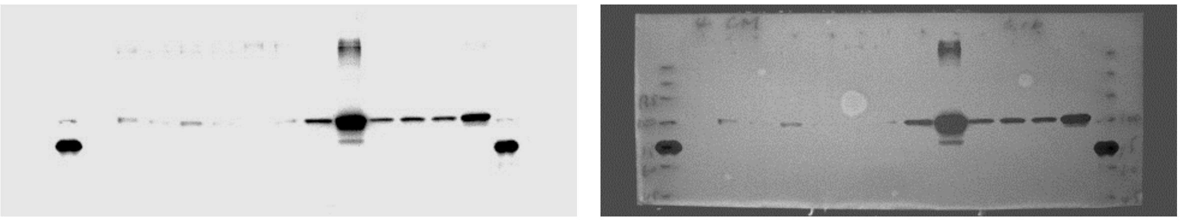

Fig. S3D

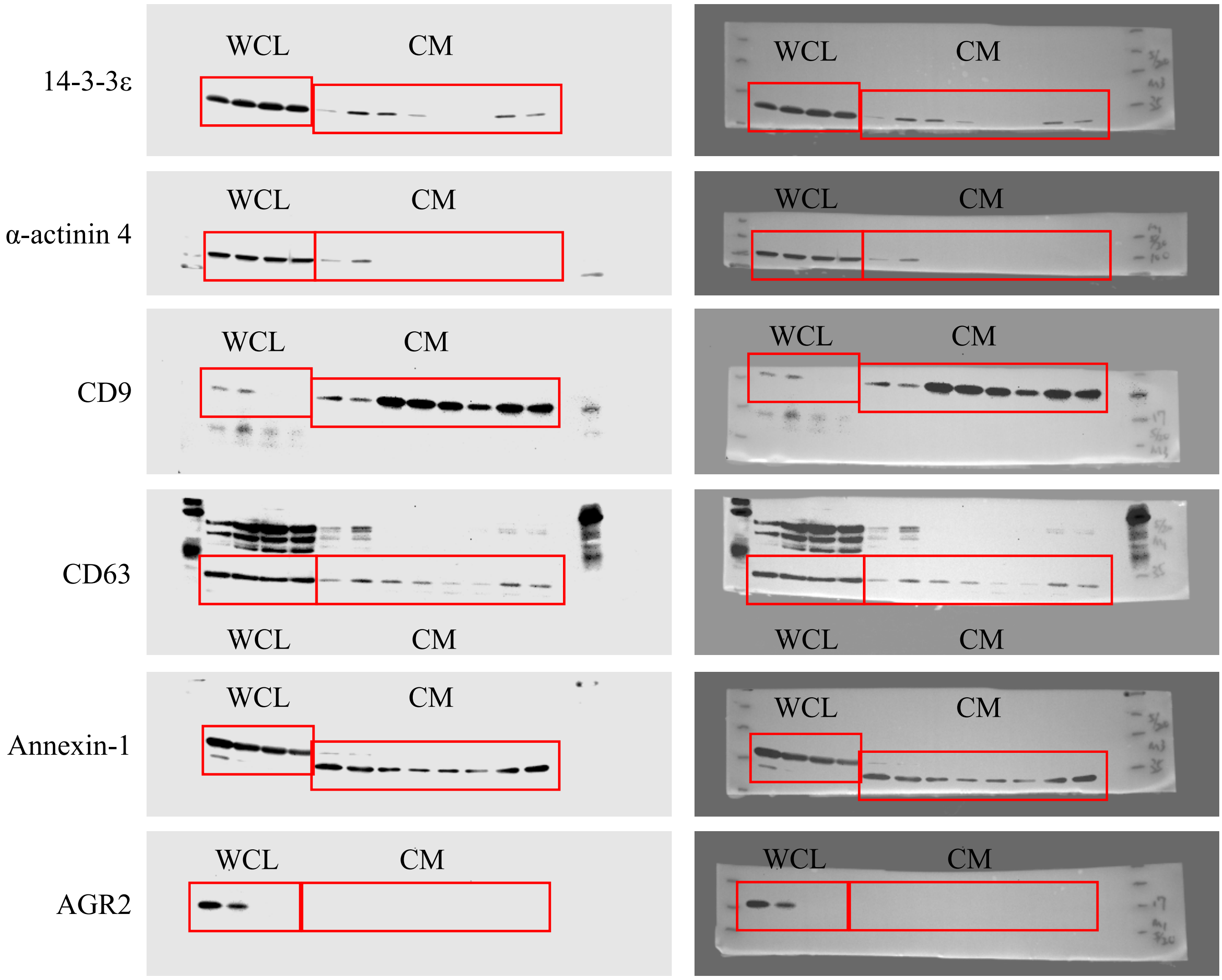

Fig. S3E

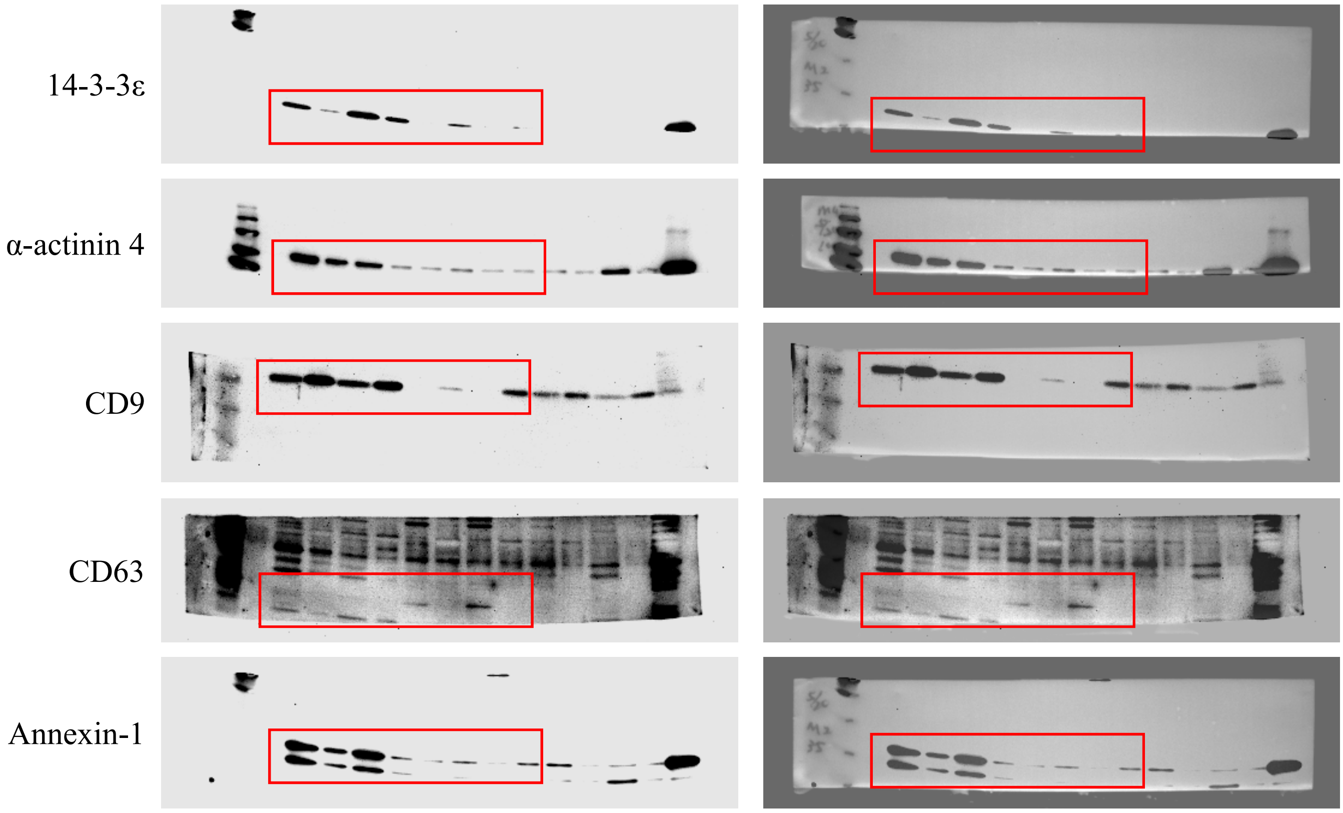

Fig. S4B

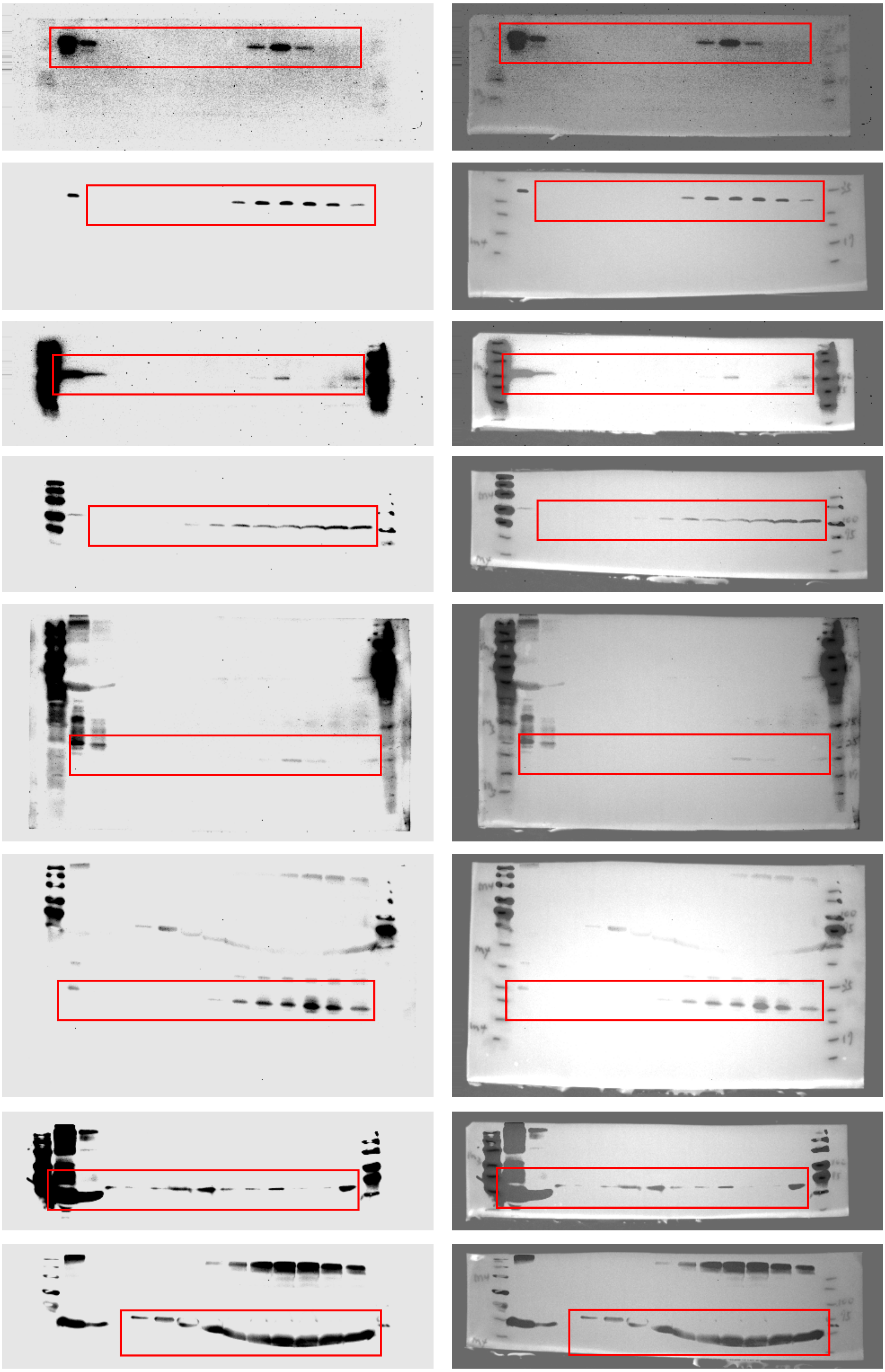

14-3-3ε

14-3-3ε

α-actinin 4

α-actinin 4

CD9

CD9

Albumin

Albumin

Fig. S4E

CMT-U27e

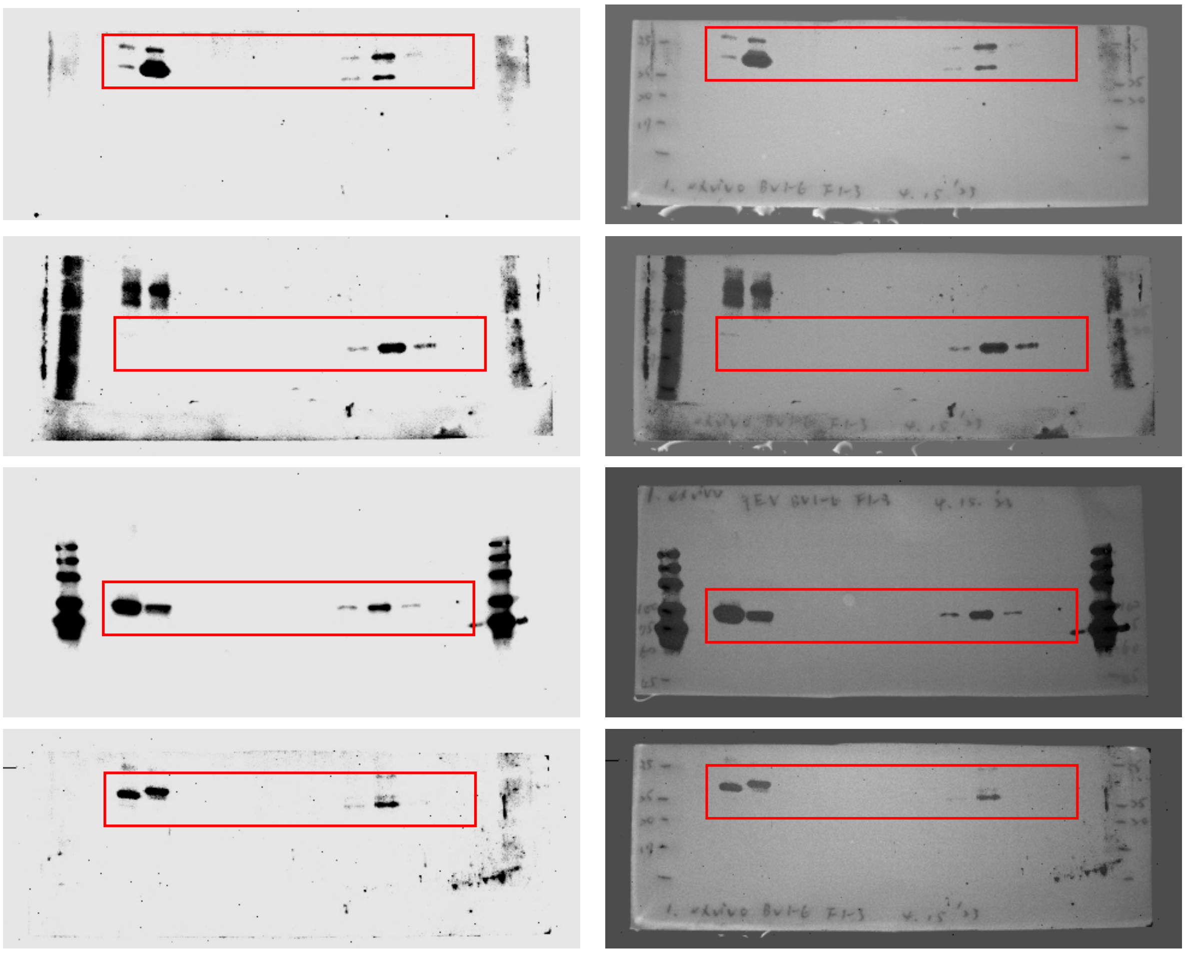

Annexin-1

CD9

α-actinin 4

14-3-3ε

Ctrl-S3

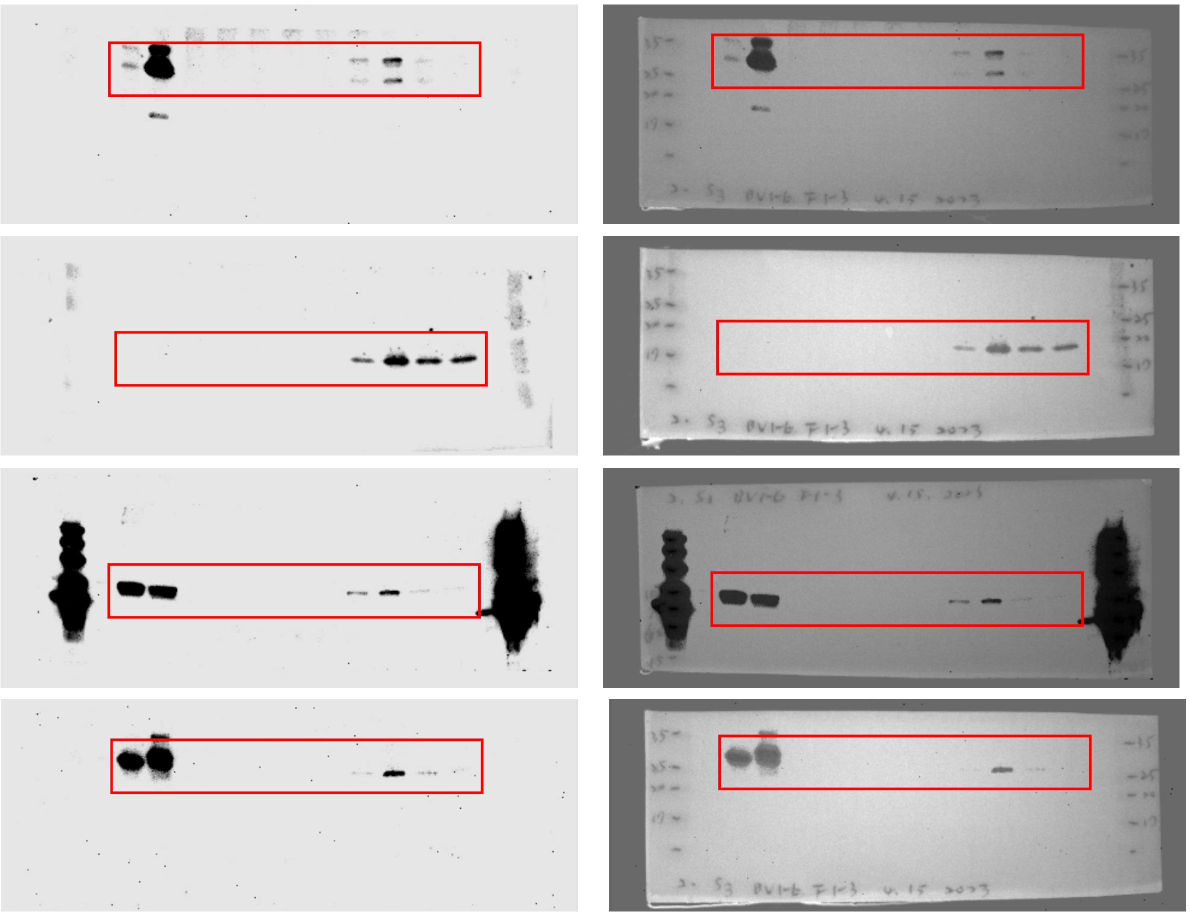

Annexin-1

CD9

α-actinin 4

14-3-3ε

Fig. S4E

KO-S4

Annexin-1

CD9

α-actinin 4

14-3-3ε

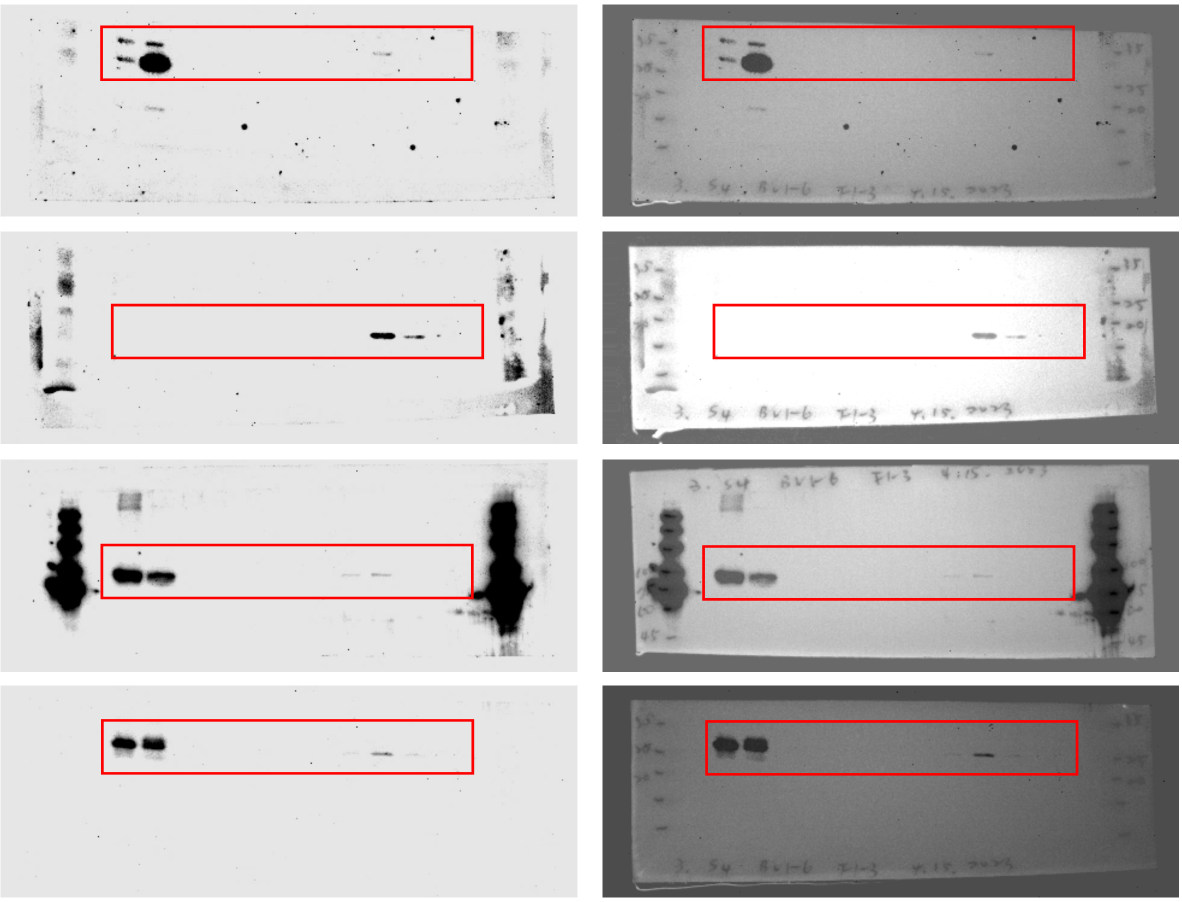

KO-S10

Annexin-1

CD9

α-actinin 4

14-3-3ε

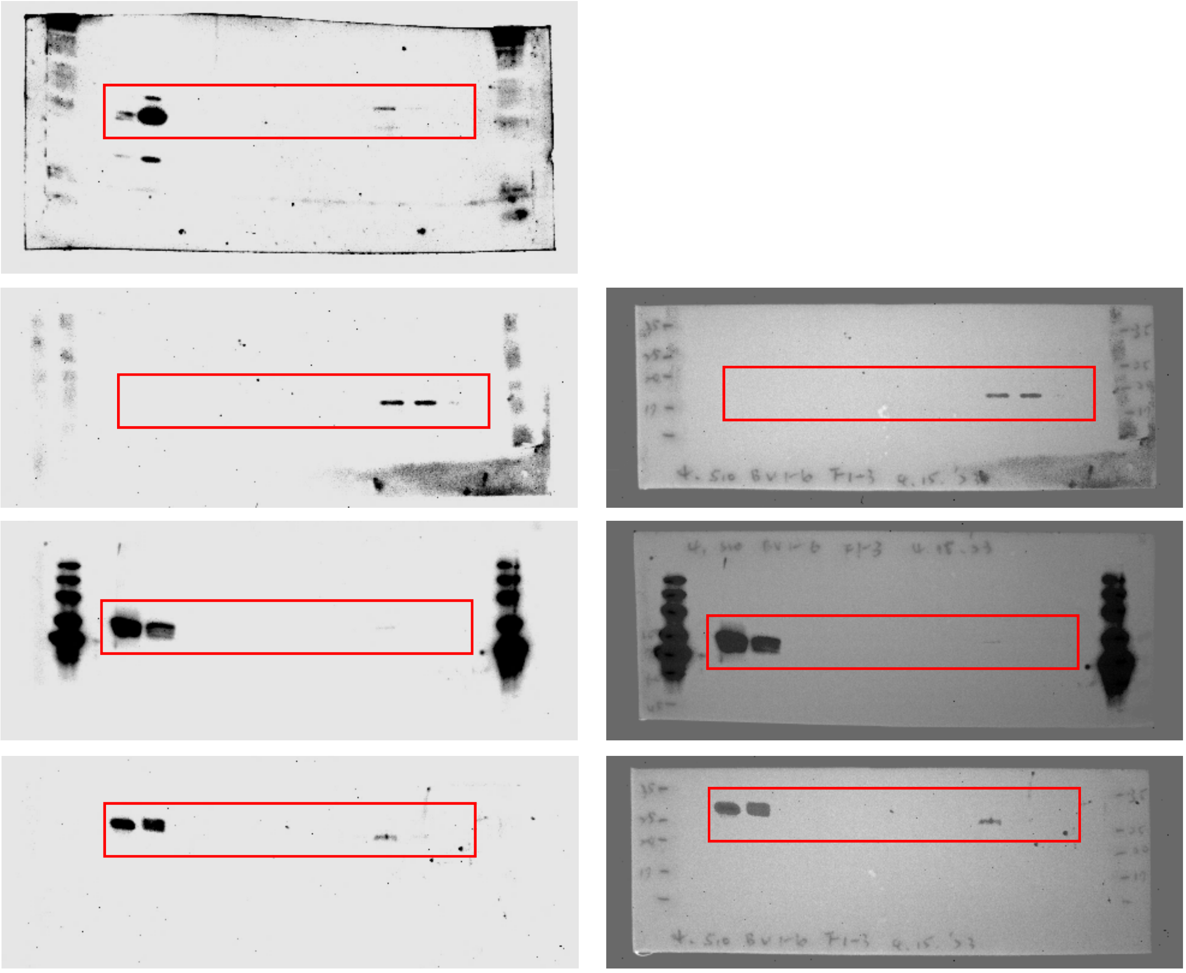

Supplement: Supplementary file 5 — Supplementary Material 5: Original immunoblots in this study. [file 11658_2024_601_MOESM5_ESM.pdf]
